# Comparative single-nucleus transcriptomics reveals asymmetric evolution of the *Drosophila* male and female germlines

**DOI:** 10.1371/journal.pbio.3003869

**Published:** 2026-07-20

**Authors:** Imtiyaz E. Hariyani, Sudeshna Das, Emma M. Le, Carmen Gamero-Castano, Tina Soroudi, Joshua Choi, Spring Momeni, Justin C. Kim, Rongying Lu, Vivek Swarup, José M. Ranz

**Affiliations:** 1 Department of Ecology and Evolutionary Biology, University of California Irvine, Irvine, California, United States of America; 2 Department of Neurobiology and Behavior, University of California Irvine, Irvine, California, United States of America; 3 Department of Systems Biology, University of California Irvine, Irvine, California, United States of America; Fred Hutchinson Cancer Research Center, UNITED STATES OF AMERICA

## Abstract

Reproductive organs vary widely across species yet share conserved cell types that produce gametes, sustaining species’ perpetuation. However, tissue-level comparisons mask critical differences among cell types, obscuring where evolutionary divergence occurs even between closely related species. We quantified expression divergence at cell-type resolution between two sibling species, *Drosophila melanogaster* and *D. simulans*, while disentangling adaptive and nonadaptive evolutionary mechanisms. We built a comparative single-nucleus transcriptomic atlas of over 100,000 nuclei from testes and ovaries of both species. Our analysis revealed sharply heterogeneous divergence across testis cell types, contrasting with a broader conservation across ovary cell types. Notably, in both organs, ~40% of genes showing interspecific differences did so in only one cell type. In the testis, spermatogonia were largely conserved, whereas divergence peaked in primary spermatocytes with extensive rewiring of coexpression modules linked to microtubule and mitochondrial functions. In the ovary, expression was largely conserved, except in early germline and late follicle cells, which showed shifts in oogenesis and cell-cycle-related coexpression modules. Divergent cell types in both tissues were enriched for evolutionarily young genes with narrow expression breadth and faster protein evolution rates. Additionally, the ovary exhibited a faster-*X* effect consistent with adaptive evolution. These findings reveal a fundamental asymmetry in how male and female germlines evolve, with functional constraints relaxed in specific testis cell types but broadly maintained across the ovary. Our work provides an evolutionary framework explaining how core reproductive functions are safeguarded during species diversification while identifying germline cells that drive evolutionary change.

## Abstract 2

Reproductive organs from different species contain conserved features and cell types. By performing single-cell transcriptomic analyses on two Drosophila species, this study shows that evolutionary divergence occurs asymmetrically; specific cell types in the testis are hotspots of rapid gene expression change, while ovary cell types remain more broadly conserved.

## Introduction

Gene expression changes are a key driver of species adaptation and phenotypic diversification [1–4]. While early comparative transcriptomic studies using microarrays or bulk RNA-sequencing helped characterize large-scale expression differences at the tissue-level, they lacked cell type resolution and were confounded by differences in tissue composition between species [5,6]. Single-cell and single-nucleus RNA sequencing (scRNA-seq and snRNA-seq, respectively) overcome these limitations by enabling direct comparisons of homologous cell types at unprecedented granularity within complex tissues [7,8]. These approaches have led to more precise evolutionary inferences—for example, in mammalian tissues such as the testis [5,9] and various regions of the brain [10,11]—by clarifying which cell types are most conserved versus those contributing disproportionately to expression divergence.

The gonads are central to understanding expression evolution, as they harbor most sex-biased genes [12] and exhibit distinct patterns of gene expression [13–15]. *Drosophila melanogaster* has served as a premier model organism for studying gametogenesis, primarily using bulk transcriptomic data, often derived from whole-body samples [16–21]. More recently, single-cell studies in *Drosophila* have revealed stage- and cell-type-specific expression patterns across both testis and ovary [7,22–30]. For example, DNA damage response genes are enriched in early germline stages of both gonadal tissues [22,27], and late spermatogenesis is marked by an enrichment of de novo genes [22]. However, reliance on single-strain datasets has limited our ability to capture the intraspecific genetic diversity of *D. melanogaster* [31–33], which is crucial for understanding functional constraints and identifying expression changes that truly contribute to species divergence [34,35]. Key questions remain unresolved: which gonadal cell types drive interspecific divergence? Do the testis and ovary follow parallel evolutionary trajectories, or do their constraints and modes of evolution fundamentally differ? What coexpression networks and functional pathways underlie this divergence? And do chromosomal location or genomic features explain putative cell-type-specific patterns?

Despite evolutionary divergence, gametogenesis in *Drosophila* and mammals shares conserved developmental features, including similar differentiation stages and core molecular pathways [36–38]. In mammals, transcriptome divergence is not uniform across the testis: it peaks in meiotic and post-meiotic germ cells of the testis, while early germline cells remain highly conserved—likely due to strong functional constraints tied to their pleiotropic roles [9,39,40]. In the mammalian ovary, the theca cells—endocrine somatic cells that surround the follicle—accumulate greater expression divergence between species compared to other ovarian somatic cell types [41]. Whether *Drosophila* exhibits similar patterns of stage- or cell-type-specific divergence in either gonadal tissue remains unknown. Comparative single-cell analyses of closely related *Drosophila* species remain scarce, making it difficult to discern whether previously reported expression divergence reflects true biological differences or methodological artifacts such as confounding or compensatory effects [13,16–18]. If substantial interspecific expression differences exist in *Drosophila*, it is unclear whether they are broadly distributed across cell types or concentrated in terminally differentiated stages, similar to patterns observed in the mammalian testis.

To address these questions, we used snRNA-seq to compare the testis and ovary transcriptomes of three strains spanning two closely related species, *D. melanogaster* and *D. simulans*, which diverged ∼1.4 mya [42]. We characterized expression divergence across cell types by integrating two complementary analyses: differential expression at the level of individual genes and network divergence at a systems level. We also examined how differentiation trajectories in spermatogenesis and oogenesis relate to pleiotropic constraint, using proxies such as expression breadth and phylogenetic gene age. Our results reveal a dichotomy in the tempo and mode of transcriptome evolution between the male and female germline. Testis transcriptomes evolve rapidly and in a highly cell-type-specific manner, with divergence in meiotic cells driven by a combination of adaptive and nonadaptive mechanisms. In contrast, the ovary exhibits more conserved expression properties, consistent with stronger pleiotropic constraint—except in a subset of germline and late-stage follicle cells, which show pronounced divergence. We further show that the cell types accumulating divergent genes also tend to have narrow expression breadth and younger phylogenetic age, especially in the testis, and that divergent genes are more likely to be on the *X* chromosome in the ovary. Together, these findings uncover how distinct evolutionary mechanisms shape gene expression programs across cell types in the male and female germlines.

## Results

### Combinatorial barcoding enables cell-type-resolved gonadal transcriptomes across *Drosophila* species

To characterize gene expression divergence between closely related *Drosophila* species at cell-type resolution, we performed single-nucleus RNA-sequencing (Parse Biosciences Evercode v2 [43]) on gonadal tissues from two strains of *Drosophila melanogaster* (A4, ISO1) and one strain of *D. simulans* (*w*^*501*^), selected to capture intra- and interspecific divergence within the *D. melanogaster* species subgroup. We profiled testes from 1 to 2-day-old naïve males and ovaries from 3–day-old virgin females using split-pool combinatorial barcoding [43]. After quality control and doublet removal, we retained 106,328 high-quality nuclei–40,274 nuclei from testis and 66,054 nuclei from ovary–across 12 biological samples (3 strains × 2 tissues × 2 biological replicates). To minimize mapping artifacts due to nucleotide differences, we aligned sequencing reads to strain-specific genome assemblies. We then integrated cross-strain data for each tissue using Harmony [44], which best preserved cell-type and species structure among the tools tested (Methods). With an average of 44,742 reads per nucleus (S1 Table), our dataset is comparable to or exceeds those of previous studies (S2 and S3 Tables), enabling high-resolution comparisons of conserved and divergent expression patterns within and between species.

### Cellular composition of *Drosophila* gonads is conserved across species

**Fig 1:**
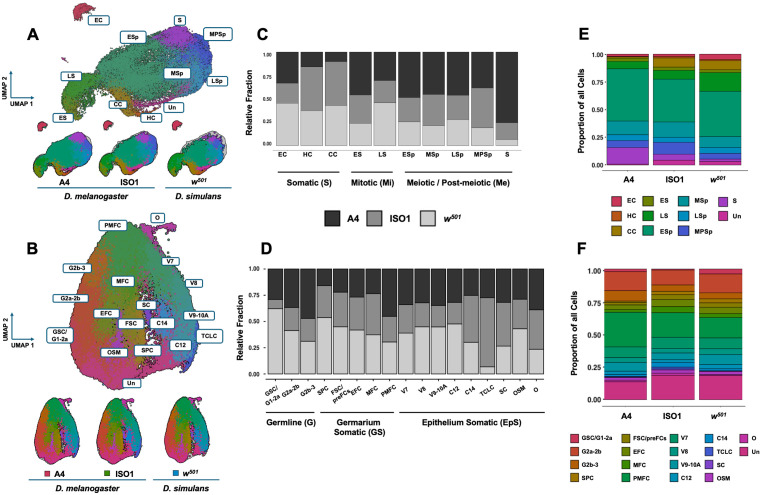
Single-nucleus transcriptomic landscapes of *Drosophila* testis and ovary across strains. UMAP visualization of transcriptomic profiles from the **(A)** testis and **(B)** ovary, respectively, colored by annotated cell types. Each cluster represents a distinct cell type that corresponds to a particular stage during spermatogenesis and oogenesis, respectively, combined for all three strains (above) and individually in *D. melanogaster* strains A4 and ISO1, and *D. simulans* strain *w*^*501*^ (below). Stacked bar plot showing the relative fraction of identified cell types for each strain in the **(C)** testis and **(D)** ovary, respectively. Stacked bar plot showing the proportion of all cells annotated for each identified cell type across the three strains in the **(E)** testis and **(F)** ovary, respectively. All annotated cell types were included after quality control filtering, regardless of number of nuclei. Testis cell types: EC, epithelial cells; HC, hub cells; CC, cyst cells; ES, germline stem cells and early spermatogonia; LS, late spermatogonia; ESp, early spermatocytes; MSp, mid spermatocytes; LSp, late spermatocytes; MPSp, maturing primary spermatocytes; and S, spermatids. Ovary cell types: GSC/G1-2a, germline stem cells and germarium region 1 and 2a cells; G2a-2b, germarium region 2a and 2b cells; G2b-3, germarium region 2b and 3 cells; SPC, stalk and polar cells; FSC/preFCs, follicle stem cells and pre-follicle cells; EFC, early follicle cells; MFC, mitotic follicle cells stage 1-5; PMFC, post-mitotic follicle cells stage 6; V7, vitellogenic main-body follicle cells (MBFCs) stage 7; V8, vitellogenic MBFCs stage 8; V9-10A, vitellogenic MBFCs stage 9-10A; C12, choriogenic MBFCs stage 12; C14, choriogenic MBFCs stage 14; TCLC, terminal corpus luteum cells; SC, stretch cells; OSM, ovarian sheath muscle; and O, oviduct. Un, unannotated.

We identified conserved germline and somatic cell populations in both testis and ovary, whose proportions and marker expression were highly conserved across all three strains. Using uniform manifold approximation and projection (UMAP) and graph-based clustering, we found 10 testis and 17 ovary cell types and annotated them using known marker genes (S4 Table). In the testis, we identified cells undergoing mitosis (Mi; GSC/early spermatogonia (ES) and late spermatogonia (LS)) and meiosis (Me; early spermatocytes (ESp), mid spermatocytes (MSp), late spermatocytes (LSp), maturing primary spermatocytes (MPSp), and post-meiotic spermatids (S)), along with three somatic populations (S; epithelial (EC), cyst (CC), and hub (HC) cells). In the ovary, we distinguished early germline cells in the germarium (G; germline stem cells and germarium region 1 and 2a (GSC/G1-2a), germarium region 2a and 2b (G2a-2b), and germarium region 2b and 3 cells (G2b-3)) from somatic cells in the germarium (GS; follicle stem cells (FSC/pre-FCs), early follicle cells (EFC), and stalk and polar cells (SPC)), and multiple somatic populations in the epithelium (EpS; main body follicle cells (V7, V8, V9-10A, C12, C14), oviduct (O), ovarian sheath muscle (OSM), stretch cells (SC), and terminal corpus luteum cells (TCLC)). These somatic cells play essential roles in stem cell maintenance, germ cell differentiation and survival, and sex-specific germline development [45–47]*.* UMAP visualization faithfully recapitulated the expected spatial arrangement of these cell types, despite the absence of spatial data (Fig 1A and 1B). We validated these annotations using developmental trajectory reconstruction, which recovered the expected progression of germline differentiation (S1 and S2 Figs).

**Fig 2:**
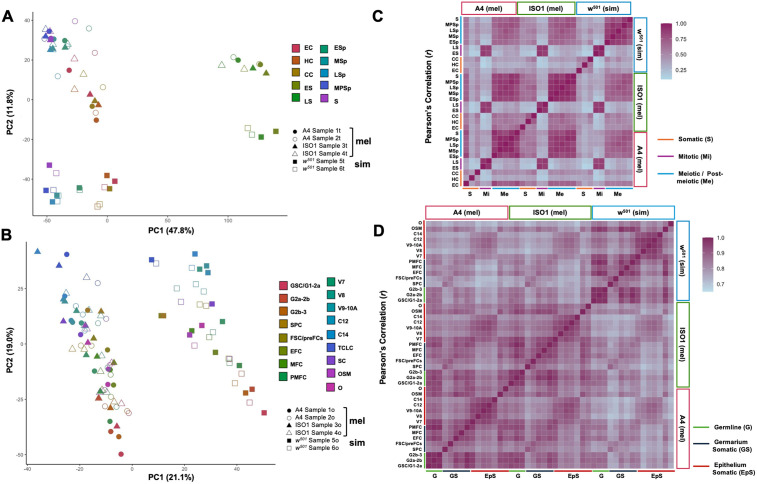
Intra- and interspecific expression variation and correlations across cell types in *Drosophila* gonads. Principal component analysis (PCA) of cell-type pseudo-bulks. **(A)** Testis. PC1 separates the mitotic cells from the meiotic and somatic cells. PC2 shows separation between species. **(B)** Ovary. PC1 shows the separation between species. PC2 shows cell progression along the oogenesis trajectory. Correlation matrices depicting Pearson’s correlation coefficients between the expression levels of genes found expressed in common between any two cell types across the three strains assayed in **(C)** testis and **(D)** ovary. A4 and ISO1, *D. melanogaster* strains; *w*^*501*^, *D. simulans* strain. In this correlation analysis, only cell types with at least 50 cells per strain and 350 cells across strains were included; as a result, the TCLC and SC ovarian cell types were excluded. Genes were required to be expressed in at least 1% of cells with a minimum average expression of 0.01. The order of cell types along both axes is identical, with broadly defined and precise cell types indicated on the *x-*axis and *y-*axis, respectively. Broad categories: somatic (S), mitotic (Mi), and meiotic (Me) categories for testis; and germline (G), germarium somatic (GS), and epithelium somatic (EpS) for ovary. Testis cell types: EC, epithelial cells; HC, hub cells; CC, cyst cells; ES, germline stem cells and early spermatogonia; LS, late spermatogonia; ESp, early spermatocytes; MSp, mid spermatocytes; LSp, late spermatocytes; MPSp, maturing primary spermatocytes; and S, spermatids. Ovary cell types: GSC/G1-2a, germline stem cells and germarium region 1 and 2a cells; G2a-2b, germarium region 2a and 2b cells; G2b-3, germarium region 2b and 3 cells; SPC, stalk and polar cells; FSC/preFCs, follicle stem cells and pre-follicle cells; EFC, early follicle cells; MFC, mitotic follicle cells stage 1-5; PMFC, post-mitotic follicle cells stage 6; V7, vitellogenic main-body follicle cells (MBFCs) stage 7; V8, vitellogenic MBFCs stage 8; V9-10A, vitellogenic MBFCs stage 9-10A; C12, choriogenic MBFCs stage 12; C14, choriogenic MBFCs stage 14; TCLC, terminal corpus luteum cells; SC, stretch cells; OSM, ovarian sheath muscle; and O, oviduct.

The testis and ovary cell types displayed strong signatures of evolutionary stability. The different strains exhibited similar overall contributions to each cell type (Fig 1C and 1D), with minimal differences in relative cell type composition (Figs 1E, 1F, and S3; S5 Table and S1 Text). This resulted in highly correlated cell proportions across strains (S6 Table). Furthermore, marker gene expression remained remarkably consistent across strains for all annotated cell types (S4 and S5 Figs). Principal component analysis revealed that expression profiles clustered by species and by broad cell type category—mitotic, meiotic/post-meiotic, and somatic in the testis, and along a developmental trajectory continuum in the ovary (Fig 2A and 2B). Biological replicates consistently clustered together, and the two strains of *D. melanogaster* always grouped more closely with each other than with *D. simulans*, reflecting their phylogenetic relatedness and confirming the validity of our sample preparation and sequencing pipeline. Together, these findings indicate the conserved developmental architecture of gametogenesis, enabling cell-type-level comparisons of gene expression evolution across species.

### Contrasting patterns of cell-type-specific expression divergence in the testis and ovary

The conserved cellular architecture of the gonads extended to the number of genes expressed per cell type, although we noted sharp within-tissue variation (S6 Fig). In the testis, late and maturing primary spermatocytes (LSp and MPSp, respectively), exhibited the highest median number of expressed genes, whereas mitotic cell types (ES and LS) had the fewest (Kruskal-Wallis chi-squared, *P <* 0.001 for each strain; S7 Table for post-hoc tests). In the ovary, germline stem cells and stage 1 and 2a cells in the germarium (GSC/G1-2a), and stage 12 choriogenic MBFCs (C12) expressed the highest median number of genes (Kruskal-Wallis chi-squared, *P <* 0.001 for each strain; S7 Table for post-hoc tests). These patterns remained stable across all three strains (S6 Fig).

Testis and ovary cell types displayed distinct trajectories of transcriptome differentiation. We quantified this differentiation at three levels: within strain, within species (A4 versus ISO1), and between species (*D. melanogaster* versus *D. simulans*). To do so, we calculated the average expression of each expressed gene for each gonadal cell type in each strain (S1 and S2 Data) and constructed gene expression correlation matrices for each tissue (Fig 2C and 2D). Within strains, median correlation values among broad cell-type categories differed significantly in both the testis and ovary, although this variation was larger in the testis (S7 Fig). This pattern indicates that transcriptome differentiation is more cell-type-specific in the testis than in the ovary (S8 Table). In the testis, median correlation values were significantly higher among mitotic cell types (Mi-Mi), among meiotic cell types (Me-Me), and between post-meiotic and meiotic categories (PMe-Me) (Kruskal-Wallis rank sum test; S7 Fig; S8 Table). In the ovary, the highest correlation values were found among germline cell types (G-G) (Kruskal-Wallis rank sum test; S7 Fig; S8 Table). When all pairwise cell type comparisons within each tissue were examined, within-strain median correlation values were significantly lower in the testis than in the ovary (two-way aligned rank transform (ART) ANOVA; S8 Fig; S9 Table).

Between strains, median expression correlations were also significantly higher among ovary cell types than among testis cell types, both within and between species (two-way ART ANOVA; S9 Fig; S10 Table). A closer examination by tissue revealed that the testis harbored the widest range of expression correlation values (min = 0.31, somatic cells, A4 versus *w*^*501*^; max = 0.94, meiotic cells, A4 versus ISO1). Among broad cell-type categories, meiotic cells exhibited the largest and the only significant difference between intra- and interspecific contrasts. In contrast, mitotic cell types–germline stem cells/ early spermatogonia (ES) and late spermatogonia (LS)–displayed similar median correlation values within and between species, suggesting a more conserved early transcriptional program. Somatic cell populations exhibited significantly lower correlation values than all other broad cell categories (two-way ART ANOVA; S10 Fig; S11 Table). By contrast, the ovary featured high intra- and interspecific correlation values (>0.75) across all broad cell-type categories, indicating more conserved expression patterns across both germline and somatic categories. Within each broad cell-type category, intraspecific median correlation values were significantly higher than interspecific ones (*P*_adj_ < 0.05). However, for any given pair of strains, median correlation values did not differ significantly among broad cell-type categories (two-way ART ANOVA; S11 Fig; S12 Table).

Together, these results show that expression correlations are highest within strain, lower within species, and lowest between species, but that this decline is highly tissue- and stage-specific. The ovary is more uniformly conserved, whereas the testis spans a much wider range of transcriptome differentiation, with meiotic cells showing the largest differences between intra- and interspecific comparisons.

### Testis cell types harbor more intra- and interspecific expression differences than ovary cell types

**Fig 3:**
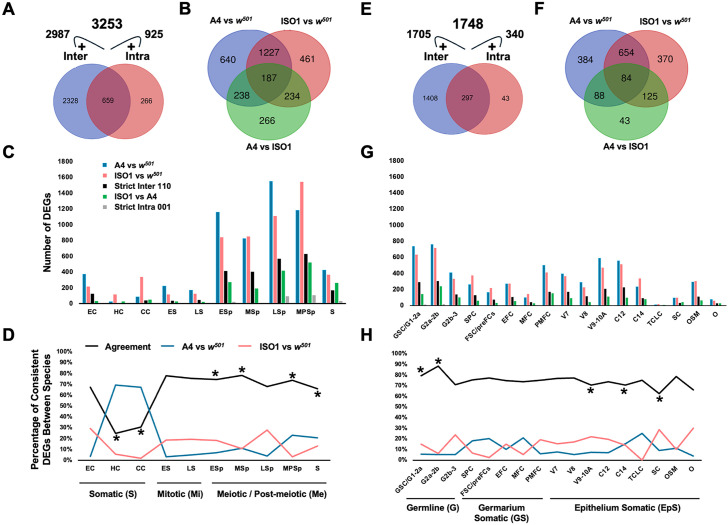
Magnitude of expression differentiation across testis and ovary cell types at two evolutionary timescales. Venn diagrams showing the number of differentially expressed genes (DEGs) unique to and shared between *D. melanogaster* and *D. simulans* in the testis: **(A)** at the inter- and intraspecific levels; and **(B)** across the three pairwise comparisons involving the *D. melanogaster* strains A4 and ISO1, and the *D. simulans* strain *w*^*501*^. **(C)** Patterns of differential expression across pairwise comparisons and testis cell types. Strict Inter (pattern 110): DEGs in both interspecific contrasts in the same cell type without being differentially expressed at the intraspecific level. Strict Intra (pattern 001): DEGs at the intraspecific level without being differentially expressed at the interspecific level. **(D)** Percentage of DEGs per testis cell type between the *D. melanogaster* strains A4 and ISO1, shown relative to the *D. simulans* strain *w*^*501*^. DEGs are categorized based on whether they show consistent differential expression in both interspecific comparisons (A4 vs. *w*^*501*^ and ISO1 vs. *w*^*501*^) or are unique to only one of the comparisons. **(E–H)** Equivalent plots for the ovary. Genes were required to be expressed in at least 1% of cells with a minimum average expression of 0.01. Asterisks, cell types showing the lowest and highest deviation relative to the random expectation for DEGs consistent across both interspecific contrasts (S14 Table). Testis cell types: EC, epithelial cells; HC, hub cells; CC, cyst cells; ES, germline stem cells and early spermatogonia; LS, late spermatogonia; ESp, early spermatocytes; MSp, mid spermatocytes; LSp, late spermatocytes; MPSp, maturing primary spermatocytes; and S, spermatids. Ovary cell types: GSC/G1-2a, germline stem cells and germarium region 1 and 2a cells; G2a-2b, germarium region 2a and 2b cells; G2b-3, germarium region 2b and 3 cells; SPC, stalk and polar cells; FSC/preFCs, follicle stem cells and pre-follicle cells; EFC, early follicle cells; MFC, mitotic follicle cells stage 1-5; PMFC, post-mitotic follicle cells stage 6; V7, vitellogenic main-body follicle cells (MBFCs) stage 7; V8, vitellogenic MBFCs stage 8; V9-10A, vitellogenic MBFCs stage 9-10A; C12, choriogenic MBFCs stage 12; C14, choriogenic MBFCs stage 14; TCLC, terminal corpus luteum cells; SC, stretch cells; OSM, ovarian sheath muscle; and O, oviduct.

To identify the genes driving patterns of expression correlation within and between species, we performed differential expression analyses, focusing on 11,481 one-to-one orthologous genes [48]. These analyses included 9,218 genes expressed in testis cell types and 8,533 genes expressed in ovary cell types, with 8,084 genes expressed in both (S3 and S4 Data). We defined differentially expressed genes (DEGs) using a 1% FDR and a log_2_ fold-change ≥ |1|, based on pairwise comparisons at both the intraspecific (ISO1 versus A4) and interspecific (ISO1 versus *w*^*501*^, and A4 versus *w*^*501*^) levels for each cell type and tissue. Consistent with previous work on the interspecific evolution of sex-biased expression [16,17,20,21], the testis showed greater expression divergence than the ovary (3,253 DEGs or 35.3% versus 1,748 DEGs or 20.5% of the expressed genes in each tissue, respectively; 2-sample test for equality of proportions with continuity correction or 2STEP, χ^2^ = 479.2, d.f. = 1, *P <* 2.2 × 10^−16^) (Fig 3A and 3E).

The two interspecific contrasts revealed overlapping but nonidentical DEG sets (Fig 3B and 3F), with a slightly higher percentage shared between contrasts in the testis compared to the ovary (47.3% or 1,414/2,987 versus 43.3% or 738/1,705, respectively; 2STEP, χ^2^ = 7.0, d.f. = 1, *P* = 8.05 × 10^−3^), a difference that remained when DEGs at the intraspecific level were omitted (52.7% or 1,227/2,328 versus 46.4% or 654/1,408, respectively; 2STEP, χ^2^ = 13.5, d.f. = 1, *P = 2*.4 × 10^−4^). For both tissues, and akin to our previous correlation analysis, the number of DEGs varied across cell types (Friedman rank sum test; testis: χ^2^ = 25.07, d.f. = 9, *P = 2*.9 × 10^−3^; ovary: χ^2^ = 45.39, d.f. = 16, *P* = 1.2 × 10^−4^), peaking in the late and maturing primary spermatocytes (LSp and MPSp, respectively) in the testis, and in the germline stem cells and germarium region 1 and 2a (GSC/G1-2a) and 2a-2b (G2a-2b) in the ovary (Figs 3C, 3G and S12). This pattern sharply contrasts with the mitotic and somatic cell types in the testis, and most somatic cell types in the ovary (*e.g.,* the follicle (FSC/pre-FCs and EFC) and oviduct (O) cell populations), which showed the lowest DEG counts between species. The number of DEGs per cell type was highly correlated across pairwise contrasts between strains (testis, minimum *r*^2^ = 0.78, *P* = 7.7 × 10^−4^; ovary, minimum *r*^2^ = 0.93, *P* = 6.4 × 10^−10^; S13 Table).

Expression differentiation across interspecific pairwise contrasts was also highly consistent at the cell type level. In the testis, 82.1% (1,007/1,227) of the DEGs were found in the same cell type across comparisons, with an average consistency of 63.7% across cell types, although varying significantly among them (test for equality of proportions, χ^2^ = 191.85, d.f. = 9, *P* = 2.2 × 10^−16^; cyst cells (CC) and mid spermatocytes (MSp), the lowest and highest deviation relative to the random expectation; S14 Table) (Fig 3D). The ovary showed comparatively higher consistency (92.5%, 605/654; 2STEP, χ^2^ = 37.07, d.f. = 1, *P* = 1.14 × 10^−9^), with an average of 74.5%, but also with significant differences across cell types (test for equality of proportions, χ^2^ = 89.87, d.f.=16, *P* = 2.6 × 10^−12^; stretch cells (SC), and germarium 2a-2b (G2a-2b), the lowest and highest deviation relative to the random expectation; S14 Table) (Fig 3H). Across both tissues, about 40% of consistent DEGs–those differentially expressed in the same cell type for both interspecific contrasts–were differentially expressed in only one cell type (S13 Fig). In the case of differential expression in more than one cell type, species directionality (*e.g*., upregulation in *D. simulans* relative to *D. melanogaster*) was preserved across all affected cell types for nearly all DEGs (1,004 and 604 DEGs in the testis and ovary, respectively). Furthermore, we detected a slight but significant tendency toward upregulation in *D. melanogaster* relative to *D. simulans* for DEGs found only in testis, compared to those found only in ovary (two-tailed Fisher’s exact test or FET, *P* = 0.033, odds ratio = 0.77). However, within each tissue, DEGs specific to one or shared between both tissues did not differ significantly in directional bias (FET, *P >* 0.05) (S14 Fig).

A sizable fraction of DEGs at the intraspecific level displayed interspecific differences (Fig 3A and 3E). This overlap was significantly greater in the ovary (87.4% or 297/340) than in the testis (71.2% or 659/925; 2STEP, χ^2^ = 34.08, d.f. = 1, *P* = 5.28 × 10^−9^). After excluding DEGs both at the intra and interspecific levels, we detected significantly more DEGs per cell type in the testis than in the ovary, and substantially fewer DEGs at the intraspecific level (2-way ART ANOVA, S15 Fig and S15 Table; and one subsequent one-sided permutation test for the counts of DEGs at the interspecific level between tissues due to acute overdispersion in testis, *P* = 0.048). However, the global ratio of the number of interspecific to intraspecific DEGs was significantly higher in the ovary than in testis (14.07 versus 3.79; 2STEP, χ^2^ = 63.64, d.f. = 1, *P* = 1.5 × 10^−15^). Because this ratio compares between-species divergence to standing within-species differentiation, lower values indicate that interspecific expression differences accumulate only modestly relative to intraspecific differences, which is suggestive of weaker functional constraints on the transcriptome program of a given tissue. Accordingly, the lower ratio observed in the testis denotes more relaxed functional constraints compared to the ovary. This ratio also varied substantially across cell types (S16 Fig). By explicitly incorporating intraspecific variation, our experimental design provides a baseline reference for interpreting interspecific divergence relative to standing expression variation, yielding a more accurate assessment of the selective constraints operating collectively on transcriptome programs of different tissues.

**Fig 4:**
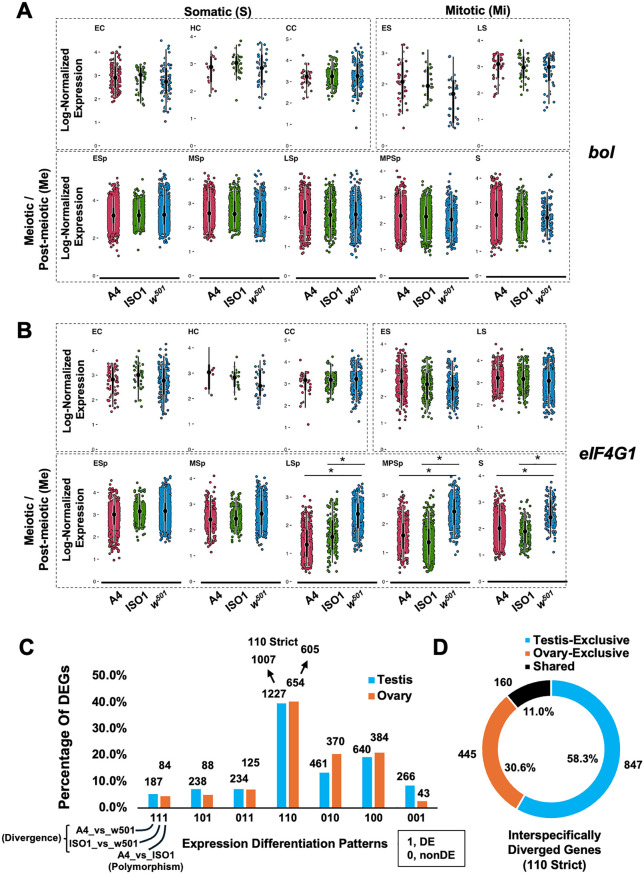
Expression differentiation patterns at the cell type level in *Drosophila.* **(A)** The gene *bol,* a non-DEG across testis cell types and strains, illustrates the stability in mRNA expression levels among strains, a pattern consistent with functional constraints. **(B)** The gene *eIF4G1* exemplifies a testis expressed gene that has diverged consistently at the interspecific level, but not at the intraspecific level. A4 and ISO1, *D. melanogaster* strains; *w*^*501*^, *D. simulans* strain. Asterisks denote statistically significant differences in mRNA levels at 1% FDR and a log_2_ fold-change ≥ |1|. **(C)** Histogram showing the percentage and the number (above bars) of DEGs in at least one cell type in at least one of the pairwise contrasts among the three strains. The combination of presence (1) or absence (0) of expression differences in each of the three pairwise contrasts results in seven possible patterns of expression differentiation. The categorization of each gene—including those showing no significant expression differences (000)—is provided in S3 and S4 Data. ‘110 Strict’ refers to the subset of DEGs entailing expression changes in at least one same cell type in both interspecific contrasts; most DEGs following a 110 pattern are part of this more stringent subset. **(D)** Donut chart showing the number and percentage of consistent DEGs at the interspecific level, exclusive to either testis or ovary, or shared between both tissues. Genes expressed in at least 1% of cells with a minimum average expression of 0.01 were considered. Testis cell types: EC, epithelial cells; HC, hub cells; CC, cyst cells; ES, germline stem cells and early spermatogonia; LS, late spermatogonia; ESp, early spermatocytes; MSp, mid spermatocytes; LSp, late spermatocytes; MPSp, maturing primary spermatocytes; and S, spermatids.

The increased resolution of snRNA-seq also uncovered fine-scale expression dynamics. For example, the gene *bol*, which encodes an RNA-binding protein essential for meiosis and spermatid differentiation [49,50], showed stable expression across strains and cell types (Fig 4A), consistent with the action of stabilizing selection. Conversely, the gene *eIF4G1*, which encodes a translation initiation factor involved in spermatogenesis [51], was downregulated in both *D. melanogaster* strains relative to *D. simulans* (Fig 4B), but only in late and maturing primary spermatocytes and spermatids (LSp, MPSp, and S, respectively). This pattern is compatible with the action of lineage-specific selection, although additional intraspecific expression data from both species and outgroup species are needed to substantiate this possibility. The patterns of expression for both genes, *bol* and *eIF4G1*, were independently validated by smFISH (S17 and S18 Figs; S5 and S6 Data), confirming the conserved expression of *bol* across cell types, and the downregulation of *eIF4G1* in a cell-type-specific manner. Equivalent patterns were observed for key genes during oogenesis such as *orb* and *ovo* [52,53] (S19 Fig). Some genes showed more complex patterns of gene expression evolution, diverging in one or both *D. melanogaster* strains relative to *D. simulans*, with or without intraspecific changes (Fig 4C).

In total, we documented 1,452 DEGs consistently diverged across both interspecific contrasts in at least one common cell type in the testis or ovary. Only 11.0% of these DEGs were shared across both tissues (Fig 4D). For this subset, the expression directionality between *D. melanogaster* and *D. simulans* was largely consistent across tissues. Only 21 genes displayed opposing patterns: 13 upregulated in *D. melanogaster* ovaries but downregulated in testes, and 8 showing the reversed pattern. Collectively, our findings suggest that the testis transcriptional program is more evolutionarily labile than that of the ovary, while providing enhanced resolution into how intra- and interspecific expression differences manifest at the level of individual cell types.

### Coexpression network modules diverge across species in a cell-type-specific manner

To determine whether gene expression divergence within individual cell types extended to broader coordinated changes at the network-level, we performed a coexpression network analysis (hdWGCNA [54]). Our analysis revealed cell-type-specific modules of coexpressed genes encompassing 3,700 genes in the testis, and 4,730 genes in the ovary (S16 Table; S7 and S8 Data). Roughly half of the constituent genes (1,642 in the testis and 2,655 in the ovary) were assigned to modules in more than half of the cell types, including known gametogenesis genes such as *polo* [55], *CycE* [56], and *mei-P26* [57]. Conversely, a smaller set of genes (396 genes in the testis; 320 in the ovary) was uniquely assigned to modules in 20% or fewer cell types, highlighting potential cell-type-specific roles (S20A Fig).

To assess the functionality of these coexpression networks, we identified hub genes, defined as the top 10 genes within each module ranked by their intramodular connectivity, kME [58]. These included known regulators of spermatogenesis and oogenesis, including *dj*, which is involved in mitochondrial differentiation within the flagellum during sperm individualization [59], *S-Lap8*, a protease-encoding gene crucial for spermatogenesis [60], and the previously mentioned *orb* and *ovo*, which are required for egg chamber formation and polarity establishment, and the activation of multiple maternally expressed genes during oocyte development, respectively [52,53]. As expected, hub genes were enriched for GO terms tied to key aspects of germline development and gamete function, including spermatid development (GO:0007286), sperm motility (GO:0097722), and cytoplasmic translation (GO:0002181) (S17 Table).

Next, we examined whether the coexpression modules showed species-specific expression patterns by conducting a differential module eigengene (DME) analysis [54]. We found that about half of the coexpression modules exhibited interspecific divergence across testis and ovary cell types, with at least one divergent module per cell type (S20B Fig; S16 Table). In the testis, divergent modules were enriched during the later stages of spermatogenesis, consistent with our findings that species-specific transcriptional differences are most pronounced in the terminally differentiating meiotic cell types. A similar but nonsignificant trend was observed in the ovary, where terminal cell types—defined as the main body follicle cells—were compared against all other cell types (terminal cell types versus the rest, 2STEP; testis, χ^2^ = 4.62, d.f.=1, *P* = 3.2 × 10^−2^; ovary, χ^2^ = 1.92, d.f. = 1^,^ *P* = 0.17).

We further assessed the association between DEGs and the number of divergent coexpression modules across cell types. Under a random distribution of DEGs across modules, we expect that cell types with more divergent modules would also contain proportionally more DEGs, resulting in a positive correlation between these metrics. Alternatively, if DEGs are concentrated within a limited subset of divergent modules, no such proportional relationship would be observed, indicating uneven modular contributions to expression network differentiation. Our results indicate that divergence in the testis is broadly distributed across modules, whereas in the ovary, divergence is concentrated in a restricted subset of coexpression modules (Spearman’s correlation coefficient; testis, ρ = 0.782, *P* = 7.5 × 10^−3^; ovary, ρ = −0.084, *P* = 0.766*;* S21 Fig). Notably, in the testis, 13 DEGs within interspecifically divergent modules are also found in an ancient spermatocyte protein interaction network conserved from *Drosophila* to humans [61]. None of the 13 genes was part of our list of top 1% hub genes, and only the knock-down of one of them resulted in impairment of male fertility [61]. These properties suggest that even genes part of this metazoan male germ cell network can accommodate transcriptional divergence at short phylogenetic distances with no detrimental consequences.

**Fig 5:**
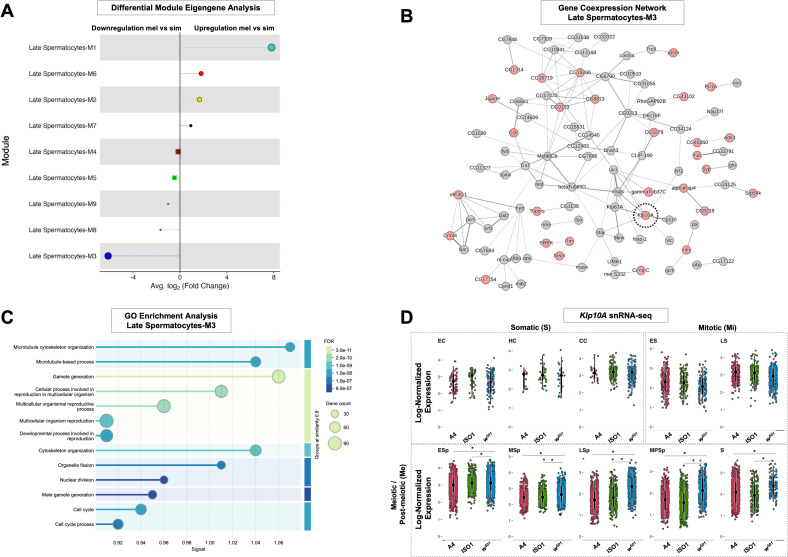
Divergent coexpression network in late spermatocytes between *D. melanogaster* and *D. simulans.* **(A)** Differential module eigengene (DME) analysis showing the average log_2_ fold change in coexpression module activity between *D. melanogaster* and *D. simulans* in late spermatocytes (minimum module size = 100 genes). Module M3 is upregulated in *D. simulans*, while Module M1 is upregulated in *D. melanogaster*. **(B)** Gene coexpression network structure of the top 160 genes in Module M3 in late spermatocytes (medium confidence threshold = 0.4; FDR = 5%). Red nodes represent genes that are significantly downregulated in *D. melanogaster* relative to *D. simulans* according to the DEG analysis. Network edges denote the level of confidence, and disconnected nodes are omitted. *Klp10A*, a DEG part of this subnetwork, is highlighted with a dashed circle. **(C)** Gene Ontology enrichment results (Biological Process category) for the top 500 genes in Module M3. **(D)** Single-nucleus RNA-seq expression patterns of *Klp10A*, a representative gene from Module 3 (panel B), across testis cell types. The cell type of interest, late spermatocytes (LSp), is highlighted. Asterisks denote statistically significant differences in mRNA levels at 1% FDR and a log_2_ fold-change ≥ |1|. Testis cell types: EC, epithelial cells; HC, hub cells; CC, cyst cells; ES, germline stem cells and early spermatogonia; LS, late spermatogonia; ESp, early spermatocytes; MSp, mid spermatocytes; LSp, late spermatocytes; MPSp, maturing primary spermatocytes; and S, spermatids.

The results of the DME and DEG analyses reinforced one another, providing a coherent picture of cell-type-specific expression divergence between *D. melanogaster* and *D. simulans*. For example, in late spermatocytes (LSp), the DME analysis revealed several divergent coexpression modules, with Module M3 and Module M1 showing a marked upregulation in *D. simulans* and in *D. melanogaster,* respectively (Fig 5A). Examination of DEG distributions within these modules showed that 29 constituent genes were upregulated in *D. simulans* in Module M3 (Fig 5B, nodes in red), while none were downregulated, aligning with the overall pattern of network-level divergence. GO enrichment analysis of this module highlighted processes such as microtubule cytoskeleton organization (GO:0000226) and nuclear division (GO:0000280) (Fig 5C; S18 Table). In contrast, module M1 contained 27 genes upregulated in *D. melanogaster* and none downregulated (S22A Fig, nodes in green). This module was enriched for processes such as mitochondrial transport (GO:0006839), male gamete generation (GO:0048232), and spermatogenesis (GO:0007283) (S18 Table; S22B Fig). Representative genes from both coexpression modules, such as the microtubule-associated gene *Klp10A* [62] and the somatic sex differentiation gene *janB* [63], further illustrate the cell-type-specific nature of these species-specific expression shifts (Figs 5D and S22C). As independently validated by smFISH, *Klp10A* exhibits upregulation in late spermatocytes (LSp) of *D. simulans*, while its expression remains conserved in nonmeiotic cell types such as late spermatogonia (LS) (S23 Fig; S9 Data). This aligns with the canonical role of *Klp10A* as a centriole-length regulator in mitotic cells of the testis [62], while suggesting that it may perform an additional meiotic function in *D. simulans*. Such species-specific regulatory roles at the level of individual genes and entire subnetworks are suggestive of functional specialization between *D. melanogaster* and *D. simulans*.

Another example of cell-type-specific network-level divergence was observed in the germarium region 2a-2b cells (G2a-2b) of the ovary. The DME analysis uncovered key coexpression modules underlying early oogenesis divergence, with two modules, M1 and M2, displaying distinct coexpression patterns between species (S24A–S24C Fig). Module M1 was upregulated in *D. simulans* and was enriched for pathways related to germ cell development (GO:0007281) and oogenesis (GO:0048477) (S19 Table; S24D Fig). A notable example is the gene *rhi* (S24F Fig), which encodes a germline-restricted heterochromatin protein essential for fertility and transposable element (TE) silencing [64], and proposed to be implicated in female hybrid sterility between *D. simulans* and *D. melanogaster* [65,66]. Conversely, Module M2 was overexpressed in *D. melanogaster* and predominantly enriched for cell-cycle (GO:0007049) and related GO terms. One of the hub genes in this module, *mei-W68*, mediates double-strand break formation during meiosis [67] (S19 Table; S24E and S24G Fig). Notably, DEGs within both divergent modules exhibited typical connectivity relative to non-DEGs, indicating that divergence is not confined to peripheral genes, particularly for M1 where DEGs were in fact significantly more connected than non-DEGs (Kruskal-Wallis rank sum test; M1: χ² = 17.94, d.f. = 1, *P* = *2*.28 × 10^−5^; M2: χ² = 1.11, d.f. = 1, *P* = 0.291)*.* These findings demonstrate species-specific divergence in germline differentiation and cell-cycle regulation during oogenesis, supported by both DME and DEG analyses, which together reveal cell-type-specific transcriptional changes between *D. melanogaster* and *D. simulans*.

### Functional constraint across gonadal cell types is stage-dependent in the testis but uniform in the ovary

**Fig 6:**
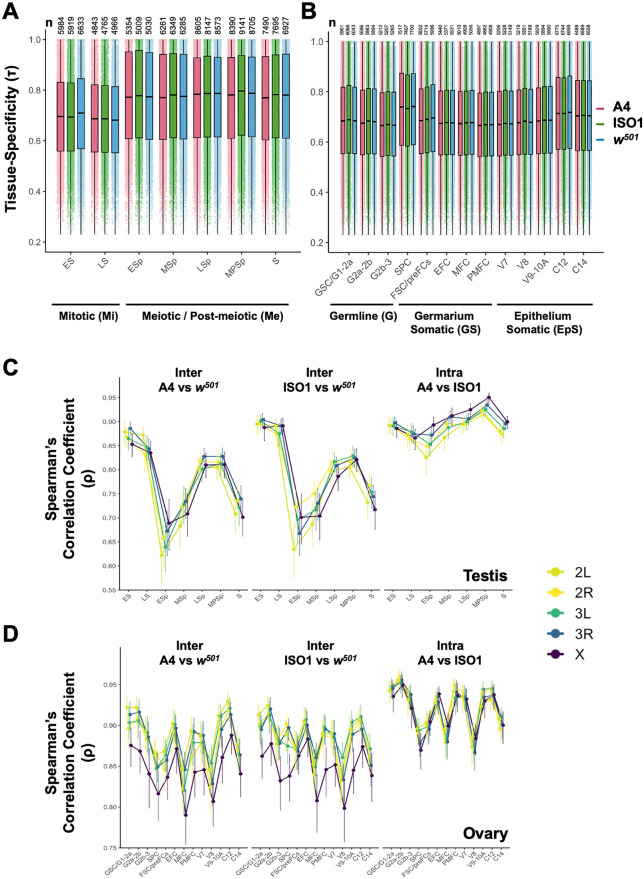
Expression breadth and chromosome expression correlation across testis and ovary cell types. **(A, B)** Tau index (τ) distributions, a measure of expression specificity, across testis and ovary, respectively, for all expressed genes in specific cell types. Higher τ values indicate more restricted expression across tissues, *i.e.,* a proxy for lower pleiotropy. Numbers above each boxplot denote the number of expressed genes per cell type. Only cell types directly involved in gametogenesis and with at least 100 cells per strain and 350 across all strains were included. Genes expressed in at least 1% of each cell type and with a minimum average expression of 0.01 are considered. Boxes represent the interquartile range around the median (black horizontal line) and whiskers extend to 1.5 times the interquartile range. Each point represents the tau value of a particular gene, and values are grouped by strain and cell type. A4 and ISO1, *D. melanogaster* strains; *w*^*501*^, *D. simulans* strain. **(C, D)** Spearman’s correlation coefficient (ρ) of gene expression between strains across cell types in the testis and ovary, respectively, calculated separately for each major chromosome arm (*2*L, *2*R, *3*L, *3*R, and *X*). Error bars represent the 95% confidence interval across replicates. Expression values above the 95th percentile within each cell type were omitted to reduce the impact of outliers in the expression correlation analyses.

The differences in expression divergence across cell types—particularly the greater evolutionary divergence of meiotic cells in the testis—suggest that functional constraints vary across gametogenesis. To test this hypothesis, we examined whether genes expressed in different cell types exhibit varying degrees of pleiotropy, given that pleiotropy is linked to the magnitude of functional constraint on a gene [68–70]. We used the tau index [71], calculated from FlyAtlas2 tissue-level expression data [72], to estimate expression breadth—a proxy for pleiotropy and functional constraint [68,69,73]— across genes expressed in different gonadal cell types. In the testis, tau values differed significantly across cell types (Kruskal-Wallis test, *P* < 1 × 10^−100^ for all three strains), with a stepwise increase from mitotic to meiotic stages (Fig 6A; S20 Table). In contrast, tau values in the ovary were lower overall and exhibited less variation across cell types. Among ovarian cell types, three somatic populations—stalk and polar cells (SPC), and choriogenic stage 12 (C12) and stage 14 (C14) MBFCs, the latter two involved in the late stages of oogenesis—showed the highest tau values (Fig 6B; S20 Table). Overall, the meiotic cells of the testis displayed the highest tau values of all gonadal cell types, suggesting weaker functional constraint, which is hypothesized to be associated with a more permissive chromatin environment found in these cell types [74–76].

We next examined expression breadth at the cell-type level within each tissue. This allowed us to ask whether tissue-level patterns were also observed at finer resolution. We repurposed the tau index to quantify cell-type specificity in our single-nucleus dataset for each tissue and strain. In the ovary, cell-type-level tau values recapitulated the patterns observed in the tissue-level analysis (S25 Fig). In the testis, however, the pattern was reversed: genes expressed in early spermatocytes, which showed high tau values in the tissue-level analysis, now displayed the lowest tau values when expression breadth was assessed across testis cell types. This indicates that although these genes are testis-biased at the tissue-level, they are broadly expressed across cell types within the testis, consistent with stronger functional constraint within that tissue (S21 Table; S25 Fig). These results suggest that patterns of functional constraint depend on the biological scale at which expression breadth is assessed and underscore the importance of accounting for within-tissue variation.

Given that expression breadth is also correlated with phylogenetic gene age [77], we hypothesized that young genes (age categories B-E, *i.e.,* genes that arose during the radiation of the genus *Drosophila*) would be enriched in late spermatogenesis stages, while older genes (*i.e.,* age category A, predating the radiation of the genus *Drosophila*) would be overrepresented in mitotic cell types. Using a precisely curated gene age categorization in flies [78]*,* we found substantial support for these hypotheses (S26 Fig). In contrast, the ovary revealed a contrasting pattern with only the germarium region 2b-3 cells (G2b-3) showing enrichment for ancient genes, while the three aforementioned somatic cell types with higher tau exhibited a significantly elevated presence of younger genes from multiple age categories (S27 Fig).

The rate of protein evolution (ω, the ratio of nonsynonymous to synonymous substitutions) provides an additional proxy for the strength of functional constraint and is known to covary with expression divergence, particularly in sex-biased genes [16], which are predominantly expressed in germline tissues [12]. To calculate ω, we used divergence and polymorphism data from *D. simulans* and two *D. melanogaster* populations—one from the species’ ancestral range and one from a derived, post-colonization population—to account for potential effects of differing demographic histories [79]. In the testis, genes expressed in meiotic cell types exhibited significantly higher ω values than those expressed in mitotic cell types (S22 Table; S28A Fig), which was consistent across all strains. In the ovary, ω was relatively uniform across cell types, with the exception of stage-12 choriogenic MBFCs (C12), which exhibited slightly elevated ω compared to most other cell types for all three strains (S23 Table; S28B Fig).

To disentangle the contributions of adaptive and nonadaptive mechanisms to these differences, we partitioned ω into its adaptive (ωₐ) and nonadaptive (ω_na_) components [80–82]. In the testis, meiotic cells had elevated ωₐ values, particularly in the early and mid-spermatocytes (ESp and MSp, respectively), suggesting a stronger role for positive selection (S22 Table; S28C Fig). In contrast, ω_na_ peaked in late spermatocytes (LSp), indicating relaxed purifying selection during the later stages of spermatogenesis (S22 Table; S28E Fig). In the ovary, both ωₐ and ω_na_ were more uniform across cell types (S23 Table; S28D and S28F Fig). In both tissues, *X*-linked genes consistently displayed higher ωₐ values than autosomal genes, suggesting a faster-*X* effect at the sequence level, driven by positive selection (S29A and S29B Fig). Since the median ωₐ for autosomal genes was significantly lower relative to ω_na_ for all strains and tissues (paired, two-sided Wilcoxon signed-rank test, V = 0, *P*_adj_ < 0.02 for all comparisons), the sequence evolution of autosomal genes is impacted more by nonadaptive than by adaptive mechanisms (S29C and S29D Fig).

Collectively, patterns of functional constraint differ starkly between the testis and ovary. The testis exhibits stage-dependent shifts in constraint across cell types, with early spermatocytes simultaneously showing the lowest tau (i.e., broader within-testis expression) and elevated rates of adaptive evolution (ωₐ), suggesting a dual role in hosting core functions while enabling innovation. Genes expressed in later meiotic stages, in contrast, showed higher nonadaptive rates of sequence evolution (ω_na_), suggesting more relaxed functional constraints. In the ovary, both expression breadth and evolutionary rates were more uniform across cell types, with only a few somatic populations (SPC, C12, C14) showing modest deviations and increased expression of younger genes. Genes on the *X*-chromosome showed consistently higher adaptive rates, indicating that chromosomal location is associated with differences in protein evolution of genes expressed in the gonadal tissues. Together, these results highlight the distinct evolutionary regimes operating in male and female gonads.

### Genomic distribution of interspecifically divergent genes is biased in the female germline

The faster evolution of *X*-linked protein-coding genes expressed in the *Drosophila* gonads at the sequence-level raises the question of whether expression divergence also shows a faster-*X* effect in the testis and ovary. The hemizygosity of the *X* chromosome has been hypothesized to result in greater divergence at the coding and expression levels compared to autosomes [83]. Although the evidence is mixed [13,19,84–86], faster-*X* effects have been primarily reported for male-biased genes in expression [19,85], many of which are preferentially expressed in the testis. We investigated how divergent genes between species at the cell type level were distributed across the genome (S30A Fig). Consistent DEGs between species exclusive to the ovary, or shared across both tissues, deviated from the random expectation (chi-squared goodness of fit test, χ^2^ = 51.35, d.f. = 4, *P*_adj_ *=* 1.88×10^−10^, and χ^2^ = 26.74, d.f. = 4, *P*_adj_ *=* 2.24 × 10^−5^, respectively). Post-hoc tests revealed that both categories of divergent genes were enriched on the *X* chromosome, with ovary-exclusive DEGs also significantly depleted from several autosomes (S24 Table; S30B Fig). In stark contrast, testis-exclusive DEGs showed a distribution not significantly different from the random expectation (chi-squared goodness of fit test, χ^2^ = 5.96, d.f. = 4, *P*_adj_ *=* 0.202) (S30B Fig).

To test the faster-*X* effect more broadly by including both DEGs and non-DEGs, we analyzed the correlation between expression levels per chromosome and cell type [19,84,86]. Interspecific comparisons confirmed greater *X-*linked expression divergence (*i.e.,* lower Spearman’s correlation coefficients) in the ovary but not in the testis, mirroring the DEG distribution results (Fig 6C and 6D; S25 Table). At the intraspecific level, we found no differences across chromosomes, and all patterns were recapitulated at the pseudo-bulk level (S31A Fig).

Both adaptive and nonadaptive mechanisms can generate a faster-*X* effect [87]. To distinguish between these possibilities, we assessed the strength of purifying selection on gene expression by analyzing the correlation of gene expression between the testis and ovary in each strain across chromosomes at the bulk-level [19]. Across strains and species, the *X* chromosome consistently exhibited higher testis-ovary correlation values than most autosomes (S31B Fig). These findings suggest that the pronounced expression divergence on the *X* is unlikely to result from relaxed functional constraints, instead pointing to positive selection as a more plausible driver of the observed faster-*X* effect.

Overall, our findings challenge traditional views from previous *Drosophila* studies, as we do not find evidence for a faster-*X* effect associated with gene expression divergence in testis [19,85,86]. The discrepancy between an observed faster-*X* effect at the coding-level and the absence of this effect at the expression level in testis has also been found in mammals [39,88]. The paradox may be explained by the unique biology of the testis, with permissive chromatin enabling widespread baseline expression, which is less sensitive to regulatory changes [75], combined with potential regulatory constraints associated with meiotic sex chromosome inactivation (MSCI) or a shutdown of dosage compensation in the meiotic cells [23,89]. The enrichment of DEGs exclusive to the ovary and those shared between tissues on the *X* chromosome points to a substantial, persistent role for positive selection acting on *X-*linked genes in females—the sex in which the *X* chromosome resides two-thirds of the time in any given generation—, presumably through dominant or partially dominant mutations [90,91].

## Discussion

Our analysis of 106,328 testis and ovary nuclei from three *Drosophila* strains resolves cell type–specific patterns of transcriptional divergence at short phylogenetic distances. Traditional bulk transcriptomic studies in *Drosophila* lacked cell type resolution [13,16,17,20,92,93], limiting inferences about fine-scale expression differences. We identified numerous key genes involved in spermatogenesis (*Jupiter*, *janB,* and *eIF4G1* [63,94,95]) and oogenesis (*ovo*, *rhi*, and *mei-W68* [52,64,67]) that display interspecific expression differences in a cell-type-specific manner, extending similar observations from mammalian gonads [9] and other tissues [10,41,96–99]. Our results also reveal new insights into gene functionality. For example, *ovo*, known for its central role in oocyte development [52], shows species-specific expression in main-body follicle cells (MBFCs), suggesting a somatic role consistent with isoform-specific activity [100].

Transcriptome evolution during spermatogenesis is especially dynamic, with divergence concentrated in meiotic and post-meiotic germ cells. These stages harbor more DEGs and divergent coexpression networks than earlier stages, providing an increased opportunity for regulatory divergence. Our findings align with prior studies on sectioned testis from *D. melanogaster* and *D. simulans* [101] and mammalian snRNA-seq that identified late spermatogenesis as a hotspot of expression divergence [9]. In mammals, this has been linked to narrower spatiotemporal expression profiles and a greater prevalence of evolutionarily young genes [9], facilitated in part by a more permissive chromatin state [74–76]. These factors may allow both neutral and adaptive expression changes to accumulate—neutral changes due to weak or no purifying selection [102], and adaptive changes in response to different selective pressures, most notably sperm competition [76,103].

We found evidence for reduced functional constraints as spermatogenesis progresses in *Drosophila*. Phylogenetically younger genes with narrower expression breadth and faster rates of sequence evolution are more prevalently expressed at later stages, which also display lower ratios of interspecific to intraspecific expression changes. Consistent with these patterns, our network-level analyses identified mitochondrial transport and microtubule-based processes—both essential for germ cell development [104,105]—as major targets of transcriptome divergence in late spermatogenesis. This divergence may reflect species-specific adaptations shaped by sexual selection, particularly sperm competition [106]. Notably, sperm length—a key factor in sperm competition—depends on the microtubule cytoskeleton for elongation [105], a trait that differs markedly between *D. melanogaster* and *D. simulans* [107]. Together, these findings support a model of accelerated transcriptome evolution in late spermatogenesis, driven by a combination of adaptive and nonadaptive evolutionary mechanisms.

In the ovary, although overall divergence is more limited than in the testis (605 DEGs in the ovary versus 1,007 DEGs in the testis), cell-type-specific differences are still prominent. Genes expressed in the ovary tend to show more uniform evolutionary properties across cell types—older age, broader expression, and slower sequence evolution—reflecting stronger pleiotropic properties and therefore stronger functional and evolutionary constraints. However, the global ratio of interspecific to intraspecific expression changes is significantly higher in the ovary than in the testis, perhaps reflecting a higher dependency of the ovary transcriptional program on transcription factor activity [75], and suggesting a more moderate role for nonadaptive mechanisms driving interspecific transcriptome divergence in the ovary. Among the most divergent cell types are terminally differentiating main-body follicle cells (MBFCs), which provide structural and regulatory support to the oocyte [108], and early-stage germline cells. For example, in germarium region 2a-2b germline cells (G2a-2b), which contain a mix of meiotic and nurse cells that have exited meiosis [109], we identified divergent coexpression modules associated with TE suppression, cell-cycle processes, and meiotic progression. This mirrors observations in mammals, where species-specific divergence in early oogenesis is linked to variation in cell-cycle timing [110], and is consistent with recent findings suggesting that the female meiotic germ cells may be especially evolutionarily dynamic [111]. The presence of meiotic gene expression in early germline cells of the germarium may help explain why divergence is detectable at these early stages [112]. These observations suggest that fine-scale regulation of mitotic-to-meiotic transitions—or meiosis itself—may be an important target of expression divergence in the ovary, as in the testis, with implications for oocyte quality and reproductive success. Furthermore, we find that *X*-linked genes show higher expression divergence and faster evolution at the protein-coding level than autosomal genes regardless of cell type, which seems to be primarily driven by adaptive mechanisms. Together, our findings suggest that the ovary evolves more slowly than the testis but is far from being transcriptionally static, undergoing cell-type-specific divergence even in core biological processes.

Overall, our findings reveal a striking asymmetry in the evolution of the male and female germlines. The testis exhibits stage-dependent expression divergence across cell types, with mitotic spermatogonia remaining highly conserved and meiotic spermatocytes emerging as a hotspot of divergence. In contrast, the ovary shows more constrained expression evolution along its developmental trajectory, with *X*-linked genes exhibiting elevated expression divergence that is consistent with a primary role of adaptive mechanisms. Across both tissues, gene age and pleiotropy are associated with the degree of cell-type-specific divergence, underscoring the influence of both adaptive and nonadaptive forces. These results support a more granular model of transcriptome evolution than previously recognized—one that reflects distinct biological demands and varying exposure to selection mechanisms [113,114]. Finally, we provide a publicly accessible resource for further exploring interspecific expression divergence during gametogenesis (https://ranzlab-server.bio.uci.edu/Drosophila_snRNA-seq_Gene_Expression_Browser_Ovary/ and https://ranzlab-server.bio.uci.edu/Drosophila_snRNA-seq_Gene_Expression_Browser_Testis/).

## Materials and methods

### Fly husbandry

Two strains of *D. melanogaster* (A4 and the reference strain ISO1; Bloomington *Drosophila* stock center ids #3852 [115] and 2057 [116], respectively), and the strain *w*^*501*^ of *D. simulans* (*Drosophila* species stock center #id: 14021-0251.195 [117]) were used. Flies were reared on a standard dextrose-cornmeal-yeast medium at room temperature (~25 °C) under continuous light. All manipulations of flies were performed under CO_2_ anesthesia.

### Gonadal tissue dissection

Testis dissections were performed on 1–2-day-old virgin males and ovary dissections on 3-day-old virgin females using fine-tipped forceps in cold Schneider’s Drosophila Medium (ThermoFisher, #21720024). The selected age prevented the overrepresentation of mature sperm that characterizes older males [7] and ensured that females were fully fertile and receptive [118]. Dissected tissues were immediately transferred to 200 μL of Schneider’s Medium in nuclease-free 1.5 mL Eppendorf tubes, pooled (10–40 tissues per tube), and kept on ice for less than 2 h. Separate petri dishes were used for dissections of different strains and tissues to avoid cross-contamination and ensure sample integrity. After dissections were completed, samples were flash-frozen by submerging the tubes in liquid nitrogen and immediately stored at −80 °C for long-term preservation. To maintain RNA integrity, samples were thawed only prior to nuclei isolation. Two biological replicates for each dissected tissue and strain were generated. In total, we dissected 1,200 pairs of testes (200 pairs per biological replicate) and 240 pairs of ovaries (40 pairs per biological replicate).

### Nuclei isolation

Nuclei isolation from *Drosophila* ovary and testis was performed using a two-step dissociation process. Initially, tissues were incubated in HBSS Buffer containing 2.5 mg/mL collagenase (Invitrogen, #17018-029) at 37°C for 15 min. The tissues were then homogenized in EZ Lysis buffer (Sigma-Aldrich, #NUC101-1KT) and incubated on ice for 10 min, followed by filtration through a 70 μm filter. The filtered homogenate was centrifuged at 750 *g* for 5 min at 4°C and resuspended in 1mL of lysis buffer. After a second centrifugation, the nuclei were incubated in Nuclei Wash and Resuspension buffer (1xPBS, 1% BSA, 0.5U/μL RNase inhibitor) for 5 min. To remove debris, a debris removal solution (Miltenyi Biotec, #130-109-398) was added to the nuclei suspension, and the mixture was centrifuged at 3,000 *g* for 10 min at 4°C. The purified nuclei were fixed and permeabilized using the Nuclei Fixation Kit (Parse Biosciences) and cryopreserved in DMSO until library preparation.

### Preparation of snRNA-seq library and sequencing

Libraries were prepared using the EVERCODE WT V2 kit (Parse Biosciences) and quantified with the Qubit dsDNA HS assay kit (Invitrogen, #Q32851). The average fragment length of each sub-library was measured using the D5000 HS kit (Agilent, #5067-5592, #5067-5593). Finally, the eight sub-libraries were sequenced on the Illumina Novaseq 6000 S4 platform using paired-end sequencing at the UCI Genomics Research and Technology Hub, with an estimated sequencing depth of 50,000 read pairs/nuclei.

### snRNA-seq read alignment

Raw sequencing data were assessed using FastQC (version 0.11.9) [119] to evaluate read quality, sequence composition, and potential sequencing artifacts. Read alignment was performed against the respective genome assembly of each *Drosophila* strain: ISO1 [120], A4 [32], and *w*^*501*^ [121]. To facilitate comparative genomic analysis, we employed Liftoff (version 1.6.3) [122] to annotate the A4 genome by mapping gene annotations from the ISO1 reference genome to the A4 assembly using default parameters. We filtered each annotation in the GTF file to retain protein-coding genes only using 10X Genomics’ CellRanger mkgtf (version 8.0.1) [123] prior to creating the references using the ‘mkref’ mode of Parse Biosciences’ split-pipe pipeline (version 1.1.2) [124]. Reads from each of the eight sub-libraries were demultiplexed by strain and aligned independently to the three respective references using the ‘all’ mode of split-pipe. The alignments were configured to incorporate each sample’s designated wells, in addition to specifying the V2 chemistry of the library preparation protocol and a post_min_map_frac of 0.01. Lastly, we combined the resulting alignments from the eight sub-libraries for each sample using the ‘comb’ mode of split-pipe to generate the final count matrices.

### Quality control, normalization, and integration

We considered previously delineated orthologous calls between *D. melanogaster* to *D. simulans* [48] and replaced the *D. simulans* gene names with those from *D. melanogaster* while retaining the genes without any one-to-one mapping. We combined the data from our two biological replicates to create one Seurat object for each tissue and strain, applying a stringent quality control criteria with the following thresholds: min.features = 200, min.cells = 3, and a minimum nCount_RNA of 300 for testis and 500 for ovary (version 5.1.0) [125]. We removed nuclei with mitochondrial reads above 5% and limited the dataset to nuclei with a maximum of 7,500 expressed genes and 100,000 counts to remove outliers, including nuclei whose gene counts are inflated by doublets/multiplets or other technical artifacts. These thresholds were implemented while inspecting violin and feature scatter plots to assess the distribution of the metrics across samples and using the Fly Cell Atlas (FCA) snRNA-seq dataset as a benchmark reference generated from comparable tissues [7]. Each of these datasets was then log-normalized (NormalizeData) and scaled (ScaleData) before obtaining a tissue- and strain-specific UMAP using Seurat’s built-in functions (RunPCA, FindNeighbors, FindClusters, RunUMAP).

DoubletFinder (version 2.0.4) [126] was employed to minimize the potential effect of doublets by using the recommended iterative parameter sweeping approach to identify the optimal pK (a neighborhood-size parameter) value for each Seurat object. We assumed a default doublet formation rate of 3% (https://support.parsebiosciences.com/hc/en-us/articles/360053107311-What-is-the-expected-doublet-rate) and used a homotypic doublet proportion estimation based on initial clustering results. Nuclei classified as doublets were removed from downstream analyses.

The post-filtered Seurat objects were merged into a single object for each tissue, and normalization (NormalizeData) and variable feature identification (FindVariableFeatures) were performed for each layer independently. The cross-strain layers were integrated using Harmony (version 1.2.1) [44], which performed the best among other tools tested (LIGER [127], RPCA -https://satijalab.org/seurat/articles/integration_rpca.html-, CCA [128], scVI [129]), and is also recommended for closely related species, carrying out species mixing while preserving biological heterogeneity [130].

### Clustering and cell type annotation

The integrated layers were subsequently joined for each tissue, and an ElbowPlot was used to determine the number of PCs for the FindNeighbors command for both testis (dims = 1:15) and ovary (dims = 1:20). We used clustree (version 0.5.1) [131] for interrogating the clusters at increasing resolution before running FindClusters on the testis (resolution = 1.2) and ovary (resolution = 1) Seurat objects (S32 Fig). We generated a combined UMAP for each tissue using the harmony reductions and visualized the plots using scpubR (version 2.0.2) [132]. Cell type annotations were inferred using known marker genes specifically expressed in each cell type (S4 Table; S4 and S5 Figs), as documented in recently published single-cell and single-nucleus RNA-seq studies of the gonadal tissue of *Drosophila* (testis [7,22–24]; ovary [7,25,26,28]).

Several control analyses were implemented to confirm expected species clustering and validate our cell type annotation. First, we performed a pseudo-bulk analysis and generated an MDS plot for each cell type of each tissue, confirming that biological replicates clustered by strain. Second, the proportions of different cell types were compared across strains using pairwise Pearson’s correlations (S1 Text). Lastly, a trajectory analysis using Monocle3 (version 1.3.5) [133–135] was performed to demonstrate that the annotated cell types were arranged in the expected developmental sequence of gametogenesis for each tissue (S1 and S2 Figs).

### Principal component analysis of cell-type pseudo-bulks

To examine expression differences within each tissue, we aggregated raw gene expression counts by cell type and sample to generate pseudo-bulk profiles. We combined these profiles into a single matrix with corresponding cell type and sample metadata, removed genes with zero counts across all pseudo-bulks, and applied variance-stabilizing transformation using the rlog function in DESeq2. We then performed principal component analysis on the transformed matrix and visualized the top two components to assess variation between cell types and strains (Fig 2A and 2B).

### Correlation of gene expression

To assess the degree of gene expression correlation for the same cell type across *Drosophila* strains, and among different cell types within strains, we identified orthologous genes present in all three strains (ISO1, A4, and *w*^*501*^). We extracted the average gene expression levels of all 1-to-1 orthologous genes between *D. melanogaster* and *D. simulans* and generated a correlation matrix using Pearson correlation coefficients, which was visualized using a heatmap.

### Differential gene expression analysis

We performed pairwise differential gene expression analyses for each interspecific (A4 versus *w*^*501*^; ISO1 versus *w*^*501*^) and intraspecific (ISO1 versus A4) comparison for testis and ovary using MAST (version 1.28.0) [136]. Differential expression was evaluated for each gene and cell type by comparing expression levels between two given strains under the following criteria: |log2 fold change| ≥ 1 and FDR ≤ 1%. We further corrected for multiple testing across the three comparisons using the Benjamini-Hochberg method (FDR ≤ 1%) and classified genes as differentially expressed (DE) or nondifferentially expressed (nDE). Genes that did not meet a minimum expression threshold of min.pct = 0.01 in a given cell type for both strains being compared were excluded from the analysis, thereby mitigating possible artifacts associated with low expression.

We adopted a systematic approach to characterize expression differentiation patterns across the three pairwise comparisons in the testis and ovary using custom in-house Python scripts. We implemented an encoding system where each gene received a three-digit binary code representing its differential expression status across these comparisons (A4 versus *w*^*501*^; ISO1 versus *w*^*501*^; ISO1 versus A4) for each tissue. Each digit can either be ‘0′ for an nDE gene or ‘1′ for a DE gene in at least one of the cell types considered (10 for the testis and 17 for ovary, respectively). Thus, interspecifically diverged genes had a code of ‘110′, which was compared between testis and ovary to investigate tissue-specific gene expression evolution. A similar 10- or 17-digit trinary code (0, no difference; +1, overexpression in strain 1 versus strain 2; and −1, underexpression in strain 1 versus strain 2) was applied for summarizing patterns of differential expression across cell types within each comparison for the testis and ovary, respectively.

### Weighted gene co-expression network analysis (hdWGCNA)

To identify modules of significantly co-expressed genes, we performed weighted gene co-expression network analysis (WGCNA) using the hdWGCNA framework (version 0.3.01) [54]. The analysis was carried out independently for each cell type, incorporating cells from all three strains, focusing exclusively on genes shared across strains and expressed in at least 5% of cells. Metacells were constructed by grouping cells with nearest-neighbor (k = 25) aggregation using the Harmony-reduced space. Metacells shared by more than 15 cells were excluded, groups with fewer than 50 cells were discarded, and the resulting metacell expression matrix was log normalized. The “signed” network type was used for optimizing the soft-thresholding power and maximize the scale-free topology fit. A topological overlap matrix (TOM) was computed for each cell type, and hierarchical clustering with dynamic tree cutting (deepSplit = 4, minimum module size = 25) was applied to identify co-expression modules. Module eigengenes (MEs) were calculated for each module and hub genes within each module were identified based on eigengene connectivity (kME) by ranking genes by kME. Modules assigned to the “gray” category, indicating unassigned genes, were excluded from downstream analyses.

To assess interspecies differences in module activity, we performed differential module eigengene (DME) analysis using hdWGCNA’s ‘FindDMEs’ function. Module eigengene expression profiles were compared between *D. melanogaster* and *D. simulans*, applying min.pct = 0.01 to exclude lowly expressed genes. We identified significantly divergent modules for each cell type using the Wilcoxon rank-sum test and compared the constituent genes of these divergent modules with the differentially expressed genes to examine congruency between network-level and gene-level patterns of expression divergence.

### Functional enrichment analysis

We analyzed gene ontology (GO) term enrichment among genes present in co-expressed gene modules for each cell type and tissue using clusterProfiler (version 4.0) [137] with genome-wide annotations from org.Dm.eg.db (version 3.18.0) [138]. Enrichment results were restricted to the top 500 genes per module and we applied a 5% FDR [139]. To identify shared and cell-type-specific functional enrichments, we used the compareCluster function in clusterProfiler to compare GO term enrichment across modules.

### Single-molecule RNA fluorescence in situ hybridization

We followed the smFISH protocol as described previously for the *Drosophila* testis [140]. Briefly, 10 pairs of testes from 1-day-old males were dissected on ice in 1× PBS and collected in 1.5 mL Eppendorf tubes. After centrifugation, PBS was removed, and tissues were fixed in 4% formaldehyde (in PBS) for 30 min on a nutator. Samples were then washed twice in PBS, transferred to 70% ethanol, and incubated overnight at 4 °C. Ethanol was removed and samples were briefly incubated in wash buffer A (2 × SSC, 10% formamide). Hybridization was carried out overnight at 37 °C in hybridization buffer containing 2 × SSC, 10% dextran sulfate, 1 mg/mL *E. coli* tRNA, 2 mM vanadyl ribonucleoside complex, 0.5% BSA, and 10% formamide, with 1 µL of 12.5 μM Stellaris probe solution targeting each focal gene (*bol, eIF4G1,* and *Klp10A*). On Day 3, without removing the hybridization solution, 1 mL of wash buffer A was added into the tube and incubated for 30 min at 37 °C. The tube was then centrifuged at 750 *g* for 5 mins, followed by a final wash in wash buffer B for 30 min at 37 °C. Samples were mounted in VECTASHIELD with DAPI (Vector Labs) and imaged immediately. Imaging was performed on a Nikon Ti2-U Inverted Fluorescence Microscope. All solutions used for RNA FISH were RNase-free. Stellaris RNA FISH probes were synthesized by LGC Biosearch Technologies. Probes were designed following blastn homology searches [141] to avoid nucleotide sequence differences between the open reading frames of *D. melanogaster* and *D. simulans* orthologs for the focal genes*.*

Quantification of smFISH signal was performed in Fiji/ImageJ [142]. The smFISH channel from each image was processed by background subtraction. Putative transcript puncta were identified using the *Find Maxima* function with a fixed prominence threshold of 40 for all images analyzed. Quantification was performed within rectangular regions of interest (ROIs) scaled to correspond to the same anatomical area across images. Guided by tissue morphology, these regions generally correspond to GSC/early spermatogonia (ES), late spermatogonia (LS), late spermatocytes (LSp), or spermatids (S). The number of detected puncta within each ROI was recorded and used for downstream comparisons of smFISH signal across strains and developmental stages.

### Genomic features

We examined several functional and genomic features in the context of expression divergence between *D. melanogaster* and *D. simulans.* Chromosomal locations and sequence coordinates were retrieved from FlyBase [143]. For expression breadth, we obtained gene expression values across 31 tissues and body parts from adult individuals of both sexes as in FlyAtlas2 [72], and calculated the tissue-specific index tau (τ) [71]. The tau index ranges from 0 to 1 with lower and higher values indicating broader and narrower expression, respectively.

We retrieved the phylogenetic age of genes inferred under a parsimonious framework based on syntenic alignments among 20 fly species, including the species *Scaptodrosophila lebanonensis* and *Bactrocera dorsalis*, which are outgroups to the genus *Drosophila* [78]. The five age classes considered correspond to different tree branches in the original publication: class A corresponds to genes originated in branches −1 and −2, *i.e.,* those present before the *Drosophila* radiation; class B corresponds to branch 0, *i.e.,* those that arose prior to the split between the *Drosophila* and *Sophophora* subgenera; class C corresponds to branches 1 and 2, *i.e.,* to those genes originated in the lineages to the *D. willistoni* and *D. pseudoobscura* species groups; class D corresponds to branch 3, *i.e.,* to those genes originated during the radiation of the *D. melanogaster* species group; and class E corresponds to branches 4–6, *i.e.,* to those originated during the evolution of the *D. melanogaster* species subgroup, which includes *D. melanogaster* and *D. simulans*.

Gene sequence evolution was analyzed through a modified version of the McDonald and Kreitman test, the extended McDonald and Kreitman test [144], which controls for low frequency polymorphisms and differentiates neutral from weakly deleterious variants, thus minimizing the problem of underestimating α, *i.e.,* the proportion of substitutions fixed by positive selection (α) [82]. Synonymous and nonsynonymous nucleotide substitutions were obtained from the comparison of sequences involving *D. simulans,* 197 lines from a *D. melanogaster* African population (Zambia), and 205 lines from a North American *D. melanogaster* population (Raleigh, North Carolina) [145], using the iMKT R package [144]. From the iMKT site, we also downloaded the allele frequency spectrum. To estimate the number of neutral segregating sites in the nonsynonymous category, we applied a minimum 5% frequency threshold to remove slightly deleterious mutations [144]. Alpha was calculated considering the corrected estimates, which allowed us to calculate the rate of protein evolution (ω), the rate of adaptive evolution (ωa), and the rate of nonadaptive evolution (ωna) [80,81].

### Statistical analyses

Two-sample test for equality of proportions with continuity correction, two-tailed Fisher’s exact test, chi-squared goodness-of-fit, chi-squared test of independence, analysis of residuals, Kruskal-Wallis rank sum, post-hoc tests, two-way ART (aligned rank transform) ANOVA, regression analysis, linear modeling, and Benjamini-Hochberg correction for multiple testing were conducted using built-in functions in R (version 4.3.2) [146,147].

### Declaration of generative AI and AI-assisted technologies in the writing process

During the preparation of this manuscript, the authors used ZotGPT (GPT-4o, GPT-5, and Claude Sonnet 4.5) to improve readability and language. After using this tool, the authors reviewed and edited the content as needed and take full responsibility for the content of the publication.

## Supporting information

S1 Fig Pseudotime projected onto the UMAPs of the testis and ovary datasets. UMAPs of the **(A)** testis and **(B)** ovary, colored by pseudotime values as inferred using Monocle 3 [135]. In both tissues, pseudotime recapitulates the expected developmental progression across gametogenesis, with early germline or progenitor populations occupying lower pseudotime values and more differentiated cell states distributed along later pseudotime regions of the embedding. The overall distribution of pseudotime is consistent with the annotated developmental hierarchy of testis and ovary cell types.
(TIFF)

S2 Fig Pseudotime progression across annotated cell types of the testis and ovary inferred from developmental trajectories. **(A, B)** Boxplots show the distribution of pseudotime values for cells annotated as specific cell types, ordered vertically to reflect the inferred developmental progression for **(A)** testis and **(B)** ovary, based on Monocle3 analysis. In both tissues, pseudotime recapitulates known differentiation hierarchies, with early germline or stem cell populations occupying lower pseudotime values and differentiated somatic or germline cells positioned later.
(TIFF)

S3 Fig Regression analysis of cell type composition across pairwise comparisons between strains. **(A, B)** Best-fit regression lines for the cell type proportions detected with snRNA-seq between A4 and two other strains (ISO1, blue; and *w*^501^, orange) are shown with their corresponding equations, coefficients of determination, and statistical significance of the latter for the testis and ovary, respectively. ISO1 and A4, *D. melanogaster*; *w*^501^, *D. simulans.* Cell clusters that were unannotated or did not fulfill the requirement of at least 50 nuclei per strain and 350 across the three strains (*i.e.,* terminal corpus luteum cells (TCLC) and stretch cells (SC) in the ovary), were not considered in the analysis. The diameter of each datapoint denotes the coefficient of variation (CV) for the proportion of a particular cell type across the three strains considered. The 95% confidence intervals are shaded.
(TIFF)

S4 Fig Conserved marker gene expression in testis. Dot plots showing the average gene expression of marker genes in different testis cell types: **(A)** mitotic cells – spermatogonia (ES and LS); **(B)** somatic cells (HC, CC, and EC); **(C)** meiotic cells – spermatocytes (ESp, MSp, LSp, and MPSp); and **(D)** spermatids (S). The size of each dot represents the proportion of cells in which the gene is expressed. Gene expression values are normalized and scaled, with darker solid colors indicating overexpression relative to the average and lighter gray shades indicating underexpression. EC, epithelial cells; HC, hub cells; CC, cyst cells; ES, germline stem cells and early spermatogonia; LS, late spermatogonia; ESp, early spermatocytes; MSp, mid spermatocytes; LSp, late spermatocytes; MPSp, maturing primary spermatocytes; and S, spermatids.
(TIFF)

S5 Fig Conserved marker gene expression in ovary. Dot plots showing the average gene expression of marker genes in different ovary cell types: **(A)** germline cells (G1-2a, G2a-2b and G2b-3); **(B)** germarium somatic cells (SPC, FSC/preFCs, EFC, MFC and PMFC); **(C)** epithelium somatic cells – main body follicle cells (V7, V8, V9-10A, C12 and C14); and **(D)** ovarian sheath muscle (OSM) and oviduct (O). The size of each dot represents the proportion of cells in which the gene is expressed. Gene expression values are normalized and scaled, with darker solid colors indicating overexpression and lighter gray shades indicating underexpression. Cell types: GSC/G1-2a, germline stem cells and germarium region 1 and 2a cells; G2a-2b, germarium region 2a and 2b cells; G2b-3, germarium region 2b and 3 cells; SPC, stalk and polar cells; FSC/preFCs, follicle stem cells and pre-follicle cells; EFC, early follicle cells; MFC, mitotic follicle cells stage 1–5; PMFC, post-mitotic follicle cells stage 6; V7, vitellogenic main-body follicle cells (MBFCs) stage 7; V8, vitellogenic MBFCs stage 8; V9-10A, vitellogenic MBFCs stage 9-10A; C12, choriogenic MBFCs stage 12; C14, choriogenic MBFCs stage 14; TCLC, terminal corpus luteum cells; SC, stretch cells; OSM, ovarian sheath muscle; and O, oviduct.
(TIFF)

S6 Fig Number of genes expressed across cell types and strains in the testis and ovary. **(A, B)** Geyser plots showing the number of expressed genes detected across the cells within particular cell types for the three strains assayed (A4 and ISO1, *D. melanogaster: w*^*501*^, *D. simulans*) in the testis and ovary, respectively. Broad categories of cell types for each tissue are indicated on top. Within each plot, the median, the 66% (thick line) and the 95% (thin line) of the data are shown. Testis cell types: EC, epithelial cells; HC, hub cells; CC, cyst cells; ES, germline stem cells and early spermatogonia; LS, late spermatogonia; ESp, early spermatocytes; MSp, mid spermatocytes; LSp, late spermatocytes; MPSp, maturing primary spermatocytes; and S, spermatids. Ovary cell types: GSC/G1-2a, germline stem cells and germarium region 1 and 2a cells; G2a-2b, germarium region 2a and 2b cells; G2b-3, germarium region 2b and 3 cells; SPC, stalk and polar cells; FSC/preFCs, follicle stem cells and pre-follicle cells; EFC, early follicle cells; MFC, mitotic follicle cells stage 1–5; PMFC, post-mitotic follicle cells stage 6; V7, vitellogenic main-body follicle cells (MBFCs) stage 7; V8, vitellogenic MBFCs stage 8; V9-10A, vitellogenic MBFCs stage 9-10A; C12, choriogenic MBFCs stage 12; C14, choriogenic MBFCs stage 14; TCLC, terminal corpus luteum cells; SC, stretch cells; OSM, ovarian sheath muscle; and O, oviduct.
(TIFF)

S7 Fig Within-strain expression correlations differ significantly among broad cell-type categories in both the testis and ovary. Violin (foreground) and box (background) plots showing the distribution of within-strain expression correlations across contrasts between broad cell-type categories in the testis and ovary. Boxes represent the interquartile range (IQR) around the median (horizontal line), and whiskers extend to 1.5 times the IQR. Median correlation values, top; number of correlation values considered (n), bottom. The outcomes of the post hoc tests can be found in S8 Table. Testis broad cell-type categories: Me, meiotic; Mi, mitotic; PMe, post-meiotic; and S, somatic. Ovary broad cell-type categories: EpS, epithelium somatic; G, germline; and GS, germarium somatic.
(TIFF)

S8 Fig Within-strain expression correlations between pairs of cell types are lower in the testis than in the ovary. Violin (foreground) and box (background) plots showing the distribution of within-strain expression correlation values between pairs of cell types in the testis and ovary across the three strains assayed. Boxes represent the interquartile range (IQR) around the median (horizontal line), and whiskers extend to 1.5 times the IQR. Median correlation values, top; number of correlation values considered (n), bottom. The outcomes of the post hoc tests can be found in S9 Table. Strains: *D. melanogaster* (A4, ISO1); *D. simulans* (*w*^*501*^).
(TIFF)

S9 Fig Between-strain expression correlations across equivalent cell types are higher within species than between species and are higher in the ovary than in the testis. Violin (foreground) and box (background) plots showing the distribution of between-strain expression correlation values for equivalent cell types in the testis and ovary across the three pairwise strain contrasts. Boxes represent the interquartile range (IQR) around the median (horizontal line), and whiskers extend to 1.5 times the IQR. Median correlation values, top; number of correlation values considered (n), bottom. The outcomes of the post hoc tests can be found in S10 Table. Strains: *D. melanogaster* (A4, ISO1); *D. simulans* (*w*^*501*^).
(TIFF)

S10 Fig Between-strain expression correlations across equivalent broad cell-type categories in the testis are largely similar within and between species and are lowest in somatic cells. Violin (foreground) and box (background) plots showing the distribution of between-strain expression correlation values across three pairwise strain contrasts and four broad cell-type categories in the testis. Boxes represent the interquartile range (IQR) around the median (horizontal line), and whiskers extend to 1.5 times the IQR. Median correlation values, top; number of correlation values considered (n), bottom. The outcomes of the post hoc tests can be found in S11 Table. Testis broad cell-type categories: Me, meiotic; Mi, mitotic; PMe, post-meiotic; and S, somatic. Strains: *D. melanogaster* (A4, ISO1); *D. simulans* (*w*^*501*^). T, testis.
(TIFF)

S11 Fig Between-strain expression correlations are similar across equivalent broad cell-type categories in the ovary, although higher within than between species. Violin (foreground) and box (background) plots showing the distribution of between-strain expression correlation values across three pairwise strain contrasts and three broad cell-type categories in the ovary. Boxes represent the interquartile range (IQR) around the median (horizontal line), and whiskers extend to 1.5 times the IQR. Median correlation values, top; number of correlation values considered (n), bottom. The outcomes of the post hoc tests can be found in S12 Table. Ovary broad cell-type categories: EpS, epithelium somatic; G, germline; and GS, germarium somatic. Strains: *D. melanogaster* (A4, ISO1); *D. simulans* (*w*^*501*^). O, ovary.
(TIFF)

S12 Fig Shared and unique patterns of differential expression across cell types in *Drosophila* testis and ovary. UpSet plots depicting differential expression patterns at different phylogenetic scales for testis and ovary comparisons. Testis: **(A)** *D. melanogaster* ISO1 versus A4 (intraspecific); **(B)** *D. melanogaster* ISO1 versus *D. simulans w*^*501*^ (interspecific); and **(C)** *D. melanogaster* A4 versus *D. simulans w*^*501*^ (interspecific). Ovary: **(D)** *D. melanogaster* ISO1 versus A4; **(E)** *D. melanogaster* ISO1 versus *D. simulans w*^*501*^; and **(F)** *D. melanogaster* A4 versus *D. simulans w*^*501*^. A bar graph is plotted alongside each UpSet plot to depict the number of differentially expressed genes detected per cell type. Broad cell type categories are indicated: testis – somatic (S), mitotic (Mi), and meiotic (Me); and ovary – germline (G), germarium somatic (GS), and epithelium somatic (EpS).
(TIFF)

S13 Fig Scope of interspecific differentiation in gene expression across cell types of gonadal tissues. Fraction of differentially expressed genes (DEGs) between species, categorized by the number of different cell types in which both *D. melanogaster* strains (ISO1, A4) differ from *D. simulans w*^*501*^.
(TIFF)

S14 Fig The directionality of interspecific expression for different patterns of differential expression and tissues. Up- and downregulation refer to significantly higher and lower expression levels in *D. melanogaster* versus *D. simulans,* respectively. Differentially expressed genes (DEGs) exclusively in testis, or ovary, do not tend to be more upregulated in *D. melanogaster* compared to *D. simulans* relative to DEGs in both tissues, as determined by Fisher’s Exact Tests (*p*-values shown for each contrast). When focusing on DEGs exclusively in one tissue, there is some evidence of a higher tendency of upregulation in *D. melanogaster* relative to *D. simulans* for those detected only in testis versus those detected only in ovary.
(TIFF)

S15 Fig Distributions of the number of differentially expressed genes across cell types in the testis and ovary for different strain comparisons. Violin (foreground) and box (background) plots showing the distribution of differentially expressed genes (DEGs) at the inter and intraspecific levels. At the intraspecific level, genes differentially expressed at the cell type level between the two strains of *D. melanogaster* (ISO1, A4) were considered. At the interspecific level, only genes consistently found differentially expressed in the same cell type of the two strains of *D. melanogaster* relative to one of *D. simulans* (*w*^*501*^) were considered. Boxes represent the interquartile range (IQR) around the median (horizontal line), and whiskers extend to 1.5 times the IQR. Median DEG counts, top; number of cell types considered (n), bottom. The outcomes of the post hoc tests can be found in S15 Table.
(TIFF)

S16 Fig Ratio of interspecific to intraspecific differentiation in gene expression across testis and ovary cell types. **(A, B)** Testis and ovary, respectively. Ratio of the average number of DEGs in interspecific comparisons (*D. melanogaster* A4 versus *D. simulans w*^*501*^, *D. melanogaster* ISO1 versus *D. simulans w*^*501*^) to the number of DEGs between the two strains of *D. melanogaster* (A4 versus ISO1). Testis cell types: EC, epithelial cells; HC, hub cells; CC, cyst cells; ES, germline stem cells and early spermatogonia; LS, late spermatogonia; ESp, early spermatocytes; MSp, mid spermatocytes; LSp, late spermatocytes; MPSp, maturing primary spermatocytes; and S, spermatids. Ovary cell types: GSC/G1-2a, germline stem cells and germarium region 1 and 2a cells; G2a-2b, germarium region 2a and 2b cells; G2b-3, germarium region 2b and 3 cells; SPC, stalk and polar cells; FSC/preFCs, follicle stem cells and pre-follicle cells; EFC, early follicle cells; MFC, mitotic follicle cells stage 1–5; PMFC, post-mitotic follicle cells stage 6; V7, vitellogenic main-body follicle cells (MBFCs) stage 7; V8, vitellogenic MBFCs stage 8; V9-10A, vitellogenic MBFCs stage 9-10A; C12, choriogenic MBFCs stage 12; C14, choriogenic MBFCs stage 14; TCLC, terminal corpus luteum cells; SC, stretch cells; OSM, ovarian sheath muscle; and O, oviduct.
(TIFF)

S17 Fig smFISH validation of *bol* expression levels across cell types and strains. **(A)** Representative smFISH images of *bol* mRNA expression in the testis from the strains A4, ISO1, and *w*^*501*^. Pink and red dashed boxes mark the standardized regions of interest (ROIs) used for quantification in the GSC/Early Spermatogonia (ES) and Spermatids (S), respectively. Nuclei were marked with DAPI. Scale bars, 100 µm. **(B)** smFISH puncta counts quantified within fixed-size ROIs in ES and S. Violin plots show the distribution across images, embedded boxplots show the median and interquartile range, and points represent individual values. Sample sizes are shown above each strain. Kruskal–Wallis tests were performed separately for ES and S. *bol* signal intensity did not differ significantly across strains in either cell type. The data underlying this figure are provided in S5 Data.
(TIFF)

S18 Fig smFISH validation of the expression levels in different cell types of the gene *eIF4G1* across strains. **(A)** Representative smFISH images of *eIF4G1* mRNA expression in the testis from the strains A4, ISO1, and *w*^*501*^. Pink and yellow dashed boxes mark the standardized regions of interest (ROIs) used for quantification in Late Spermatogonia (LS) and Late Spermatocytes (LSp), respectively. Nuclei were marked with DAPI. Scale bars, 100 µm. **(B)** smFISH puncta counts quantified within fixed-size ROIs in LS and LSp. Violin plots show the distribution across images, embedded boxplots show the median and interquartile range, and points represent individual values. Sample sizes are shown above each strain. Kruskal–Wallis tests were performed separately for LS and LSp, followed by pairwise two-sided Mann–Whitney tests with Benjamini–Hochberg correction for multiple tests in the case of LSp. *eIF4G1* signal intensity was similar across strains in LS, whereas in LSp it was significantly elevated in *w*^*501*^ relative to A4 and ISO1*.* The data underlying this figure are provided in S6 Data.
(TIFF)

S19 Fig Examples of expression differentiation among three *Drosophila* strains at the cell type level in ovary. **(A)** The gene *orb* shows no differential expression across cell types or strains, suggesting functional constraints operating on mRNA abundance. **(B)** The gene *ovo* shows consistent upregulation in the two *D. melanogaster* strains compared to *D. simulans* in two cell types (V8, V9-10A), with no intraspecific difference. Cell types: GSC/G1-2a, germline stem cells and germarium region 1 and 2a cells; G2a-2b, germarium region 2a and 2b cells; G2b-3, germarium region 2b and 3 cells; SPC, stalk and polar cells; FSC/preFCs, follicle stem cells and pre-follicle cells; EFC, early follicle cells; MFC, mitotic follicle cells stage 1–5; PMFC, post-mitotic follicle cells stage 6; V7, vitellogenic main-body follicle cells (MBFCs) stage 7; V8, vitellogenic MBFCs stage 8; V9-10A, vitellogenic MBFCs stage 9-10A; C12, choriogenic MBFCs stage 12; C14, choriogenic MBFCs stage 14; TCLC, terminal corpus luteum cells; SC, stretch cells; OSM, ovarian sheath muscle; and O, oviduct.
(TIFF)

S20 Fig Salient features of coexpression modules in *Drosophila* gonads. **(A)** Number of genes present across different coexpression modules. **(B)** Number of conserved and divergent coexpression modules between *D. melanogaster* and *D. simulans* per cell type. Coexpression modules were delineated with hdWGCNA [54].
(TIFF)

S21 Fig The number of DEGs scales up with the number of divergent coexpression modules in the testis but not in ovary. **(A, B)** Testis and ovary. Divergent coexpression modules between *D. melanogaster* and *D. simulans* were delineated with hdWGCNA [54], while differential expression on a gene basis was determined independently at a 1% FDR and a log_2_ fold-change ≥ |1| for both interspecific contrasts, i.e., A4 versus *w*^*501*^ and ISO1 versus *w*^*501*^. The Spearman’s rho and its statistical significance are provided for each linear relationship.
(TIFF)

S22 Fig *D. melanogaster*-biased coexpression module and gene expression divergence in late spermatocytes. **(A)** Gene coexpression network structure of the top 160 genes in Module M1 in late spermatocytes (LSp; medium confidence threshold = 0.4; FDR = 5%). Green nodes represent genes that are significantly upregulated in *D. melanogaster* relative to *D. simulans* according to our differential expression analysis. Network edges denote the level of confidence and disconnected nodes are omitted. **(B)** Gene Ontology enrichment results (Biological Process category) for the top 500 genes in Module M1. **(C)** Single-nucleus RNA-seq expression patterns for one of the genes in this coexpression module, *janB,* across the meiotic testis cell types (also circled in (A)). This gene is upregulated in *D. melanogaster* relative to *D. simulans* in several cell types, including late spermatocytes (LSp). Asterisks denote statistically significant differences in mRNA levels at 1% FDR and a log_2_ fold-change ≥ |1|. Testis cell types (c): ESp, early spermatocytes; MSp, mid spermatocytes; LSp, late spermatocytes; MPSp, maturing primary spermatocytes; S, spermatids.
(TIFF)

S23 Fig smFISH validation of the expression levels in different cell types of the gene *Klp10A* across strains. **(A)** Representative smFISH images of *Klp10A* mRNA expression in the testis from the strains A4, ISO1, and *w*^*501*^. Pink and red dashed boxes mark the standardized regions of interest (ROIs) used for quantification in Late Spermatogonia (LS) and Late Spermatocytes (LSp), respectively. Nuclei were marked with DAPI. Scale bars, 100 µm. **(B)** smFISH puncta counts quantified within fixed-size ROIs in LS and LSp. Violin plots show the distribution across images, embedded boxplots show the median and interquartile range, and points represent individual values. Sample sizes are shown above each strain. Kruskal–Wallis tests were performed separately for LS and LSp, followed by pairwise two-sided Mann–Whitney tests with Benjamini–Hochberg correction for LSp. *Klp10A* signal intensity was similar across strains in LS, whereas in LSp it was significantly elevated in *w*^*501*^ relative to A4 and ISO1. The data underlying this figure are provided in S9 Data.
(TIFF)

S24 Fig Gene coexpression network expression divergence between *D. melanogaster* and *D. simulans* in germarium region 2a–2b germline cells of the ovary. **(A)** Differential module eigengene (DME) analysis showing log_2_(fold change) of coexpression module activity between species. Significant interspecific differences affect Modules M1 and M2 (≥100 genes). **(B, C)** Coexpression network of the top 160 genes in Modules M1 and M2 (medium confidence = 0.4, 5%FDR). Red nodes indicate downregulation in *D. melanogaster* (M1), while green nodes indicate upregulation in *D. melanogaster* (M2). Network edges indicate the level of confidence, and disconnected nodes are hidden. **(D, E)** GO enrichment analysis (Biological Process) for the top 500 genes in Modules M1 and M2. **(F, G)** Expression levels of the genes *rhi* (M1) and *mei-W68* (M2) across early germline and follicular ovary cell types; the genes are also indicated with blue and turquoise circles in (B) and (C), respectively. The cell type of interest, Germline-Germarium 2a–2b cells, is highlighted. Cell types: GSC/G1-2a, germline stem cells and germarium region 1 and 2a cells; G2a-2b, germarium region 2a and 2b cells; G2b-3, germarium region 2b and 3 cells; EFC, early follicle cells; and MFC, mitotic follicle cells stage 1–5.
(TIFF)

S25 Fig Intra-tissue expression specificity across the testis and ovary cell types. **(A, B)** Cell-type tau distributions (τ), a measure of intra-tissue expression specificity, across testis and ovary, respectively, for all expressed genes by cell type. Higher τ values indicate more restricted expression across cell types within a given tissue. Only cell types directly involved in gametogenesis and with at least 100 cells per strain and 350 across all strains were included. Genes expressed in at least 1% of each cell type and with a minimum average expression of 0.01 are considered. Boxes represent the interquartile range around the median (black horizontal line) and whiskers extend to 1.5 times the interquartile range. Each point represents the cell-type tau value of a particular gene, and values are grouped by strain and cell type. A4 and ISO1, *D. melanogaster* strains; *w*^*501*^, *D. simulans* strain.
(TIFF)

S26 Fig Phylogenetic age distribution of genes expressed across testis cell types. Bar plots show the observed and expected (darker and lighter bars, respectively) percentages of expressed genes in each phylogenetic age class **(A–E)** across testis cell types for each strain (ISO1 and A4, *D. melanogaster*; *w*^*501*^, *D. simulans*). Class A includes the oldest genes and Class E the youngest. Vertical arrows denote statistically significant deviations (↑, enrichment; ↓ , depletion) between observed and expected values based on a permutation test and corrected for multiple tests [139]. Numbers below each cell type indicate the observed counts of expressed genes per age class. The five phylogenetic age classes as defined by Dong and colleagues [78]: class A, genes present before the *Drosophila* radiation; class B, genes originated in the genus *Drosophila*; class C, genes originated in the *Sophophora* subgenus; class D, genes originated in the *melanogaster* species group; class E, genes originated in the *D. melanogaster* species subgroup comprising the *simulans* species complex and *D. melanogaster.* Cell types: EC, epithelial cells; HC, hub cells; CC, cyst cells; ES, germline stem cells and early spermatogonia; LS, late spermatogonia; ESp, early spermatocytes; MSp, mid spermatocytes; LSp, late spermatocytes; MPSp, maturing primary spermatocytes; and S, spermatids.
(TIFF)

S27 Fig Phylogenetic age distribution of genes expressed across ovary cell types. Bar plots show the observed and expected (darker and lighter bars, respectively) percentages of expressed genes in each phylogenetic age class **(A–E)** across ovary cell types for each strain (ISO1 and A4, *D. melanogaster*; *w*^*501*^, *D. simulans*). Class A includes the oldest genes and Class E the youngest. Vertical arrows denote statistically significant deviations (↑, enrichment; ↓ , depletion) between observed and expected values based on a permutation test and corrected for multiple tests [139]. Numbers below each cell type indicate the observed counts of expressed genes per age class. The five phylogenetic age classes as defined by Dong and colleagues [78]: class A, genes present before the *Drosophila* radiation; class B, genes originated in the genus *Drosophila*; class C, genes originated in the *Sophophora* subgenus; class D, genes originated in the *melanogaster* species group; class E, genes originated in the *D. melanogaster* species subgroup comprising the *simulans* species complex and *D. melanogaster.* Cell types: GSC/G1-2a, germline stem cells and germarium region 1 and 2a cells; G2a-2b, germarium region 2a and 2b cells; G2b-3, germarium region 2b and 3 cells; SPC, stalk and polar cells; FSC/preFCs, follicle stem cells and pre-follicle cells; EFC, early follicle cells; MFC, mitotic follicle cells stage 1–5; PMFC, post-mitotic follicle cells stage 6; V7, vitellogenic main-body follicle cells (MBFCs) stage 7; V8, vitellogenic MBFCs stage 8; V9-10A, vitellogenic MBFCs stage 9-10A; C12, choriogenic MBFCs stage 12; C14, choriogenic MBFCs stage 14; TCLC, terminal corpus luteum cells; SC, stretch cells; OSM, ovarian sheath muscle; and O, oviduct.
(TIFF)

S28 Fig Selection pressure on gene expression divergence at the cell type level. Box plots displaying three metrics of evolutionary change at sequence level of the genes expressed across cell types and strains (A4 and ISO1, *D. melanogaster; w*^*501*^, *D. simulans*). The plots show: **(A, B)** the ratio of nonsynonymous to synonymous substitutions (ω, left); **(C, D)** the adaptive component of the ratio of protein evolution (ω_a,_ center); and **(E, F)** the nonadaptive component of the rate of protein evolution (ω_na_, right). Testis, top; ovary, bottom. Boxes represent the interquartile range around the median (black horizontal line) and whiskers extend to 1.5 times the IQR. Each point represents the value of a particular gene, and values are grouped by strain and cell type. Gene counts for each combination (n) are shown above each boxplot. Testis cell types: EC, epithelial cells; HC, hub cells; CC, cyst cells; ES, germline stem cells and early spermatogonia; LS, late spermatogonia; ESp, early spermatocytes; MSp, mid spermatocytes; LSp, late spermatocytes; MPSp, maturing primary spermatocytes; and S, spermatids. Ovary cell types: GSC/G1-2a, germline stem cells and germarium region 1 and 2a cells; G2a-2b, germarium region 2a and 2b cells; G2b-3, germarium region 2b and 3 cells; SPC, stalk and polar cells; FSC/preFCs, follicle stem cells and pre-follicle cells; EFC, early follicle cells; MFC, mitotic follicle cells stage 1–5; PMFC, post-mitotic follicle cells stage 6; V7, vitellogenic main-body follicle cells (MBFCs) stage 7; V8, vitellogenic MBFCs stage 8; V9-10A, vitellogenic MBFCs stage 9-10A; C12, choriogenic MBFCs stage 12; C14, choriogenic MBFCs stage 14; TCLC, terminal corpus luteum cells; SC, stretch cells; OSM, ovarian sheath muscle; and O, oviduct.
(TIFF)

S29 Fig Adaptive component of selection pressure on gene expression divergence at the cell type level for *X*-linked and autosomal genes. Box plots displaying the adaptive component of the ratio of protein evolution (ω_a_) at sequence level for the genes expressed across cell types and strains (A4 and ISO1, *D. melanogaster; w*^*501*^, *D. simulans*) split by **(A, B)** *X*-linked and **(C, D)** autosomal genes in the testis (top) and ovary (bottom). Boxes represent the interquartile range around the median (black horizontal line) and whiskers extend to 1.5 times the IQR. Each point represents the ω_a_ value of a particular gene, and values are grouped by strain and cell type. Testis cell types: EC, epithelial cells; HC, hub cells; CC, cyst cells; ES, germline stem cells and early spermatogonia; LS, late spermatogonia; ESp, early spermatocytes; MSp, mid spermatocytes; LSp, late spermatocytes; MPSp, maturing primary spermatocytes; and S, spermatids. Ovary cell types: GSC/G1-2a, germline stem cells and germarium region 1 and 2a cells; G2a-2b, germarium region 2a and 2b cells; G2b-3, germarium region 2b and 3 cells; SPC, stalk and polar cells; FSC/preFCs, follicle stem cells and pre-follicle cells; EFC, early follicle cells; MFC, mitotic follicle cells stage 1–5; PMFC, post-mitotic follicle cells stage 6; V7, vitellogenic main-body follicle cells (MBFCs) stage 7; V8, vitellogenic MBFCs stage 8; V9-10A, vitellogenic MBFCs stage 9-10A; C12, choriogenic MBFCs stage 12; C14, choriogenic MBFCs stage 14; TCLC, terminal corpus luteum cells; SC, stretch cells; OSM, ovarian sheath muscle; and O, oviduct.
(TIFF)

S30 Fig Genomic distribution of interspecifically diverged genes between *D. melanogaster* and *D. simulans.* **(A)** Chromosome distribution of DEGs between species. Only genes that showed consistent expression differences across the two interspecific comparisons (*i.e.,* A4 versus *w*^*501*^ and ISO1 versus *w*^*501*^) for at least one same cell type and not located on the dot-like chromosome *4*, are considered. DEGs only in testis, *n* = 842, only in ovary, *n* = 431, in both tissues, n = 155. The karyotype was generated using the R package karyoploteR [148]. **(B)** Percentage of expected versus observed DEGs for each category (testis-exclusive, ovary-exclusive, shared by both tissues) across different chromosomes. The probability of finding a difference between both distributions was determined by a goodness-of-fit chi-squared test (in parenthesis). Post-hoc tests revealed which chromosome contributes to the global statistical difference found. *P*-values were adjusted for multiple tests correction [139].
(TIFF)

S31 Fig Interstrain expression divergence and intersex expression correlation across chromosomes and tissues at the pseudo-bulk level. **(A)** Pairwise Spearman’s Rho correlations of gene expression across chromosomes are shown for three strain comparisons and two tissues, testis and ovary. In the ovary but not in the testis, there is evidence of faster-*X* evolution in gene expression (i.e., lower correlation) in the two interspecific contrasts. For each graph and chromosome, the dot corresponds to the correlation value and the error bar is the 95% CI. In each graph, the genome-wide correlation is shown as a red dashed line while the red dotted lines correspond to the genome-wide 95% CI. Only the four rod-like autosomes (the left and right arms -L and R- of chromosomes *2* and *3*) are considered. **(B)** Spearman’s Rho correlations of gene expression across chromosomes between expression levels in testis and ovaries are shown for three strains. Gene expression on the *X* chromosome is not subject to more relaxed functional constraints compared to the autosomes as shown by the highest correlation values of the *X* versus the autosomes. Strains: A4 and ISO1, *D. melanogaster; w*^*501*^, *D. simulans*.
(TIFF)

S32 Fig Cell cluster relationships across clustering resolutions in the testis and ovary. Clustree plots show the relationships among clusters across increasing clustering resolutions (0–2.0, in increments of 0.2) for **(A)** testis and **(B)** ovary. Each node represents a cluster identified at a given resolution, and edges connect clusters across adjacent resolutions based on shared cells. These plots were used to assess cluster stability and subdivision across resolutions and to guide selection of an appropriate resolution for downstream annotation and analysis.
(TIFF)

S1 Text Analysis of cell type proportions across strains. (DOCX)

S1 Table Salient stats of the snRNA-seq experiments performed in *Drosophila* gonadal tissues. (XLSX)

S2 Table Comparison across scRNA-seq studies in the testis. (XLSX)

S3 Table Comparison across scRNA-seq studies in the ovary. (XLSX)

S4 Table List of marker genes used to annotate testis and ovary cell types. (XLSX)

S5 Table Results of linear models to test for differences in cell type composition across pairwise comparisons of *Drosophila* strains. (XLSX)

S6 Table Correlation coefficients for the percentage of different cell types between any two *Drosophila* strains surveyed. (XLSX)

S7 Table Test for differences in the number of expressed genes between cell types across the strains and tissues considered. (XLSX)

S8 Table Test for differences in within-strain expression correlations across broad-cell type categories in the testis and ovary. (XLSX)

S9 Table Test for differences in within-strain expression correlations between different broad cell type categories across strains and tissues. (XLSX)

S10 Table Test for differences in between-strain expression correlation values within and between species across tissues. (XLSX)

S11 Table Test for differences across broad cell type categories in the testis and across between-strain contrasts. (XLSX)

S12 Table Test for differences across broad cell type categories in the ovary and across between-strain contrasts. (XLSX)

S13 Table Linear relationship metrics between the number of DEGs and different cell types across different pairwise comparisons. (XLSX)

S14 Table Test of differences in the proportion of differentially expressed genes between the strains of *D. melanogaster* and *D. simulans* that are shared at the cell type level across cell types within a given tissue. (XLSX)

S15 Table Test for differences in number of DEGs detected at the cell type level between tissues and type of contrast. (XLSX)

S16 Table Salient features of coexpression modules in testis and ovary. (XLSX)

S17 Table Gene ontology (GO) enrichment analysis of top 10 hub genes across coexpression modules of the testis and ovary. (XLSX)

S18 Table Gene ontology (GO) enrichment analysis of species-divergent coexpression modules of the testis. (XLSX)

S19 Table Gene ontology (GO) enrichment analysis of species-divergent coexpression modules of the ovary. (XLSX)

S20 Table Tests for differences in tissue specificity for the genes expressed between cell types and across strains and tissues. (XLSX)

S21 Table Tests for differences in cell-type specificity for the genes expressed between cell types across strains and tissues. (XLSX)

S22 Table Tests for differences in sequence evolution metrics of the genes expressed between testis cell types across the strains considered. (XLSX)

S23 Table Tests for differences in sequence evolution metrics of the genes expressed between ovary cell types across the strains. (XLSX)

S24 Table Test for deviations in the distribution of DEG genes between *D. melanogaster* and *D. simulans* across chromosomes. (XLSX)

S25 Table Test for differences in interstrains expression correlations per chromosome across testis and ovary cell types. (XLSX)

S1 Data Average expression of genes per cell type and strain in the testis. (XLSX)

S2 Data Average expression of genes per cell type and strain in the ovary. (XLSX)

S3 Data Differential expression of genes per cell type and strain in the testis. (XLSX)

S4 Data Differential expression of genes per cell type and strain in the ovary. (XLSX)

S5 Data Data underlying S17 Fig.
(XLSX)

S6 Data Data underlying S18 Fig.
(XLSX)

S7 Data Coexpression network analysis across testis cell types and strains. (XLSX)

S8 Data Coexpression network analysis across ovary cell types and strains. (XLSX)

S9 Data Data underlying S23 Fig.
(XLSX)

## Abbreviations

ART: aligned rank transform
CC: cyst cells
C12: choriogenic MBFCs stage 12
C14: choriogenic MBFCs stage 14
DE: differentially expressed
DEGs: differentially expressed genes
DME: differential module eigengene
EC: epithelial cells
EFC: early follicle cells
ES: early spermatogonia
EpS: epithelium somatic
ESp: early spermatocytes
FCA: Fly Cell Atlas
FSC/preFCs: follicle stem cells and pre-follicle cells
G: germline
GO: gene ontology
GS: germarium somatic
G2a-2b: germarium region 2a and 2b cells
G2b-3: germarium region 2b and 3 cells
GSC/G1-2a: germline stem cells and germarium region 1 and 2a cells
HC: hub cells
LS: late spermatogonia
LSp: late spermatocytes
MBFCs: main-body follicle cells
Me: meiotic/post-meiotic
MEs: module eigengenes
MFC: mitotic follicle cells stage 1-5
Mi: mitotic
MSp: mid spermatocytes
MPSp: maturing primary spermatocytes
MSCI: meiotic sex chromosome inactivation
O: oviduct
OSM: ovarian sheath muscle
PCA: Principal component analysis
PMFC: post-mitotic follicle cells stage 6
ROIs: regions of interest
S: somatic
S: spermatids
SC: stretch cells
SPC: stalk and polar cells
TCLC: terminal corpus luteum cells
TE: transposable element
TOM: topological overlap matrix
UMAP: uniform manifold approximation and projection.
Un: unannotated
V7: vitellogenic main-body follicle cells (MBFCs) stage 7
V8: vitellogenic MBFCs stage 8
V9-10A: vitellogenic MBFCs stage 9-10A.
