# Supplementary figures and images for: Comparative single-nucleus transcriptomics reveals asymmetric evolution of the Drosophila male and female germlines
Source: PLoS Biol. 2026 Jul 20;24(7):e3003869. doi: 10.1371/journal.pbio.3003869 (PMC13384527; doi:10.1371/journal.pbio.3003869)

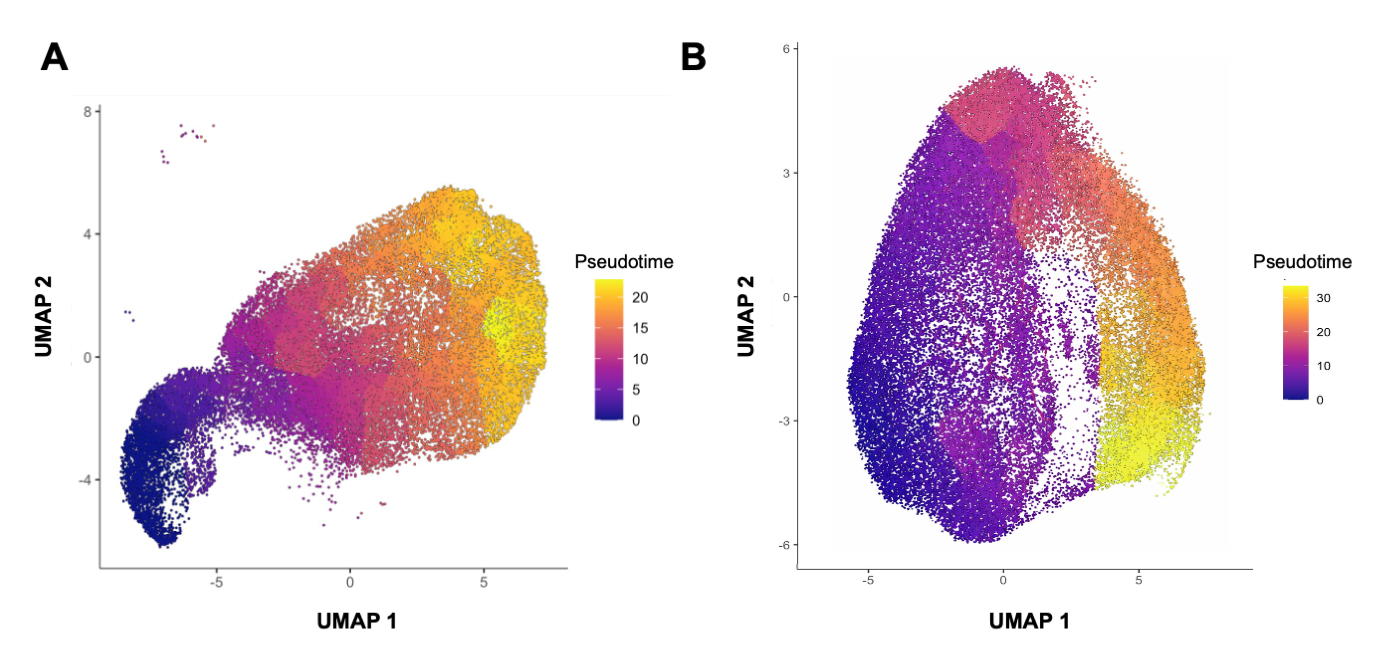

Supplement: S1 Fig — UMAPs of the (A) testis and (B) ovary, colored by pseudotime values as inferred using Monocle 3 [135]. In both tissues, pseudotime recapitulates the expected developmental progression across gametogenesis, with early germline or progenitor populations occupying lower pseudotime values and more differentiated cell states distributed along later pseudotime regions of the embedding. The overall distribution of pseudotime is consistent with the annotated developmental hierarchy of testis and ovary cell types. (TIFF) [file pbio.3003869.s001.tiff]

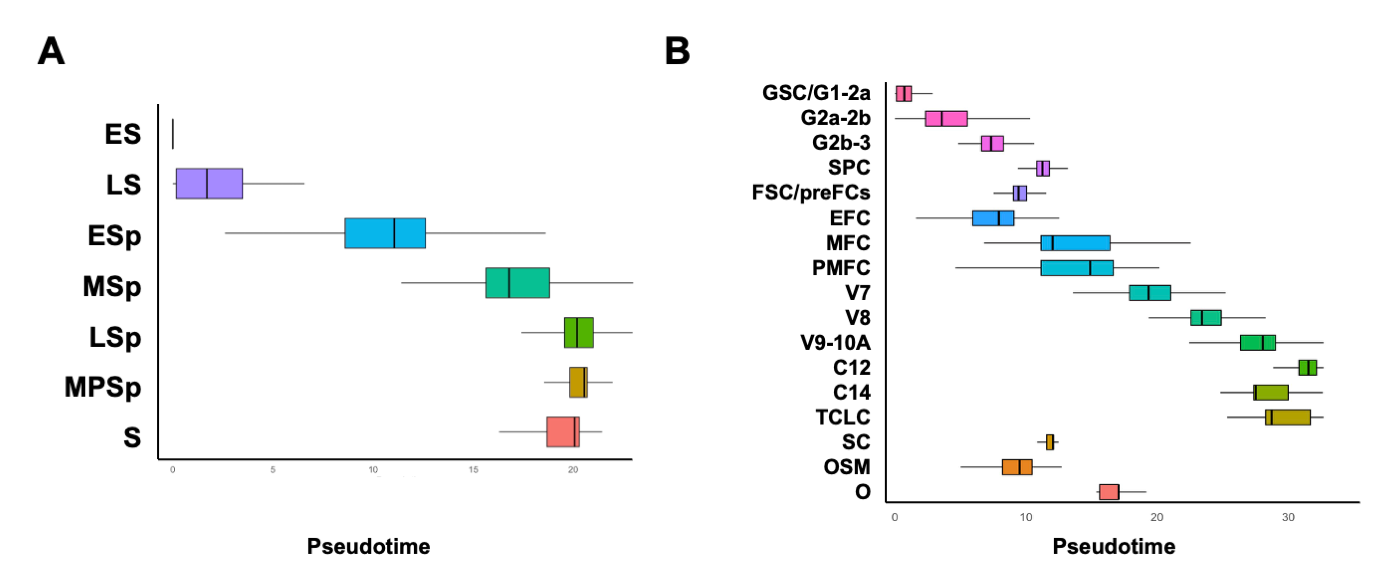

Supplement: S2 Fig — (A, B) Boxplots show the distribution of pseudotime values for cells annotated as specific cell types, ordered vertically to reflect the inferred developmental progression for (A) testis and (B) ovary, based on Monocle3 analysis. In both tissues, pseudotime recapitulates known differentiation hierarchies, with early germline or stem cell populations occupying lower pseudotime values and differentiated somatic or germline cells positioned later. (TIFF) [file pbio.3003869.s002.tiff]

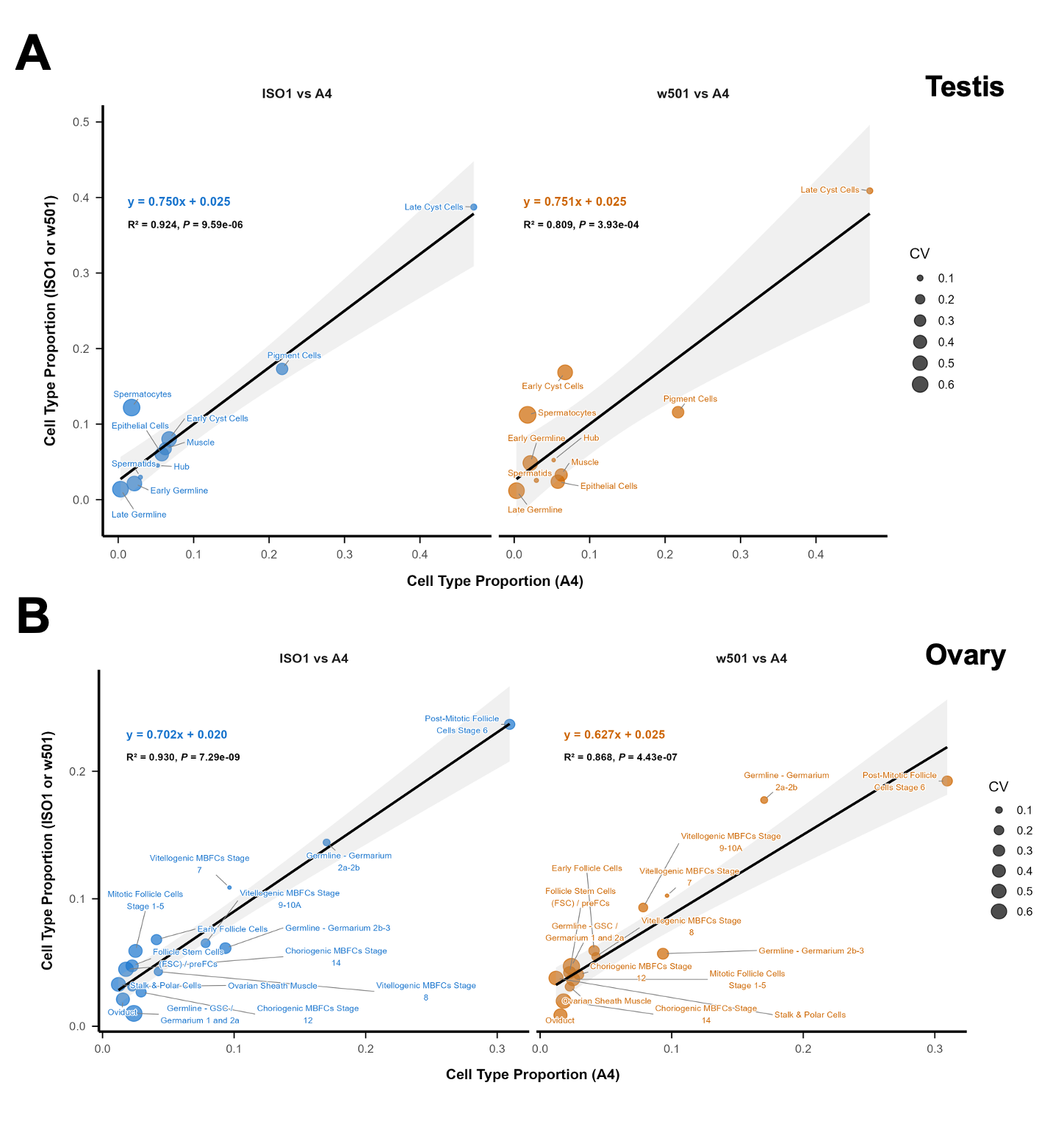

Supplement: S3 Fig — (A, B) Best-fit regression lines for the cell type proportions detected with snRNA-seq between A4 and two other strains (ISO1, blue; and w501, orange) are shown with their corresponding equations, coefficients of determination, and statistical significance of the latter for the testis and ovary, respectively. ISO1 and A4, D. melanogaster; w501, D. simulans. Cell clusters that were unannotated or did not fulfill the requirement of at least 50 nuclei per strain and 350 across the three strains (i.e., terminal corpus luteum cells (TCLC) and stretch cells (SC) in the ovary), were not considered in the analysis. The diameter of each datapoint denotes the coefficient of variation (CV) for the proportion of a particular cell type across the three strains considered. The 95% confidence intervals are shaded. (TIFF) [file pbio.3003869.s003.tiff]

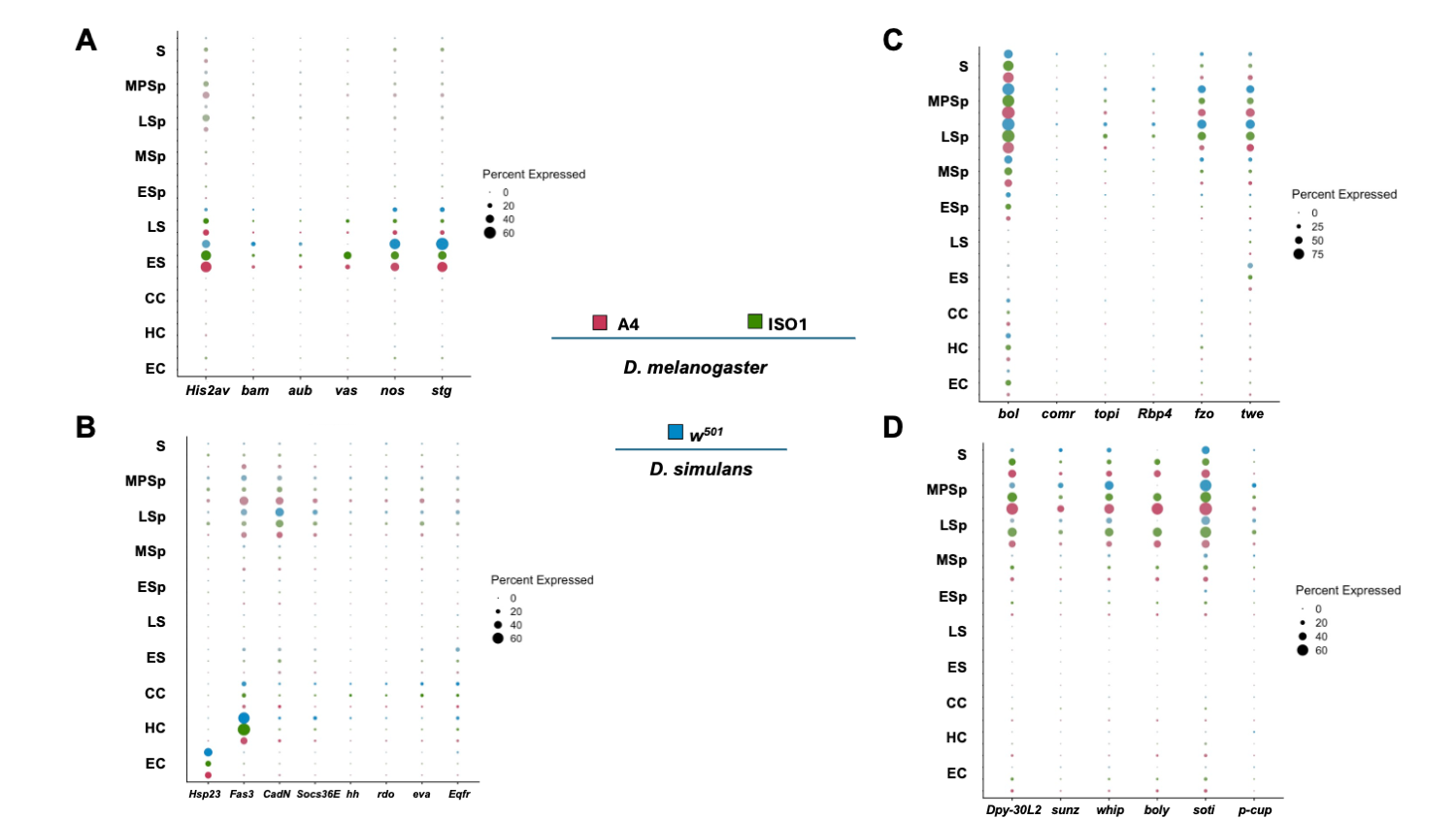

Supplement: S4 Fig — Dot plots showing the average gene expression of marker genes in different testis cell types: (A) mitotic cells – spermatogonia (ES and LS); (B) somatic cells (HC, CC, and EC); (C) meiotic cells – spermatocytes (ESp, MSp, LSp, and MPSp); and (D) spermatids (S). The size of each dot represents the proportion of cells in which the gene is expressed. Gene expression values are normalized and scaled, with darker solid colors indicating overexpression relative to the average and lighter gray shades indicating underexpression. EC, epithelial cells; HC, hub cells; CC, cyst cells; ES, germline stem cells and early spermatogonia; LS, late spermatogonia; ESp, early spermatocytes; MSp, mid spermatocytes; LSp, late spermatocytes; MPSp, maturing primary spermatocytes; and S, spermatids. (TIFF) [file pbio.3003869.s004.tiff]

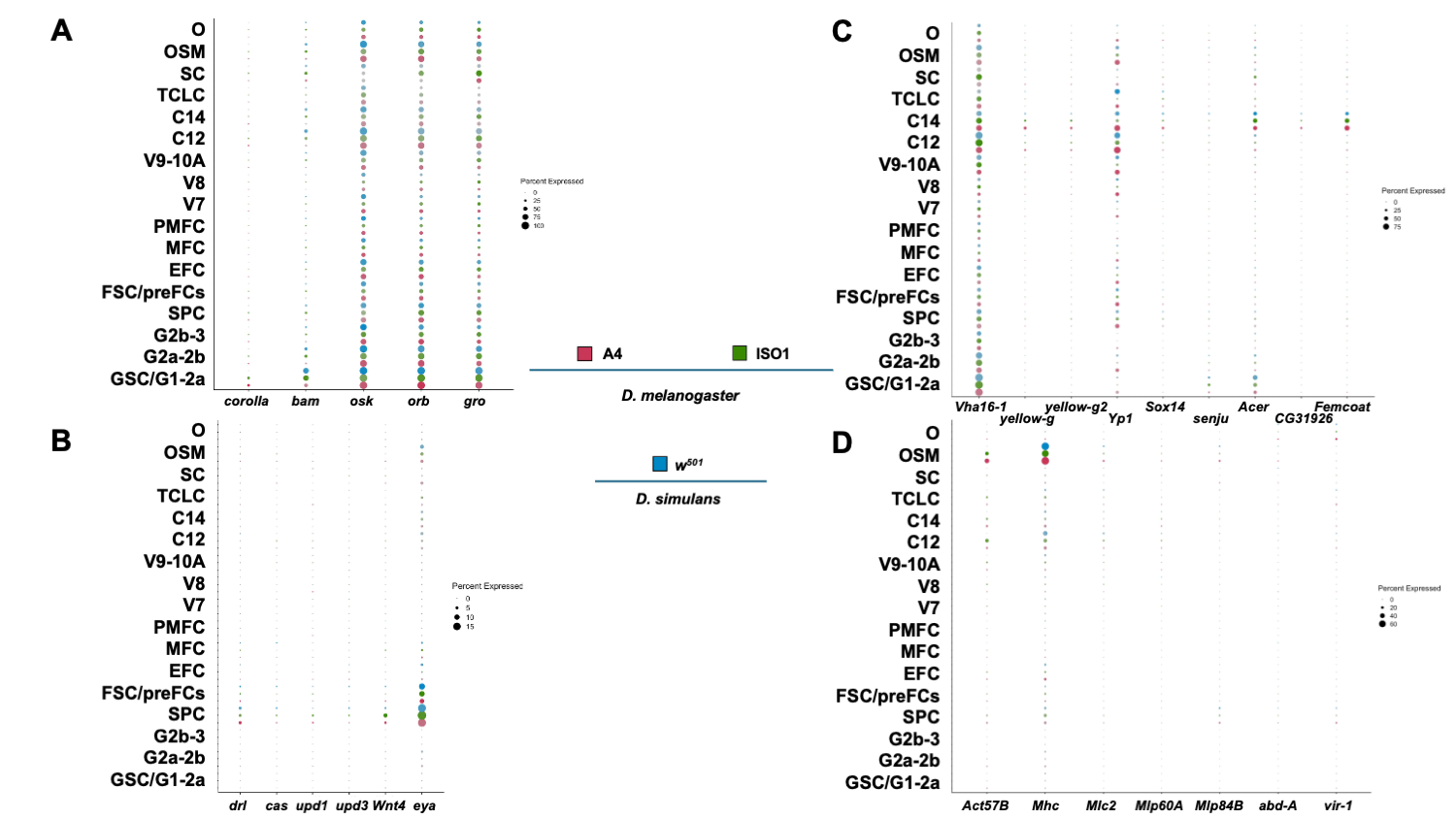

Supplement: S5 Fig — Dot plots showing the average gene expression of marker genes in different ovary cell types: (A) germline cells (G1-2a, G2a-2b and G2b-3); (B) germarium somatic cells (SPC, FSC/preFCs, EFC, MFC and PMFC); (C) epithelium somatic cells – main body follicle cells (V7, V8, V9-10A, C12 and C14); and (D) ovarian sheath muscle (OSM) and oviduct (O). The size of each dot represents the proportion of cells in which the gene is expressed. Gene expression values are normalized and scaled, with darker solid colors indicating overexpression and lighter gray shades indicating underexpression. Cell types: GSC/G1-2a, germline stem cells and germarium region 1 and 2a cells; G2a-2b, germarium region 2a and 2b cells; G2b-3, germarium region 2b and 3 cells; SPC, stalk and polar cells; FSC/preFCs, follicle stem cells and pre-follicle cells; EFC, early follicle cells; MFC, mitotic follicle cells stage 1–5; PMFC, post-mitotic follicle cells stage 6; V7, vitellogenic main-body follicle cells (MBFCs) stage 7; V8, vitellogenic MBFCs stage 8; V9-10A, vitellogenic MBFCs stage 9-10A; C12, choriogenic MBFCs stage 12; C14, choriogenic MBFCs stage 14; TCLC, terminal corpus luteum cells; SC, stretch cells; OSM, ovarian sheath muscle; and O, oviduct. (TIFF) [file pbio.3003869.s005.tiff]

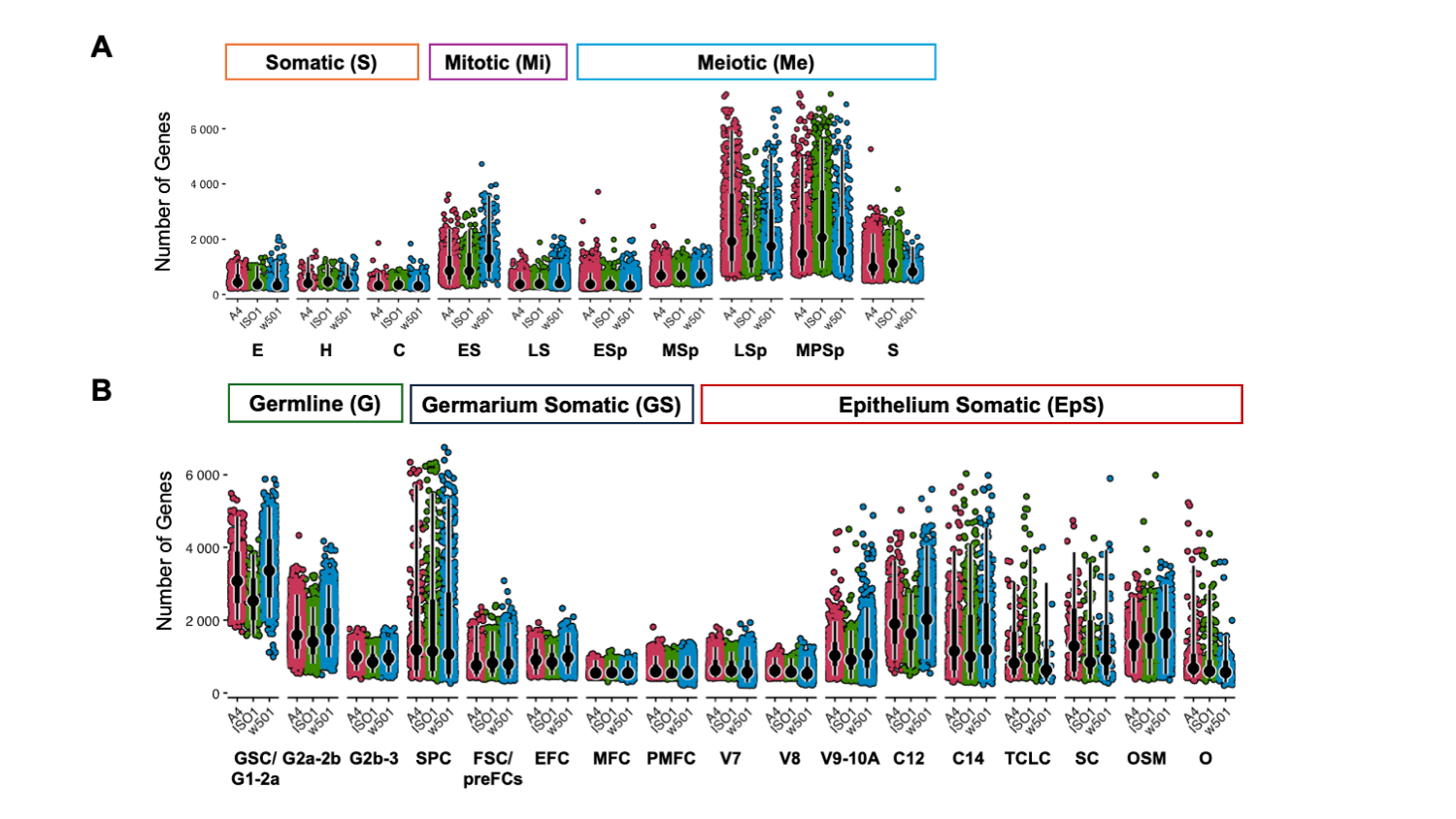

Supplement: S6 Fig — (A, B) Geyser plots showing the number of expressed genes detected across the cells within particular cell types for the three strains assayed (A4 and ISO1, D. melanogaster: w501, D. simulans) in the testis and ovary, respectively. Broad categories of cell types for each tissue are indicated on top. Within each plot, the median, the 66% (thick line) and the 95% (thin line) of the data are shown. Testis cell types: EC, epithelial cells; HC, hub cells; CC, cyst cells; ES, germline stem cells and early spermatogonia; LS, late spermatogonia; ESp, early spermatocytes; MSp, mid spermatocytes; LSp, late spermatocytes; MPSp, maturing primary spermatocytes; and S, spermatids. Ovary cell types: GSC/G1-2a, germline stem cells and germarium region 1 and 2a cells; G2a-2b, germarium region 2a and 2b cells; G2b-3, germarium region 2b and 3 cells; SPC, stalk and polar cells; FSC/preFCs, follicle stem cells and pre-follicle cells; EFC, early follicle cells; MFC, mitotic follicle cells stage 1–5; PMFC, post-mitotic follicle cells stage 6; V7, vitellogenic main-body follicle cells (MBFCs) stage 7; V8, vitellogenic MBFCs stage 8; V9-10A, vitellogenic MBFCs stage 9-10A; C12, choriogenic MBFCs stage 12; C14, choriogenic MBFCs stage 14; TCLC, terminal corpus luteum cells; SC, stretch cells; OSM, ovarian sheath muscle; and O, oviduct. (TIFF) [file pbio.3003869.s006.tiff]

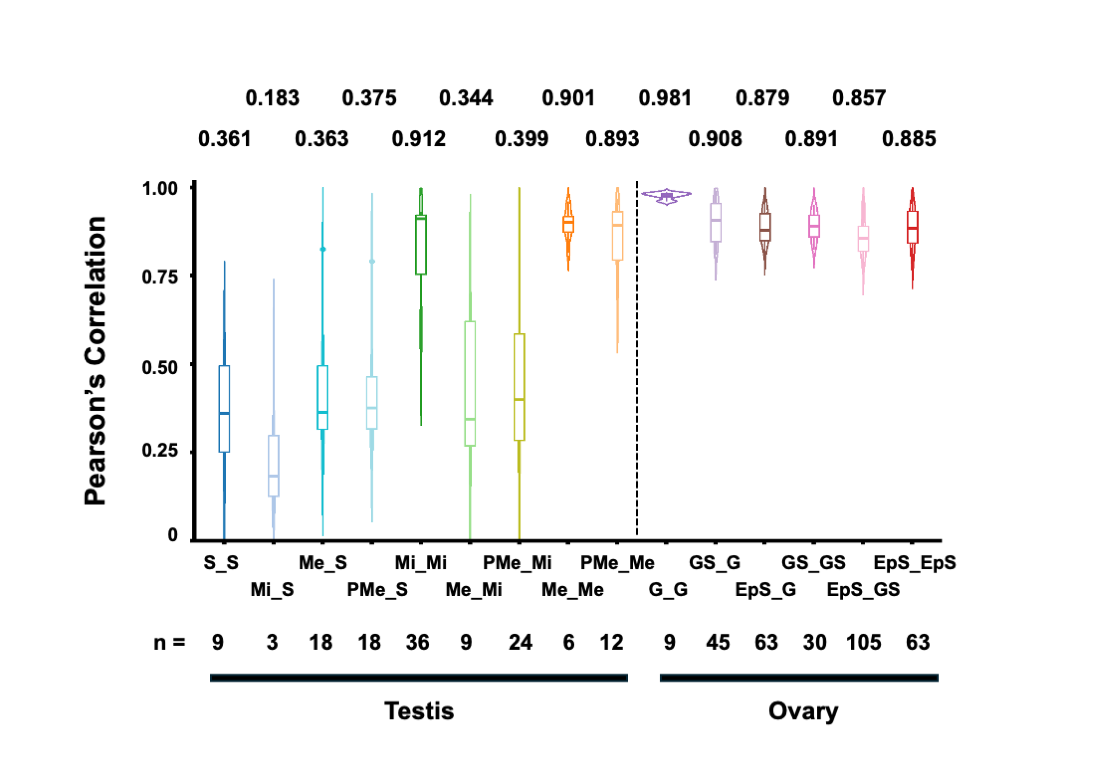

Supplement: S7 Fig — Violin (foreground) and box (background) plots showing the distribution of within-strain expression correlations across contrasts between broad cell-type categories in the testis and ovary. Boxes represent the interquartile range (IQR) around the median (horizontal line), and whiskers extend to 1.5 times the IQR. Median correlation values, top; number of correlation values considered (n), bottom. The outcomes of the post hoc tests can be found in S8 Table. Testis broad cell-type categories: Me, meiotic; Mi, mitotic; PMe, post-meiotic; and S, somatic. Ovary broad cell-type categories: EpS, epithelium somatic; G, germline; and GS, germarium somatic. (TIFF) [file pbio.3003869.s007.tiff]

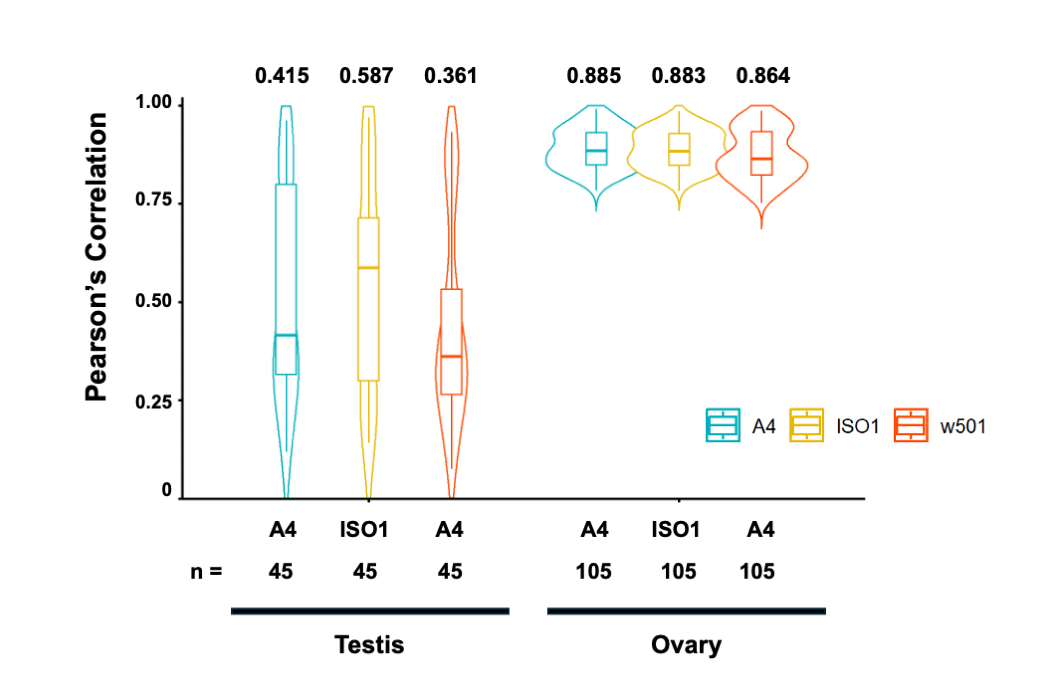

Supplement: S8 Fig — Violin (foreground) and box (background) plots showing the distribution of within-strain expression correlation values between pairs of cell types in the testis and ovary across the three strains assayed. Boxes represent the interquartile range (IQR) around the median (horizontal line), and whiskers extend to 1.5 times the IQR. Median correlation values, top; number of correlation values considered (n), bottom. The outcomes of the post hoc tests can be found in S9 Table. Strains: D. melanogaster (A4, ISO1); D. simulans (w501). (TIFF) [file pbio.3003869.s008.tiff]

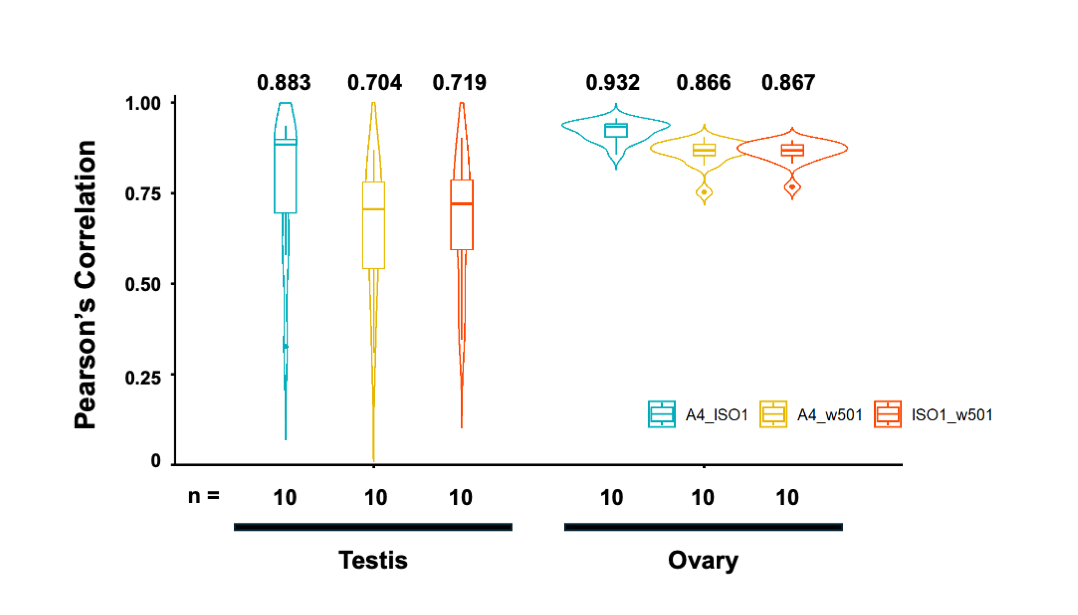

Supplement: S9 Fig — Violin (foreground) and box (background) plots showing the distribution of between-strain expression correlation values for equivalent cell types in the testis and ovary across the three pairwise strain contrasts. Boxes represent the interquartile range (IQR) around the median (horizontal line), and whiskers extend to 1.5 times the IQR. Median correlation values, top; number of correlation values considered (n), bottom. The outcomes of the post hoc tests can be found in S10 Table. Strains: D. melanogaster (A4, ISO1); D. simulans (w501). (TIFF) [file pbio.3003869.s009.tiff]

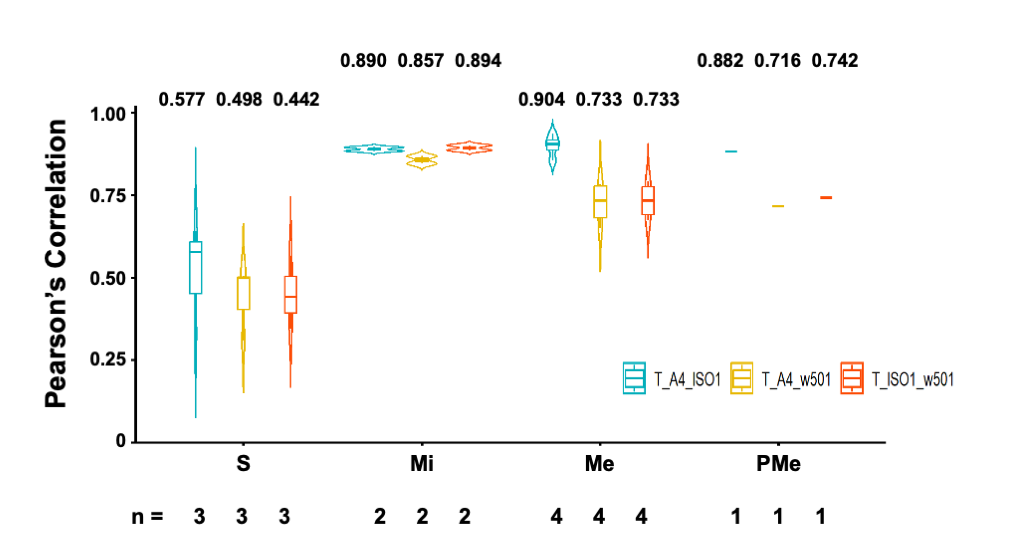

Supplement: S10 Fig — Violin (foreground) and box (background) plots showing the distribution of between-strain expression correlation values across three pairwise strain contrasts and four broad cell-type categories in the testis. Boxes represent the interquartile range (IQR) around the median (horizontal line), and whiskers extend to 1.5 times the IQR. Median correlation values, top; number of correlation values considered (n), bottom. The outcomes of the post hoc tests can be found in S11 Table. Testis broad cell-type categories: Me, meiotic; Mi, mitotic; PMe, post-meiotic; and S, somatic. Strains: D. melanogaster (A4, ISO1); D. simulans (w501). T, testis. (TIFF) [file pbio.3003869.s010.tiff]

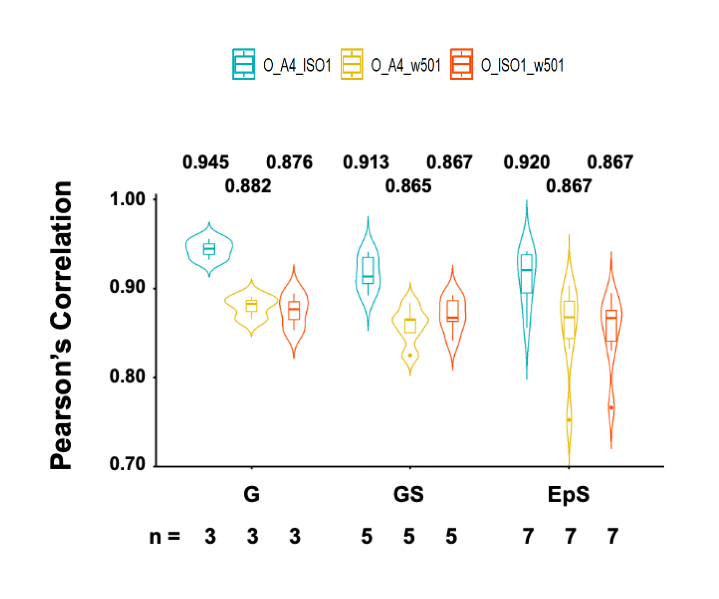

Supplement: S11 Fig — Violin (foreground) and box (background) plots showing the distribution of between-strain expression correlation values across three pairwise strain contrasts and three broad cell-type categories in the ovary. Boxes represent the interquartile range (IQR) around the median (horizontal line), and whiskers extend to 1.5 times the IQR. Median correlation values, top; number of correlation values considered (n), bottom. The outcomes of the post hoc tests can be found in S12 Table. Ovary broad cell-type categories: EpS, epithelium somatic; G, germline; and GS, germarium somatic. Strains: D. melanogaster (A4, ISO1); D. simulans (w501). O, ovary. (TIFF) [file pbio.3003869.s011.tiff]

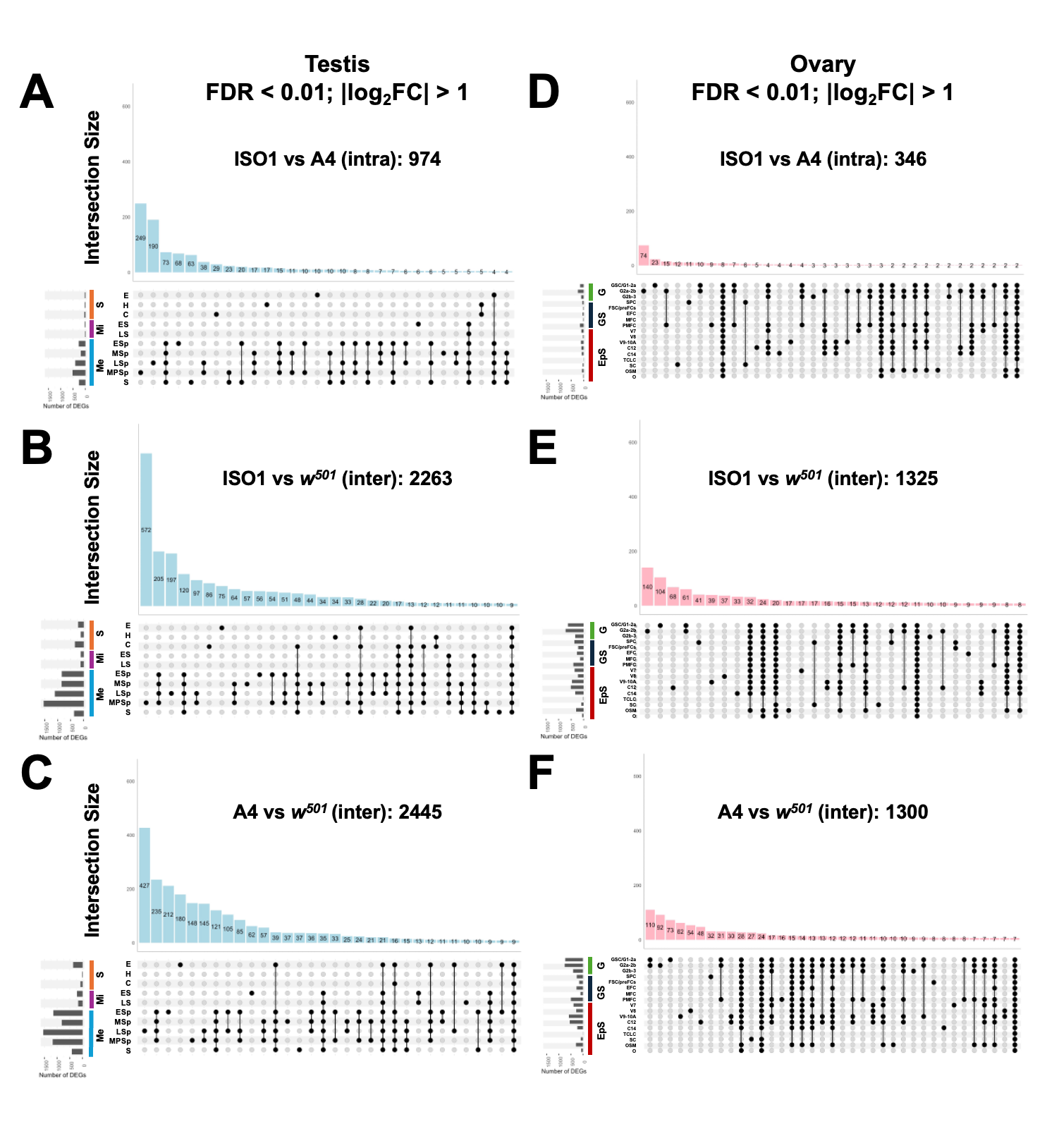

Supplement: S12 Fig — UpSet plots depicting differential expression patterns at different phylogenetic scales for testis and ovary comparisons. Testis: (A) D. melanogaster ISO1 versus A4 (intraspecific); (B) D. melanogaster ISO1 versus D. simulans w501 (interspecific); and (C) D. melanogaster A4 versus D. simulans w501 (interspecific). Ovary: (D) D. melanogaster ISO1 versus A4; (E) D. melanogaster ISO1 versus D. simulans w501; and (F) D. melanogaster A4 versus D. simulans w501. A bar graph is plotted alongside each UpSet plot to depict the number of differentially expressed genes detected per cell type. Broad cell type categories are indicated: testis – somatic (S), mitotic (Mi), and meiotic (Me); and ovary – germline (G), germarium somatic (GS), and epithelium somatic (EpS). (TIFF) [file pbio.3003869.s012.tiff]

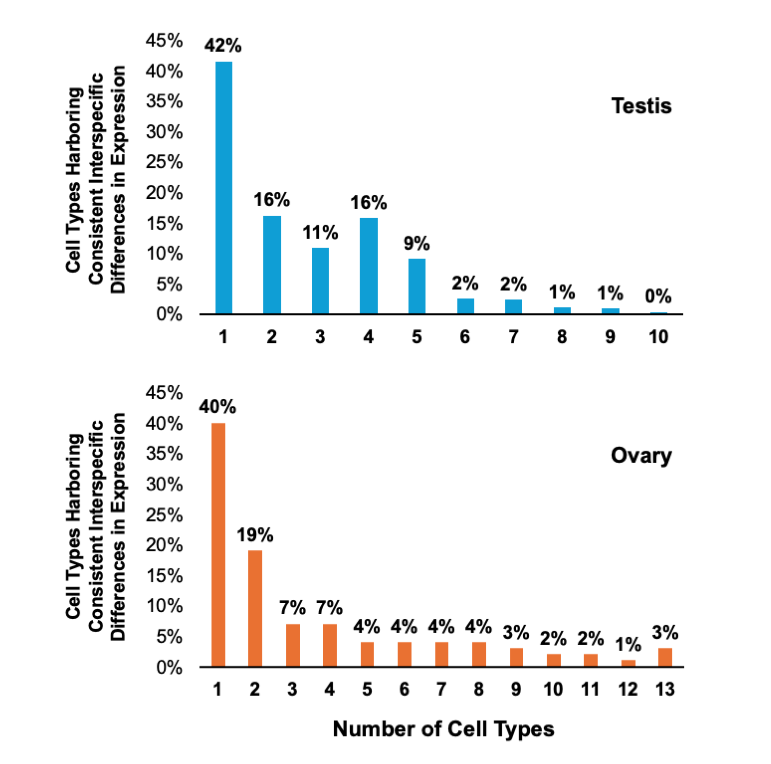

Supplement: S13 Fig — Fraction of differentially expressed genes (DEGs) between species, categorized by the number of different cell types in which both D. melanogaster strains (ISO1, A4) differ from D. simulans w501. (TIFF) [file pbio.3003869.s013.tiff]

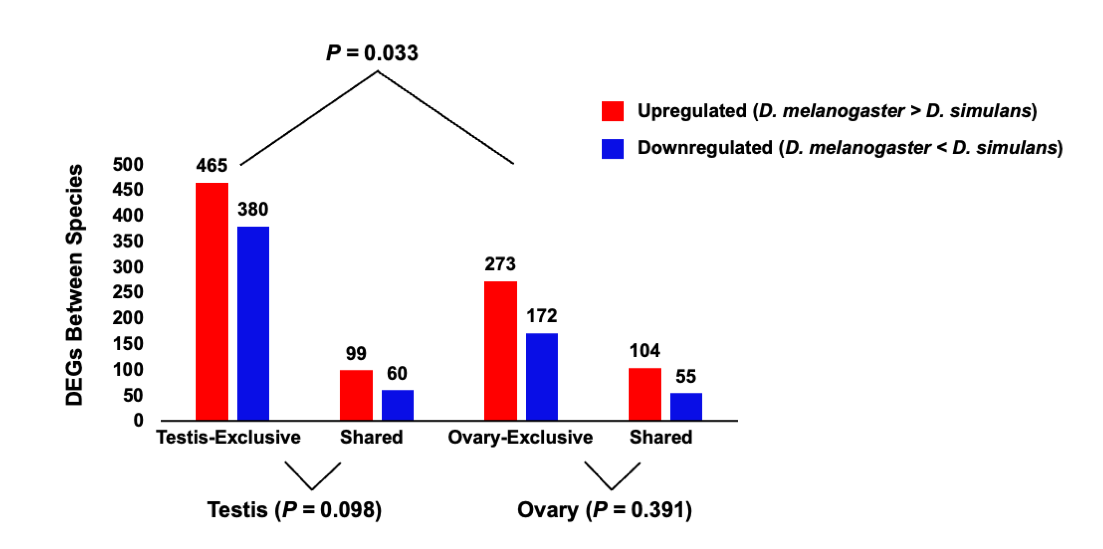

Supplement: S14 Fig — Up- and downregulation refer to significantly higher and lower expression levels in D. melanogaster versus D. simulans, respectively. Differentially expressed genes (DEGs) exclusively in testis, or ovary, do not tend to be more upregulated in D. melanogaster compared to D. simulans relative to DEGs in both tissues, as determined by Fisher’s Exact Tests (p-values shown for each contrast). When focusing on DEGs exclusively in one tissue, there is some evidence of a higher tendency of upregulation in D. melanogaster relative to D. simulans for those detected only in testis versus those detected only in ovary. (TIFF) [file pbio.3003869.s014.tiff]

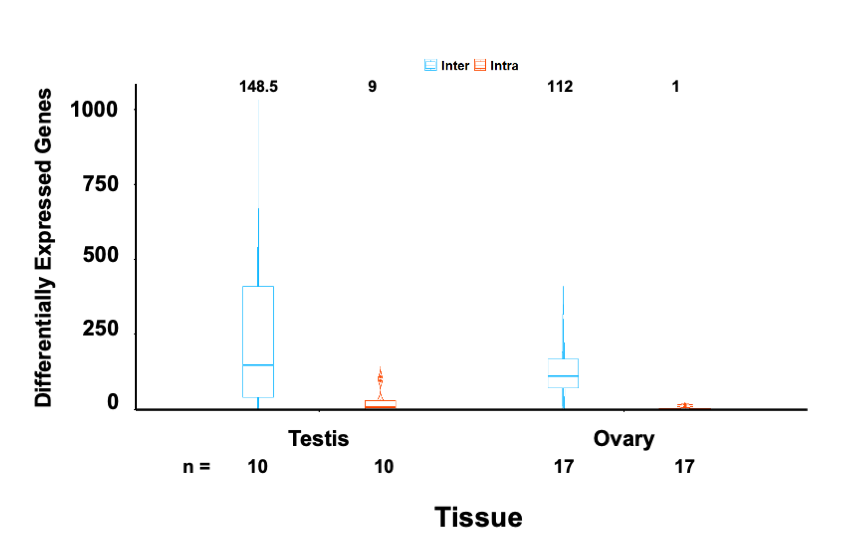

Supplement: S15 Fig — Violin (foreground) and box (background) plots showing the distribution of differentially expressed genes (DEGs) at the inter and intraspecific levels. At the intraspecific level, genes differentially expressed at the cell type level between the two strains of D. melanogaster (ISO1, A4) were considered. At the interspecific level, only genes consistently found differentially expressed in the same cell type of the two strains of D. melanogaster relative to one of D. simulans (w501) were considered. Boxes represent the interquartile range (IQR) around the median (horizontal line), and whiskers extend to 1.5 times the IQR. Median DEG counts, top; number of cell types considered (n), bottom. The outcomes of the post hoc tests can be found in S15 Table. (TIFF) [file pbio.3003869.s015.tiff]

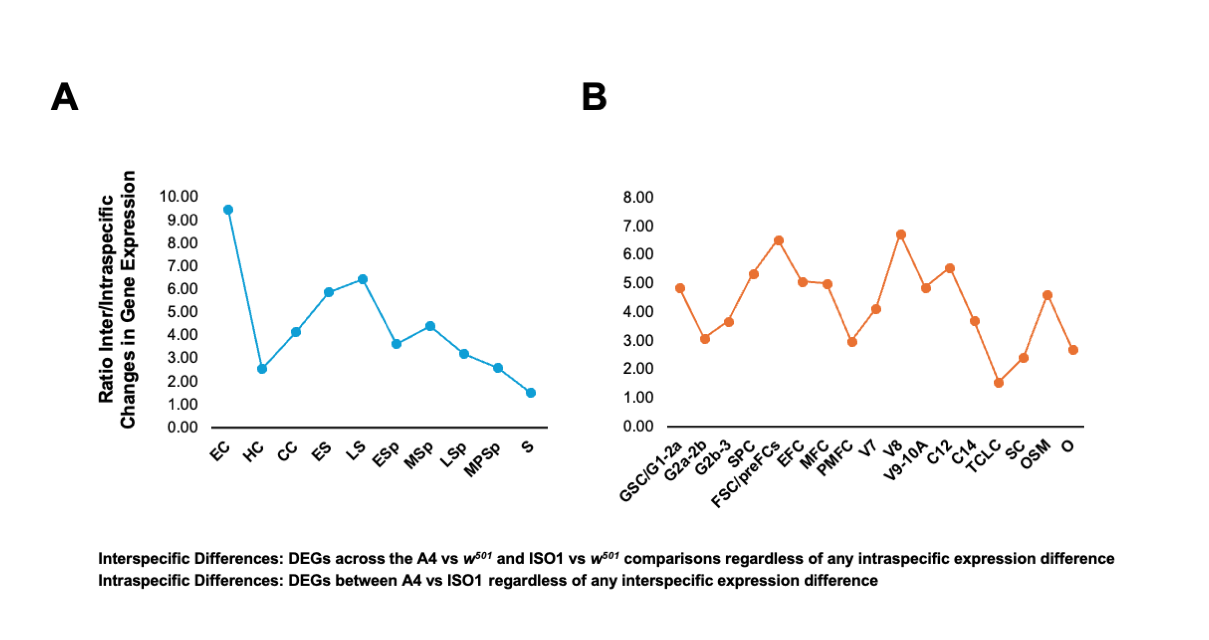

Supplement: S16 Fig — (A, B) Testis and ovary, respectively. Ratio of the average number of DEGs in interspecific comparisons (D. melanogaster A4 versus D. simulans w501, D. melanogaster ISO1 versus D. simulans w501) to the number of DEGs between the two strains of D. melanogaster (A4 versus ISO1). Testis cell types: EC, epithelial cells; HC, hub cells; CC, cyst cells; ES, germline stem cells and early spermatogonia; LS, late spermatogonia; ESp, early spermatocytes; MSp, mid spermatocytes; LSp, late spermatocytes; MPSp, maturing primary spermatocytes; and S, spermatids. Ovary cell types: GSC/G1-2a, germline stem cells and germarium region 1 and 2a cells; G2a-2b, germarium region 2a and 2b cells; G2b-3, germarium region 2b and 3 cells; SPC, stalk and polar cells; FSC/preFCs, follicle stem cells and pre-follicle cells; EFC, early follicle cells; MFC, mitotic follicle cells stage 1–5; PMFC, post-mitotic follicle cells stage 6; V7, vitellogenic main-body follicle cells (MBFCs) stage 7; V8, vitellogenic MBFCs stage 8; V9-10A, vitellogenic MBFCs stage 9-10A; C12, choriogenic MBFCs stage 12; C14, choriogenic MBFCs stage 14; TCLC, terminal corpus luteum cells; SC, stretch cells; OSM, ovarian sheath muscle; and O, oviduct. (TIFF) [file pbio.3003869.s016.tiff]

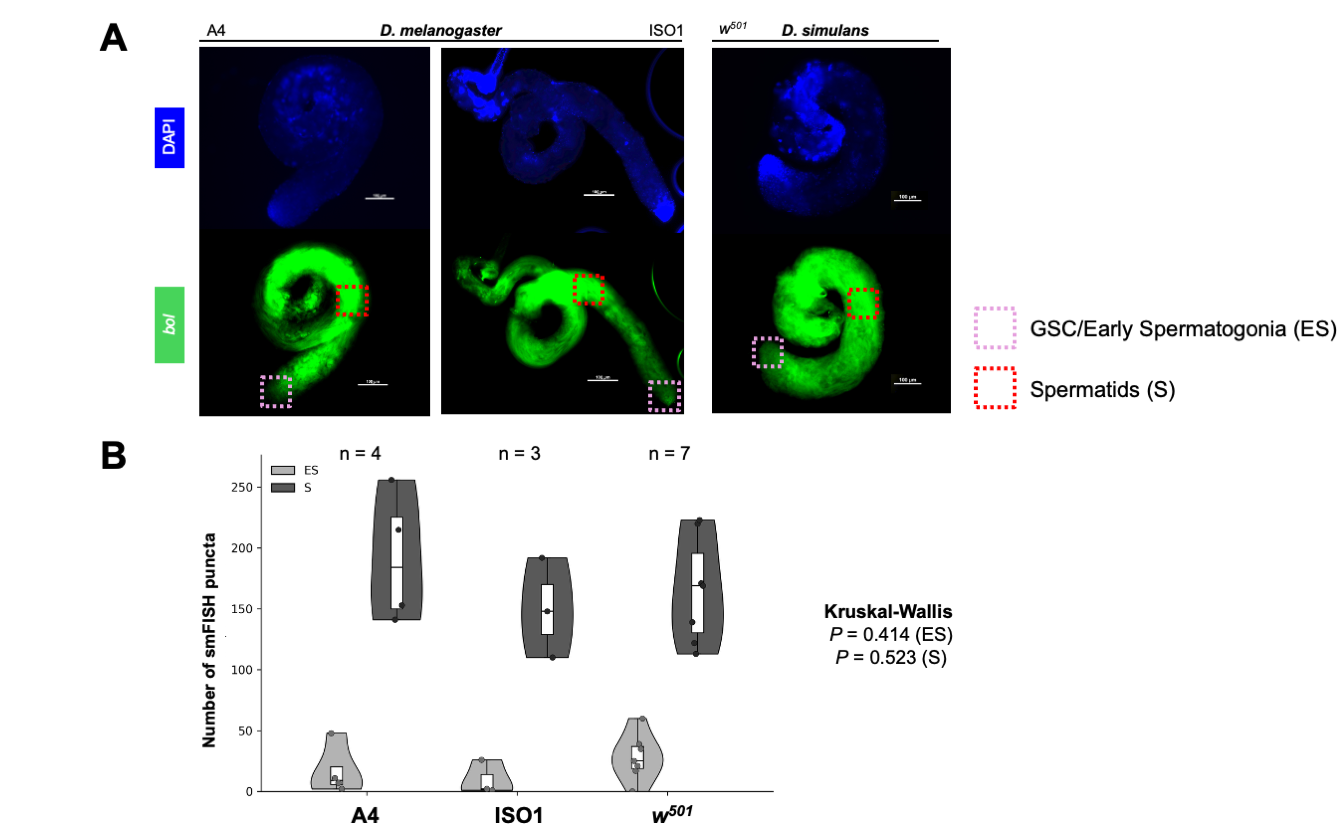

Supplement: S17 Fig — (A) Representative smFISH images of bol mRNA expression in the testis from the strains A4, ISO1, and w501. Pink and red dashed boxes mark the standardized regions of interest (ROIs) used for quantification in the GSC/Early Spermatogonia (ES) and Spermatids (S), respectively. Nuclei were marked with DAPI. Scale bars, 100 µm. (B) smFISH puncta counts quantified within fixed-size ROIs in ES and S. Violin plots show the distribution across images, embedded boxplots show the median and interquartile range, and points represent individual values. Sample sizes are shown above each strain. Kruskal–Wallis tests were performed separately for ES and S. bol signal intensity did not differ significantly across strains in either cell type. The data underlying this figure are provided in S5 Data. (TIFF) [file pbio.3003869.s017.tiff]

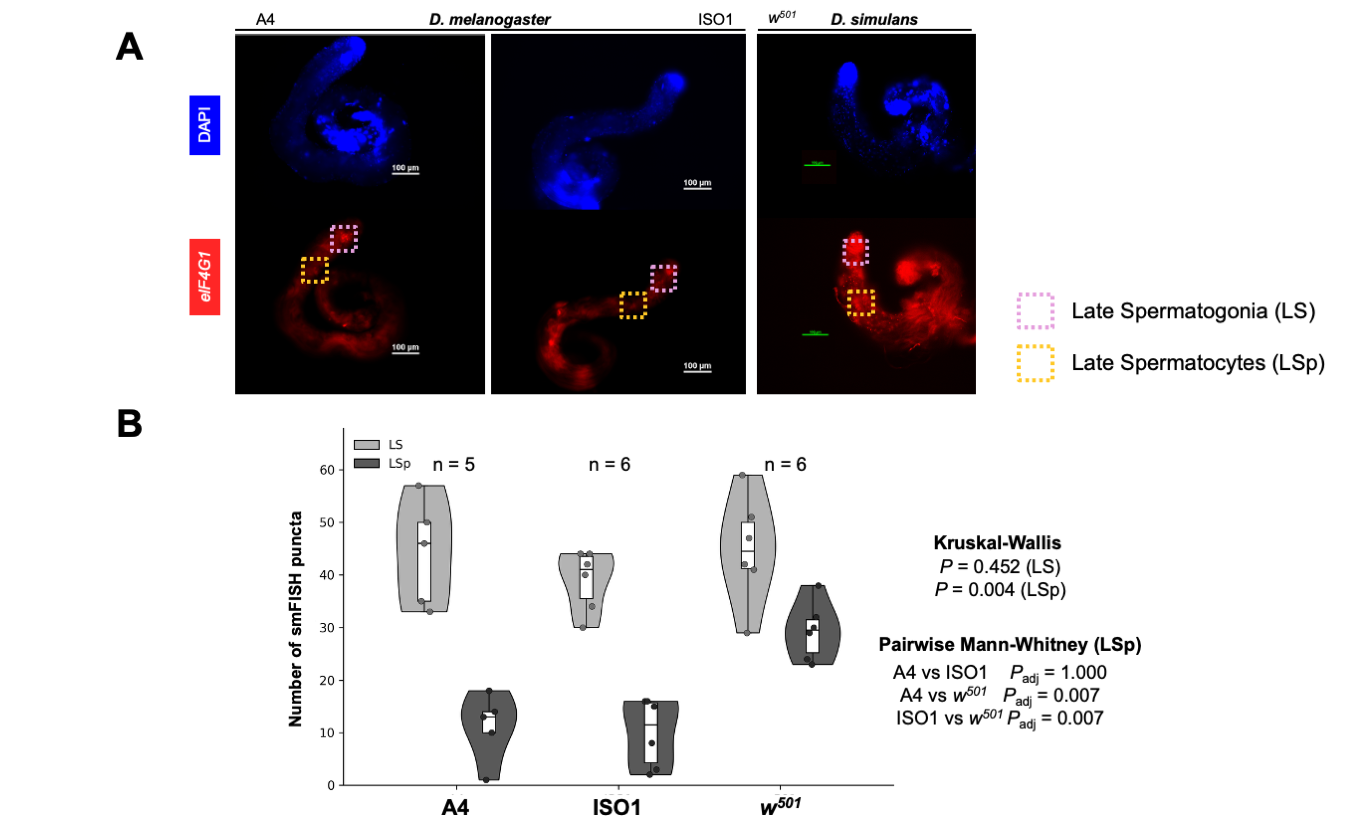

Supplement: S18 Fig — (A) Representative smFISH images of eIF4G1 mRNA expression in the testis from the strains A4, ISO1, and w501. Pink and yellow dashed boxes mark the standardized regions of interest (ROIs) used for quantification in Late Spermatogonia (LS) and Late Spermatocytes (LSp), respectively. Nuclei were marked with DAPI. Scale bars, 100 µm. (B) smFISH puncta counts quantified within fixed-size ROIs in LS and LSp. Violin plots show the distribution across images, embedded boxplots show the median and interquartile range, and points represent individual values. Sample sizes are shown above each strain. Kruskal–Wallis tests were performed separately for LS and LSp, followed by pairwise two-sided Mann–Whitney tests with Benjamini–Hochberg correction for multiple tests in the case of LSp. eIF4G1 signal intensity was similar across strains in LS, whereas in LSp it was significantly elevated in w501 relative to A4 and ISO1. The data underlying this figure are provided in S6 Data. (TIFF) [file pbio.3003869.s018.tiff]

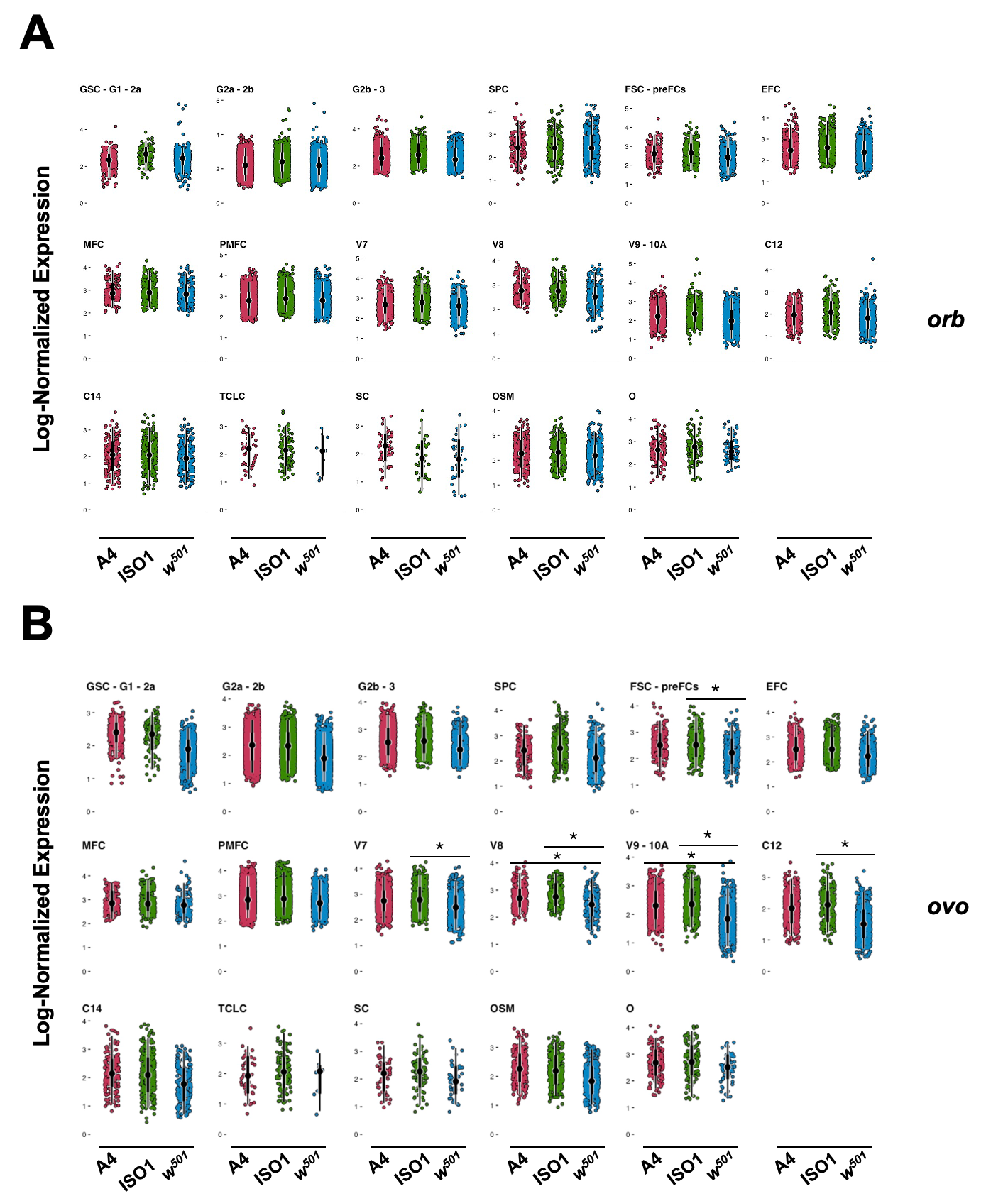

Supplement: S19 Fig — (A) The gene orb shows no differential expression across cell types or strains, suggesting functional constraints operating on mRNA abundance. (B) The gene ovo shows consistent upregulation in the two D. melanogaster strains compared to D. simulans in two cell types (V8, V9-10A), with no intraspecific difference. Cell types: GSC/G1-2a, germline stem cells and germarium region 1 and 2a cells; G2a-2b, germarium region 2a and 2b cells; G2b-3, germarium region 2b and 3 cells; SPC, stalk and polar cells; FSC/preFCs, follicle stem cells and pre-follicle cells; EFC, early follicle cells; MFC, mitotic follicle cells stage 1–5; PMFC, post-mitotic follicle cells stage 6; V7, vitellogenic main-body follicle cells (MBFCs) stage 7; V8, vitellogenic MBFCs stage 8; V9-10A, vitellogenic MBFCs stage 9-10A; C12, choriogenic MBFCs stage 12; C14, choriogenic MBFCs stage 14; TCLC, terminal corpus luteum cells; SC, stretch cells; OSM, ovarian sheath muscle; and O, oviduct. (TIFF) [file pbio.3003869.s019.tiff]

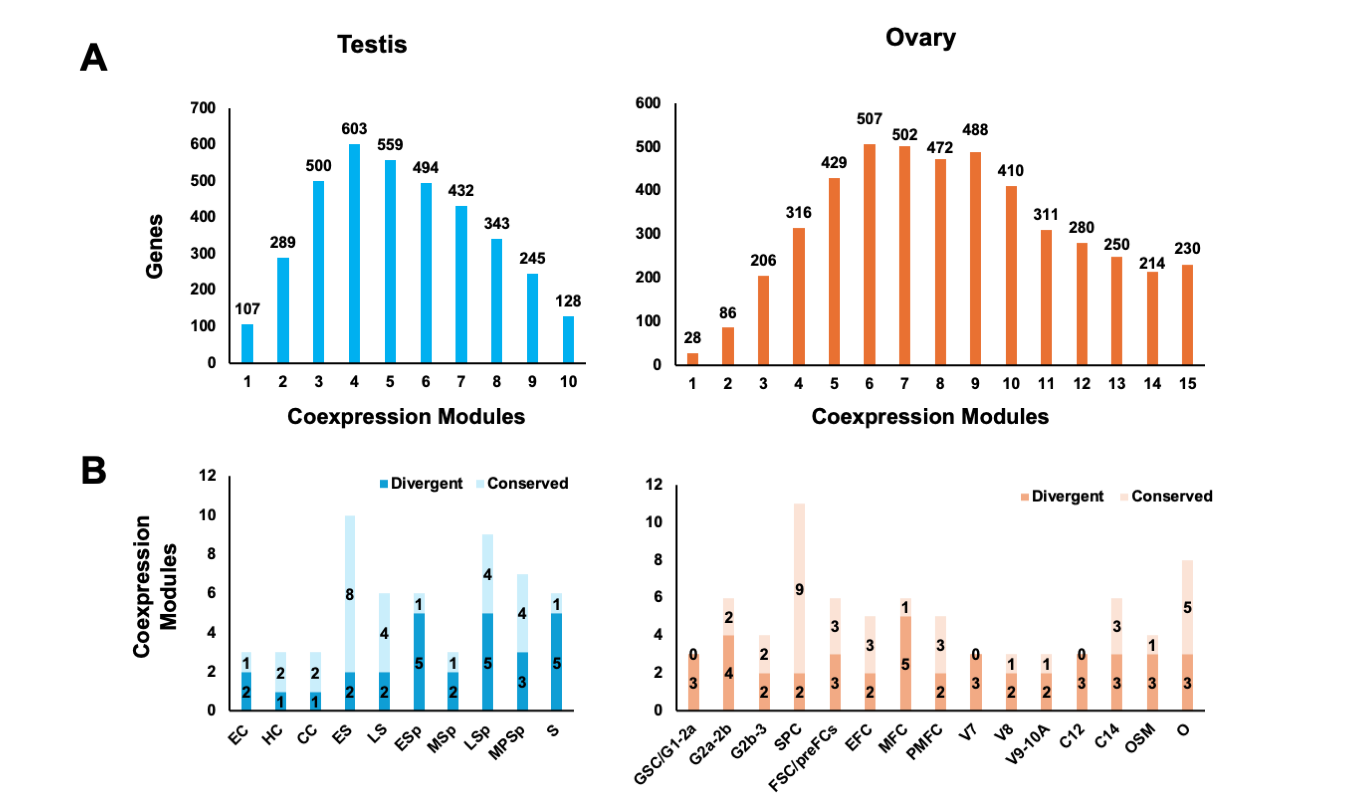

Supplement: S20 Fig — (A) Number of genes present across different coexpression modules. (B) Number of conserved and divergent coexpression modules between D. melanogaster and D. simulans per cell type. Coexpression modules were delineated with hdWGCNA [54]. (TIFF) [file pbio.3003869.s020.tiff]

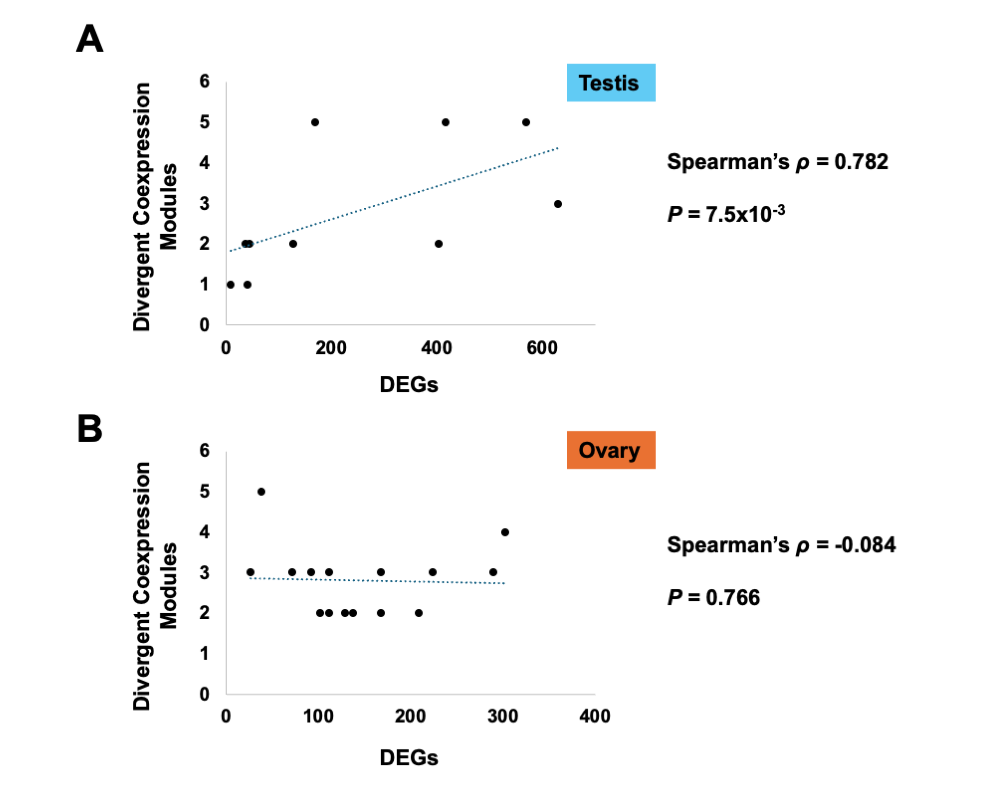

Supplement: S21 Fig — (A, B) Testis and ovary. Divergent coexpression modules between D. melanogaster and D. simulans were delineated with hdWGCNA [54], while differential expression on a gene basis was determined independently at a 1% FDR and a log2 fold-change ≥ |1| for both interspecific contrasts, i.e., A4 versus w501 and ISO1 versus w501. The Spearman’s rho and its statistical significance are provided for each linear relationship. (TIFF) [file pbio.3003869.s021.tiff]

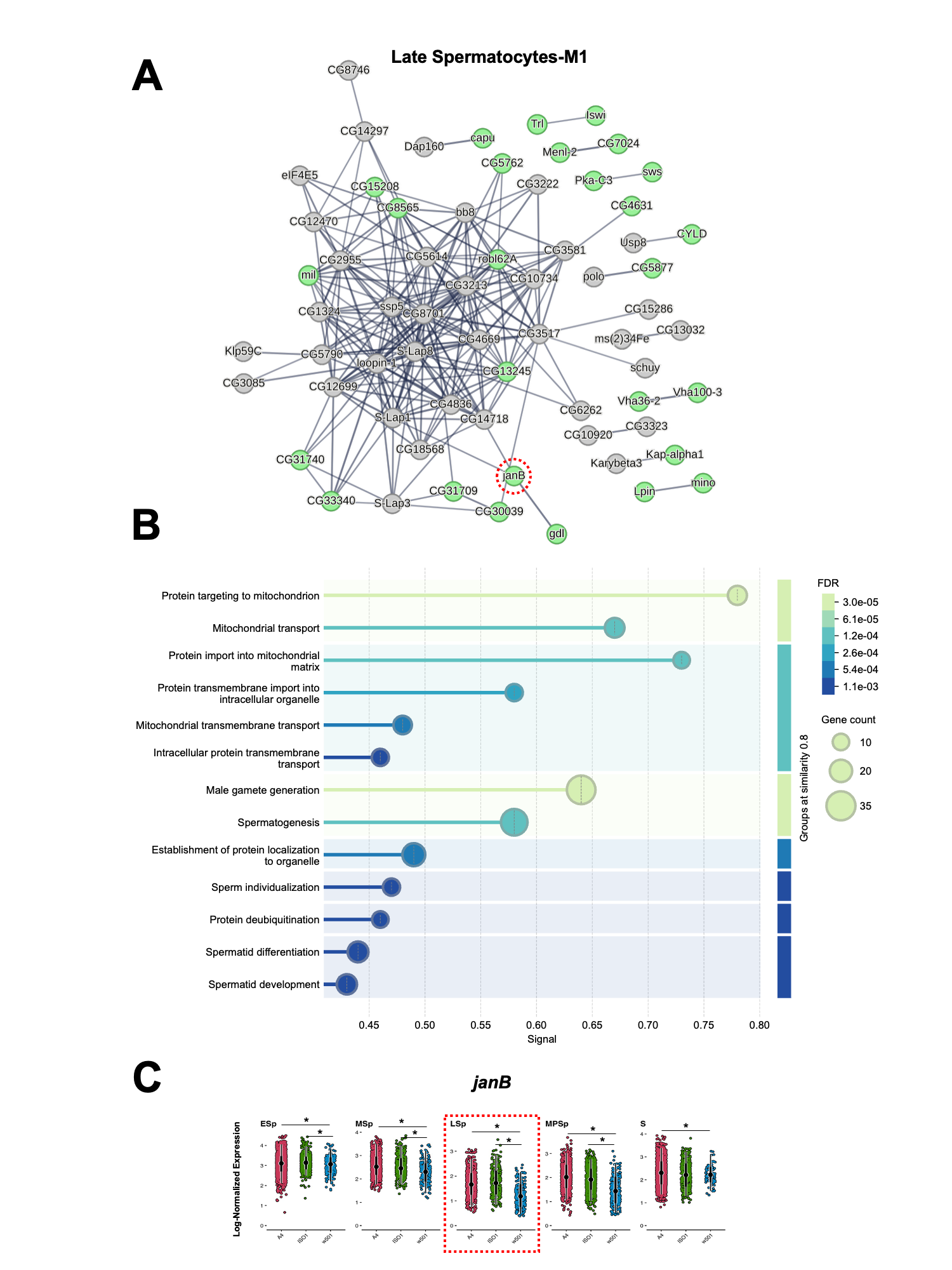

Supplement: S22 Fig — (A) Gene coexpression network structure of the top 160 genes in Module M1 in late spermatocytes (LSp; medium confidence threshold = 0.4; FDR = 5%). Green nodes represent genes that are significantly upregulated in D. melanogaster relative to D. simulans according to our differential expression analysis. Network edges denote the level of confidence and disconnected nodes are omitted. (B) Gene Ontology enrichment results (Biological Process category) for the top 500 genes in Module M1. (C) Single-nucleus RNA-seq expression patterns for one of the genes in this coexpression module, janB, across the meiotic testis cell types (also circled in (A)). This gene is upregulated in D. melanogaster relative to D. simulans in several cell types, including late spermatocytes (LSp). Asterisks denote statistically significant differences in mRNA levels at 1% FDR and a log2 fold-change ≥ |1|. Testis cell types (c): ESp, early spermatocytes; MSp, mid spermatocytes; LSp, late spermatocytes; MPSp, maturing primary spermatocytes; S, spermatids. (TIFF) [file pbio.3003869.s022.tiff]

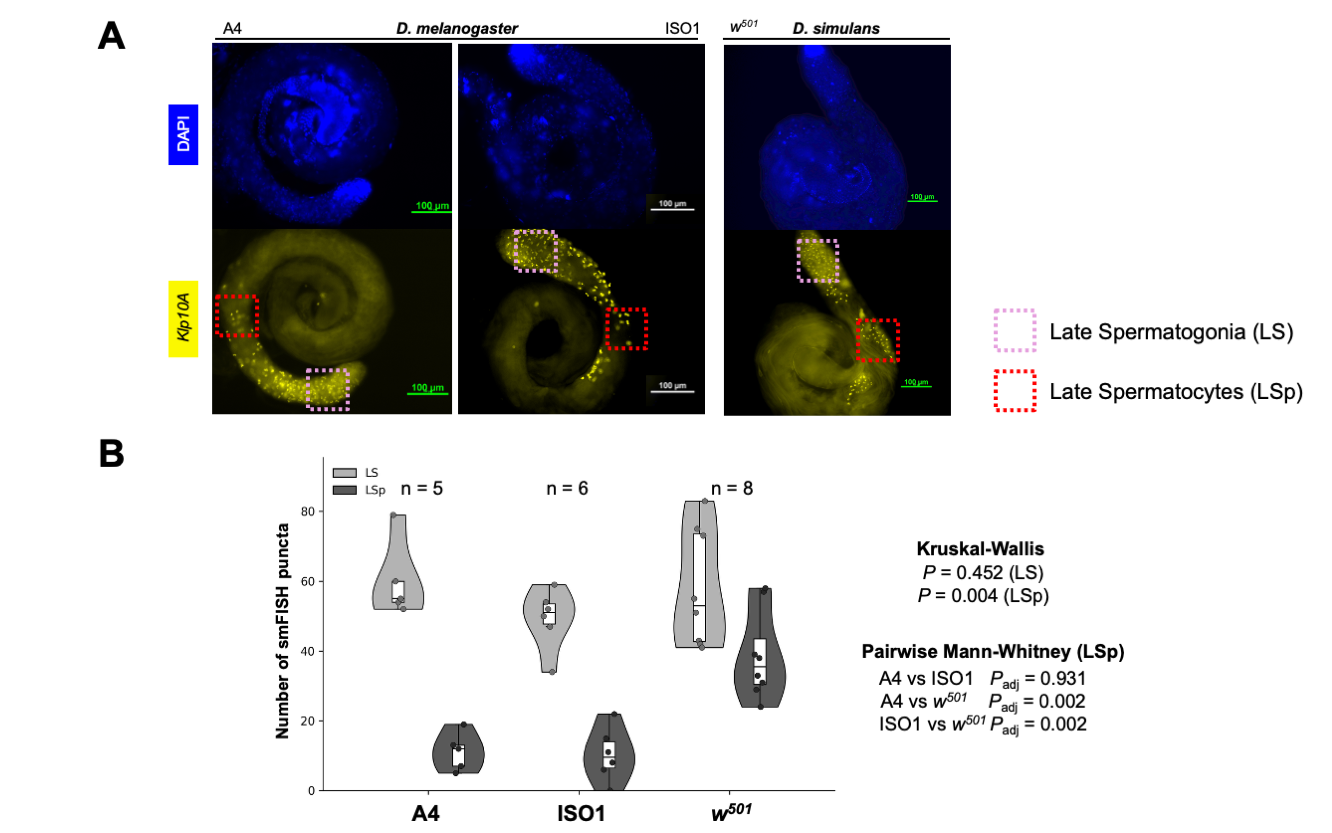

Supplement: S23 Fig — (A) Representative smFISH images of Klp10A mRNA expression in the testis from the strains A4, ISO1, and w501. Pink and red dashed boxes mark the standardized regions of interest (ROIs) used for quantification in Late Spermatogonia (LS) and Late Spermatocytes (LSp), respectively. Nuclei were marked with DAPI. Scale bars, 100 µm. (B) smFISH puncta counts quantified within fixed-size ROIs in LS and LSp. Violin plots show the distribution across images, embedded boxplots show the median and interquartile range, and points represent individual values. Sample sizes are shown above each strain. Kruskal–Wallis tests were performed separately for LS and LSp, followed by pairwise two-sided Mann–Whitney tests with Benjamini–Hochberg correction for LSp. Klp10A signal intensity was similar across strains in LS, whereas in LSp it was significantly elevated in w501 relative to A4 and ISO1. The data underlying this figure are provided in S9 Data. (TIFF) [file pbio.3003869.s023.tiff]

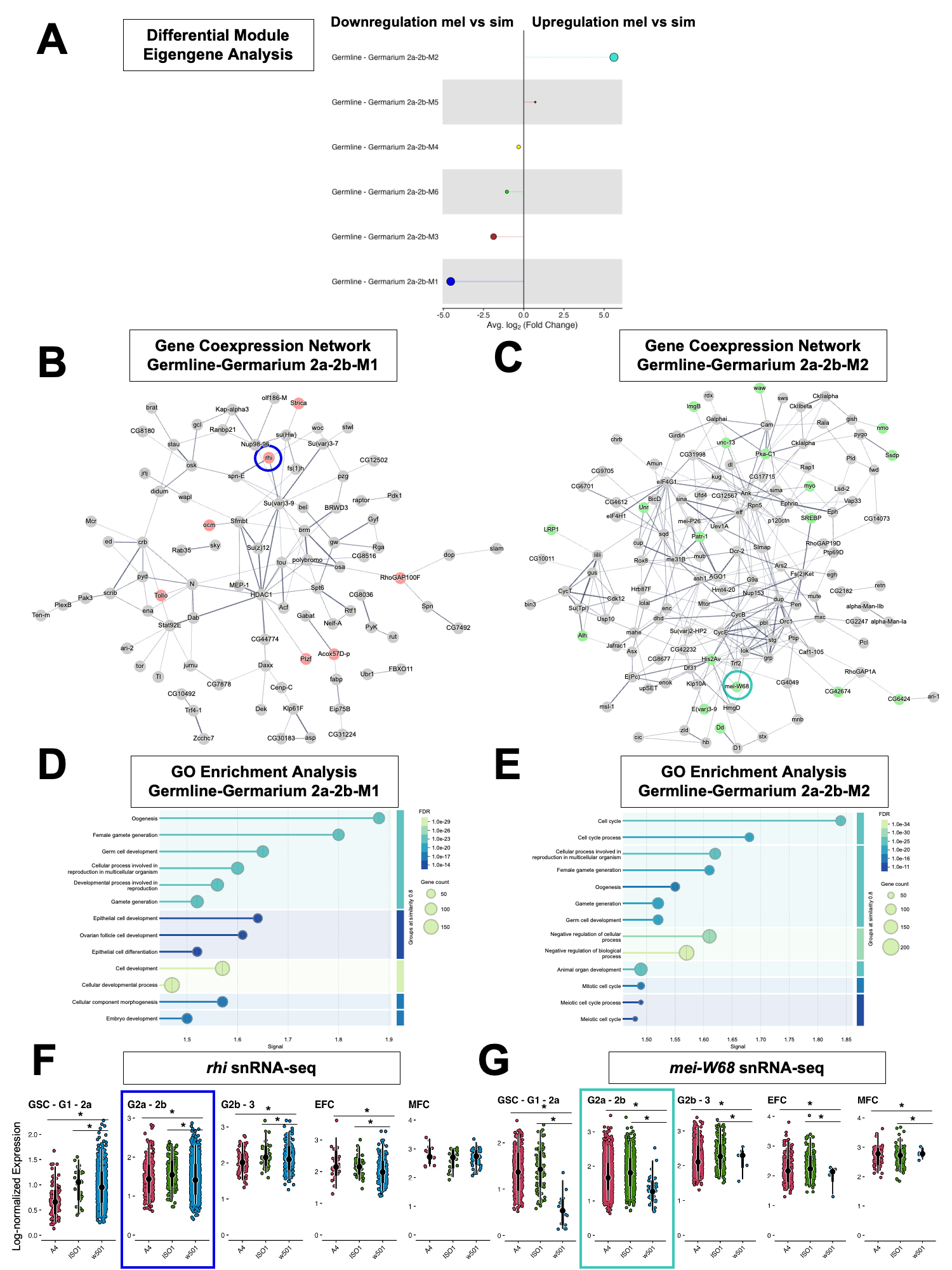

Supplement: S24 Fig — (A) Differential module eigengene (DME) analysis showing log2(fold change) of coexpression module activity between species. Significant interspecific differences affect Modules M1 and M2 (≥100 genes). (B, C) Coexpression network of the top 160 genes in Modules M1 and M2 (medium confidence = 0.4, 5%FDR). Red nodes indicate downregulation in D. melanogaster (M1), while green nodes indicate upregulation in D. melanogaster (M2). Network edges indicate the level of confidence, and disconnected nodes are hidden. (D, E) GO enrichment analysis (Biological Process) for the top 500 genes in Modules M1 and M2. (F, G) Expression levels of the genes rhi (M1) and mei-W68 (M2) across early germline and follicular ovary cell types; the genes are also indicated with blue and turquoise circles in (B) and (C), respectively. The cell type of interest, Germline-Germarium 2a–2b cells, is highlighted. Cell types: GSC/G1-2a, germline stem cells and germarium region 1 and 2a cells; G2a-2b, germarium region 2a and 2b cells; G2b-3, germarium region 2b and 3 cells; EFC, early follicle cells; and MFC, mitotic follicle cells stage 1–5. (TIFF) [file pbio.3003869.s024.tiff]

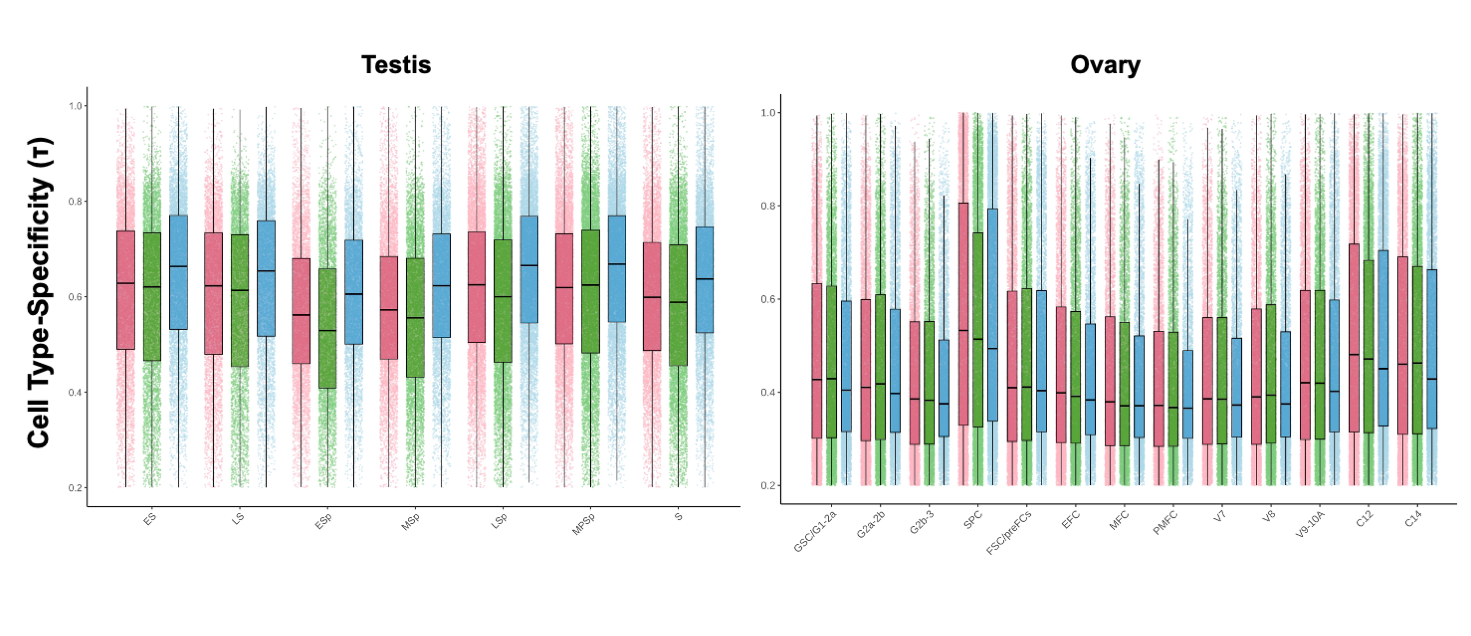

Supplement: S25 Fig — (A, B) Cell-type tau distributions (τ), a measure of intra-tissue expression specificity, across testis and ovary, respectively, for all expressed genes by cell type. Higher τ values indicate more restricted expression across cell types within a given tissue. Only cell types directly involved in gametogenesis and with at least 100 cells per strain and 350 across all strains were included. Genes expressed in at least 1% of each cell type and with a minimum average expression of 0.01 are considered. Boxes represent the interquartile range around the median (black horizontal line) and whiskers extend to 1.5 times the interquartile range. Each point represents the cell-type tau value of a particular gene, and values are grouped by strain and cell type. A4 and ISO1, D. melanogaster strains; w501, D. simulans strain. (TIFF) [file pbio.3003869.s025.tiff]

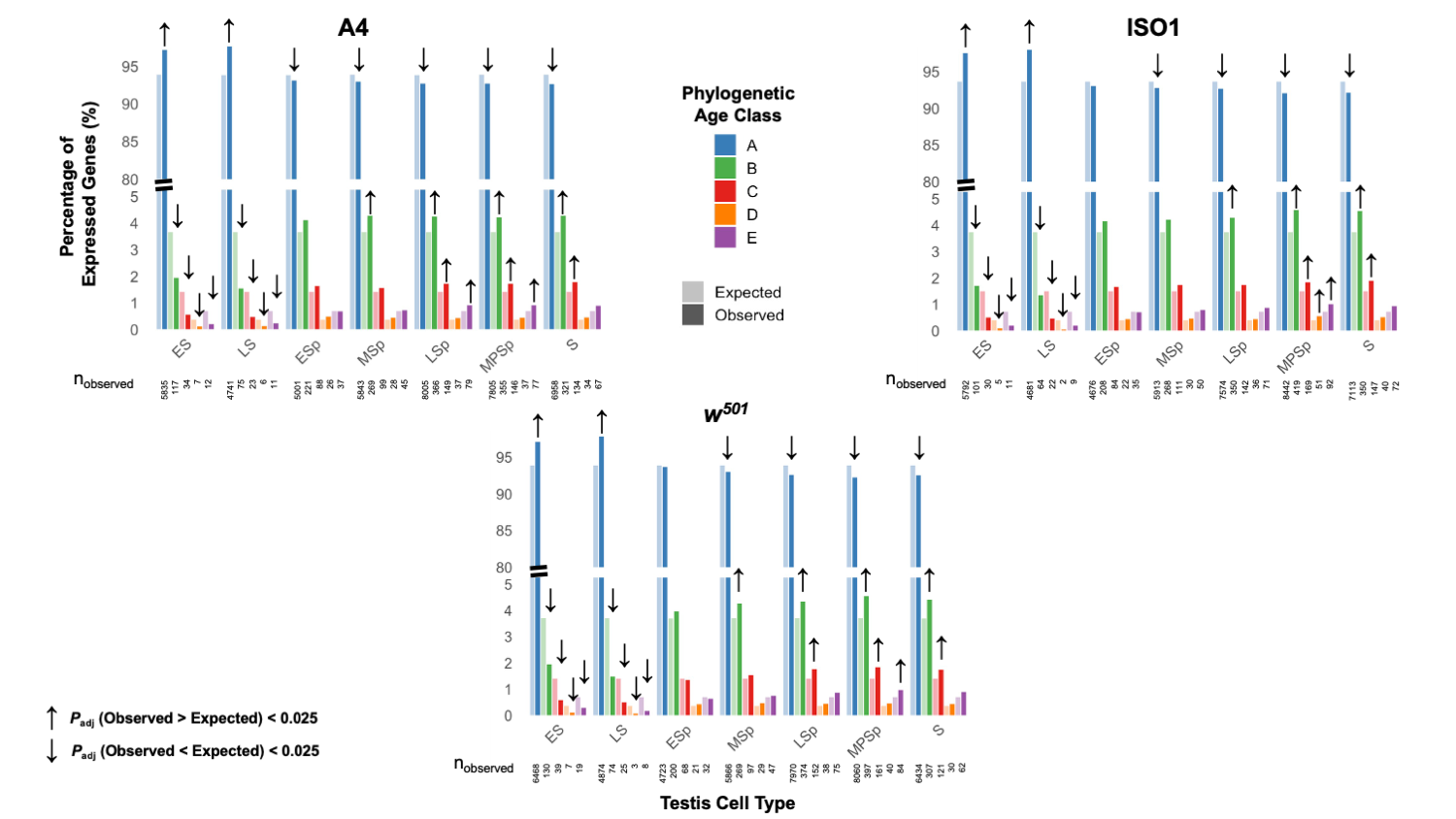

Supplement: S26 Fig — Bar plots show the observed and expected (darker and lighter bars, respectively) percentages of expressed genes in each phylogenetic age class (A–E) across testis cell types for each strain (ISO1 and A4, D. melanogaster; w501, D. simulans). Class A includes the oldest genes and Class E the youngest. Vertical arrows denote statistically significant deviations (↑, enrichment; ↓ , depletion) between observed and expected values based on a permutation test and corrected for multiple tests [139]. Numbers below each cell type indicate the observed counts of expressed genes per age class. The five phylogenetic age classes as defined by Dong and colleagues [78]: class A, genes present before the Drosophila radiation; class B, genes originated in the genus Drosophila; class C, genes originated in the Sophophora subgenus; class D, genes originated in the melanogaster species group; class E, genes originated in the D. melanogaster species subgroup comprising the simulans species complex and D. melanogaster. Cell types: EC, epithelial cells; HC, hub cells; CC, cyst cells; ES, germline stem cells and early spermatogonia; LS, late spermatogonia; ESp, early spermatocytes; MSp, mid spermatocytes; LSp, late spermatocytes; MPSp, maturing primary spermatocytes; and S, spermatids. (TIFF) [file pbio.3003869.s026.tiff]

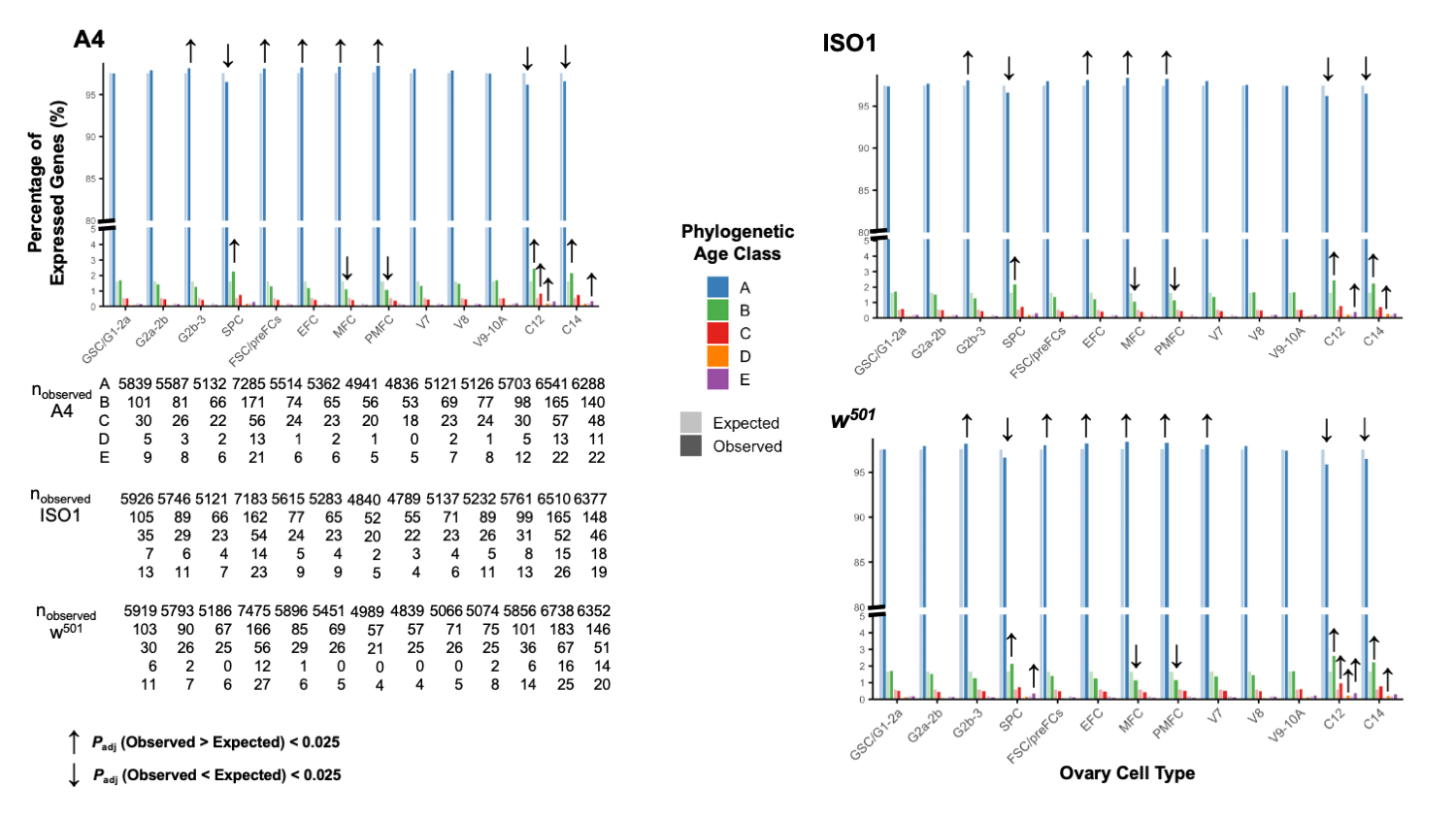

Supplement: S27 Fig — Bar plots show the observed and expected (darker and lighter bars, respectively) percentages of expressed genes in each phylogenetic age class (A–E) across ovary cell types for each strain (ISO1 and A4, D. melanogaster; w501, D. simulans). Class A includes the oldest genes and Class E the youngest. Vertical arrows denote statistically significant deviations (↑, enrichment; ↓ , depletion) between observed and expected values based on a permutation test and corrected for multiple tests [139]. Numbers below each cell type indicate the observed counts of expressed genes per age class. The five phylogenetic age classes as defined by Dong and colleagues [78]: class A, genes present before the Drosophila radiation; class B, genes originated in the genus Drosophila; class C, genes originated in the Sophophora subgenus; class D, genes originated in the melanogaster species group; class E, genes originated in the D. melanogaster species subgroup comprising the simulans species complex and D. melanogaster. Cell types: GSC/G1-2a, germline stem cells and germarium region 1 and 2a cells; G2a-2b, germarium region 2a and 2b cells; G2b-3, germarium region 2b and 3 cells; SPC, stalk and polar cells; FSC/preFCs, follicle stem cells and pre-follicle cells; EFC, early follicle cells; MFC, mitotic follicle cells stage 1–5; PMFC, post-mitotic follicle cells stage 6; V7, vitellogenic main-body follicle cells (MBFCs) stage 7; V8, vitellogenic MBFCs stage 8; V9-10A, vitellogenic MBFCs stage 9-10A; C12, choriogenic MBFCs stage 12; C14, choriogenic MBFCs stage 14; TCLC, terminal corpus luteum cells; SC, stretch cells; OSM, ovarian sheath muscle; and O, oviduct. (TIFF) [file pbio.3003869.s027.tiff]

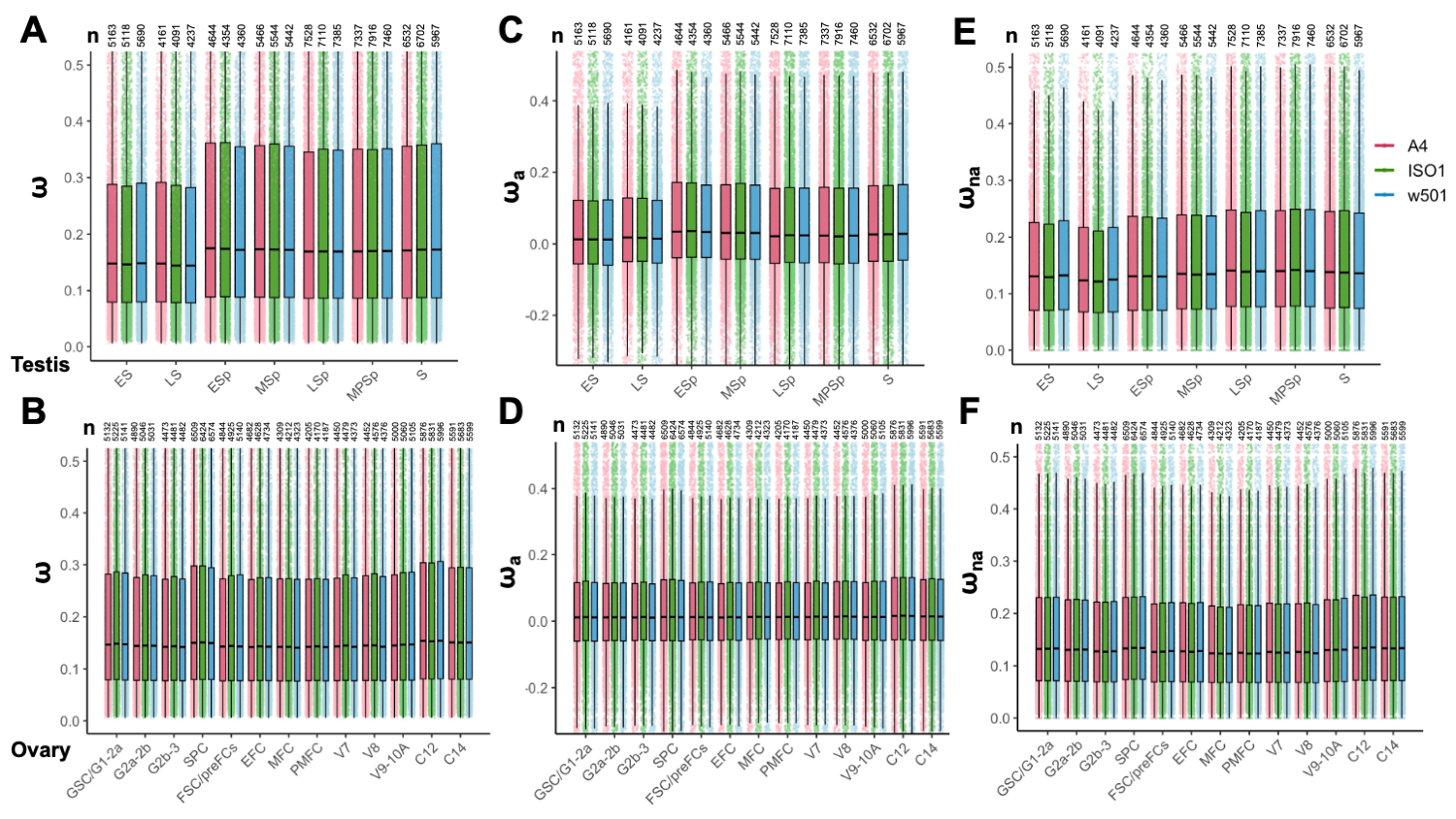

Supplement: S28 Fig — Box plots displaying three metrics of evolutionary change at sequence level of the genes expressed across cell types and strains (A4 and ISO1, D. melanogaster; w501, D. simulans). The plots show: (A, B) the ratio of nonsynonymous to synonymous substitutions (ω, left); (C, D) the adaptive component of the ratio of protein evolution (ωa, center); and (E, F) the nonadaptive component of the rate of protein evolution (ωna, right). Testis, top; ovary, bottom. Boxes represent the interquartile range around the median (black horizontal line) and whiskers extend to 1.5 times the IQR. Each point represents the value of a particular gene, and values are grouped by strain and cell type. Gene counts for each combination (n) are shown above each boxplot. Testis cell types: EC, epithelial cells; HC, hub cells; CC, cyst cells; ES, germline stem cells and early spermatogonia; LS, late spermatogonia; ESp, early spermatocytes; MSp, mid spermatocytes; LSp, late spermatocytes; MPSp, maturing primary spermatocytes; and S, spermatids. Ovary cell types: GSC/G1-2a, germline stem cells and germarium region 1 and 2a cells; G2a-2b, germarium region 2a and 2b cells; G2b-3, germarium region 2b and 3 cells; SPC, stalk and polar cells; FSC/preFCs, follicle stem cells and pre-follicle cells; EFC, early follicle cells; MFC, mitotic follicle cells stage 1–5; PMFC, post-mitotic follicle cells stage 6; V7, vitellogenic main-body follicle cells (MBFCs) stage 7; V8, vitellogenic MBFCs stage 8; V9-10A, vitellogenic MBFCs stage 9-10A; C12, choriogenic MBFCs stage 12; C14, choriogenic MBFCs stage 14; TCLC, terminal corpus luteum cells; SC, stretch cells; OSM, ovarian sheath muscle; and O, oviduct. (TIFF) [file pbio.3003869.s028.tiff]

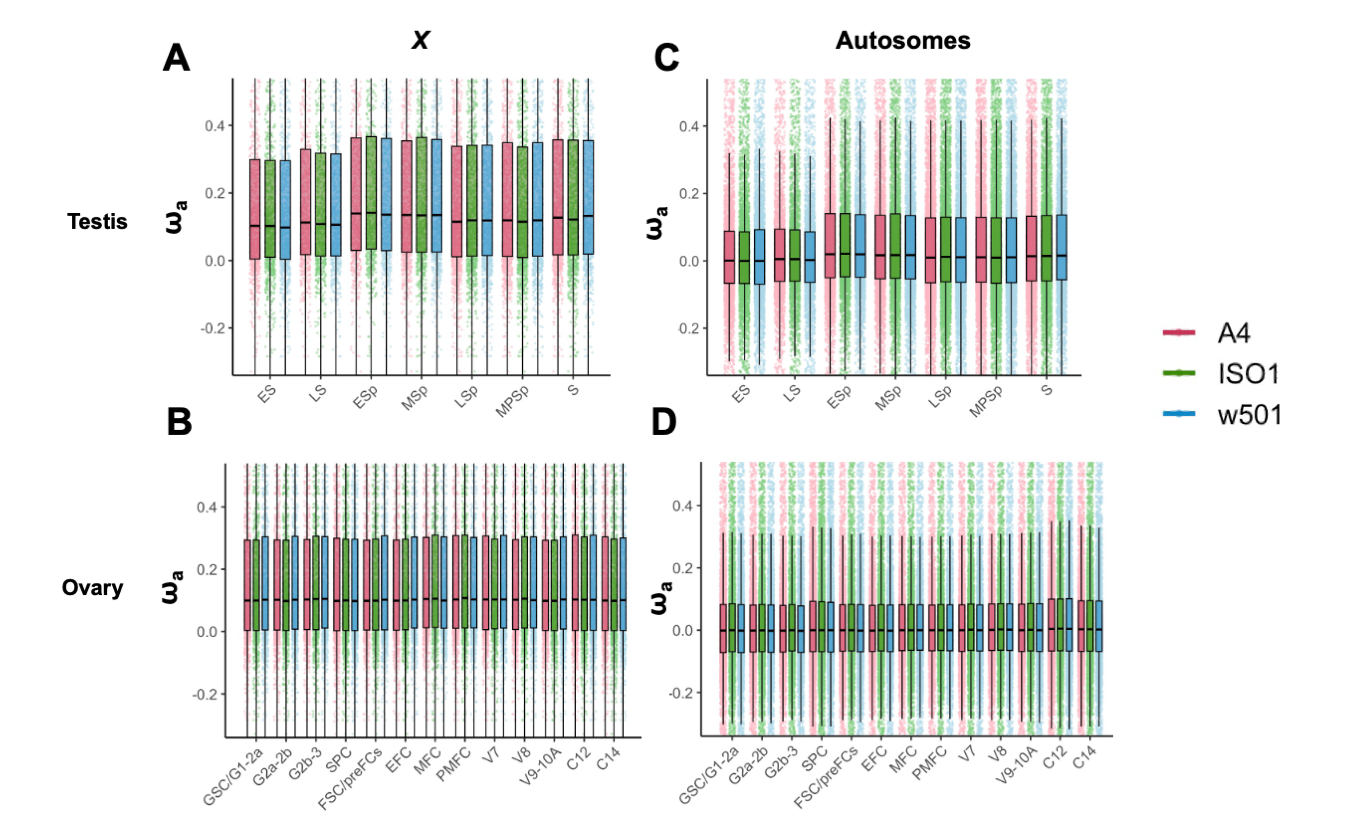

Supplement: S29 Fig — Box plots displaying the adaptive component of the ratio of protein evolution (ωa) at sequence level for the genes expressed across cell types and strains (A4 and ISO1, D. melanogaster; w501, D. simulans) split by (A, B) X-linked and (C, D) autosomal genes in the testis (top) and ovary (bottom). Boxes represent the interquartile range around the median (black horizontal line) and whiskers extend to 1.5 times the IQR. Each point represents the ωa value of a particular gene, and values are grouped by strain and cell type. Testis cell types: EC, epithelial cells; HC, hub cells; CC, cyst cells; ES, germline stem cells and early spermatogonia; LS, late spermatogonia; ESp, early spermatocytes; MSp, mid spermatocytes; LSp, late spermatocytes; MPSp, maturing primary spermatocytes; and S, spermatids. Ovary cell types: GSC/G1-2a, germline stem cells and germarium region 1 and 2a cells; G2a-2b, germarium region 2a and 2b cells; G2b-3, germarium region 2b and 3 cells; SPC, stalk and polar cells; FSC/preFCs, follicle stem cells and pre-follicle cells; EFC, early follicle cells; MFC, mitotic follicle cells stage 1–5; PMFC, post-mitotic follicle cells stage 6; V7, vitellogenic main-body follicle cells (MBFCs) stage 7; V8, vitellogenic MBFCs stage 8; V9-10A, vitellogenic MBFCs stage 9-10A; C12, choriogenic MBFCs stage 12; C14, choriogenic MBFCs stage 14; TCLC, terminal corpus luteum cells; SC, stretch cells; OSM, ovarian sheath muscle; and O, oviduct. (TIFF) [file pbio.3003869.s029.tiff]

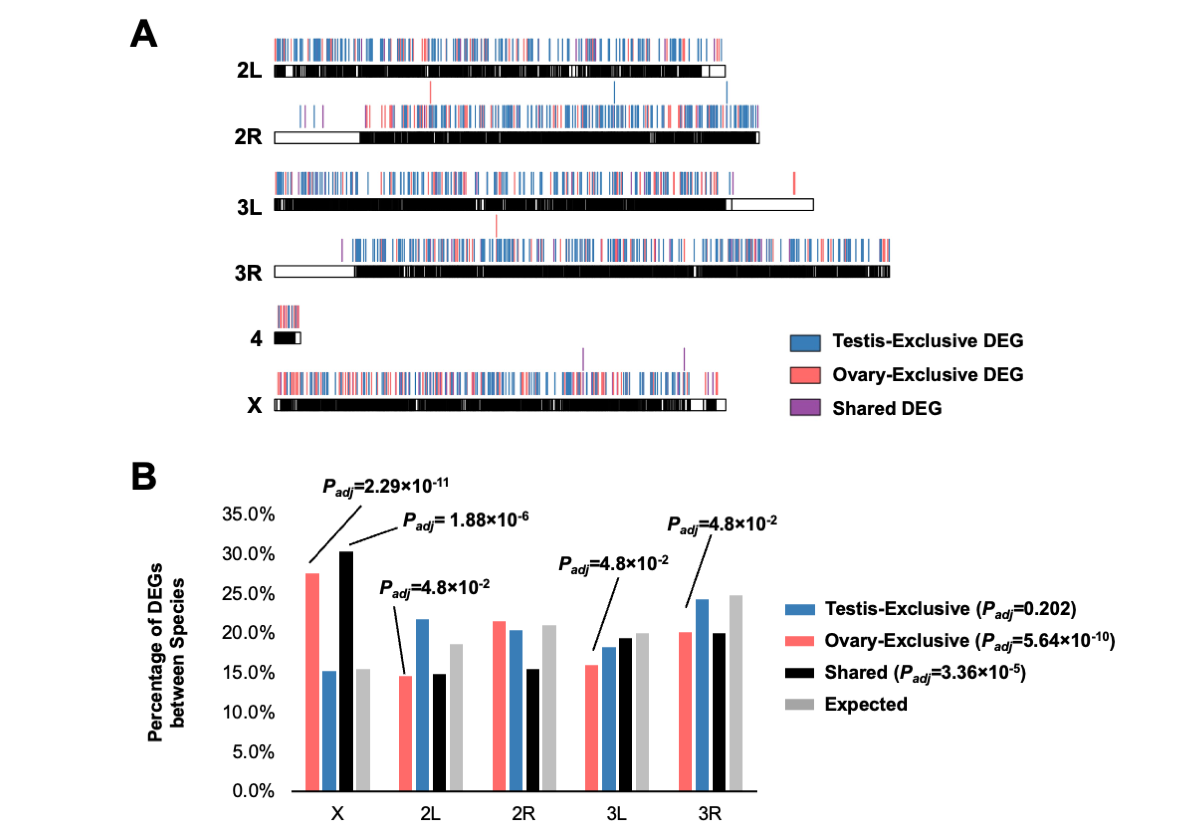

Supplement: S30 Fig — (A) Chromosome distribution of DEGs between species. Only genes that showed consistent expression differences across the two interspecific comparisons (i.e., A4 versus w501 and ISO1 versus w501) for at least one same cell type and not located on the dot-like chromosome 4, are considered. DEGs only in testis, n = 842, only in ovary, n = 431, in both tissues, n = 155. The karyotype was generated using the R package karyoploteR [148]. (B) Percentage of expected versus observed DEGs for each category (testis-exclusive, ovary-exclusive, shared by both tissues) across different chromosomes. The probability of finding a difference between both distributions was determined by a goodness-of-fit chi-squared test (in parenthesis). Post-hoc tests revealed which chromosome contributes to the global statistical difference found. P-values were adjusted for multiple tests correction [139]. (TIFF) [file pbio.3003869.s030.tiff]

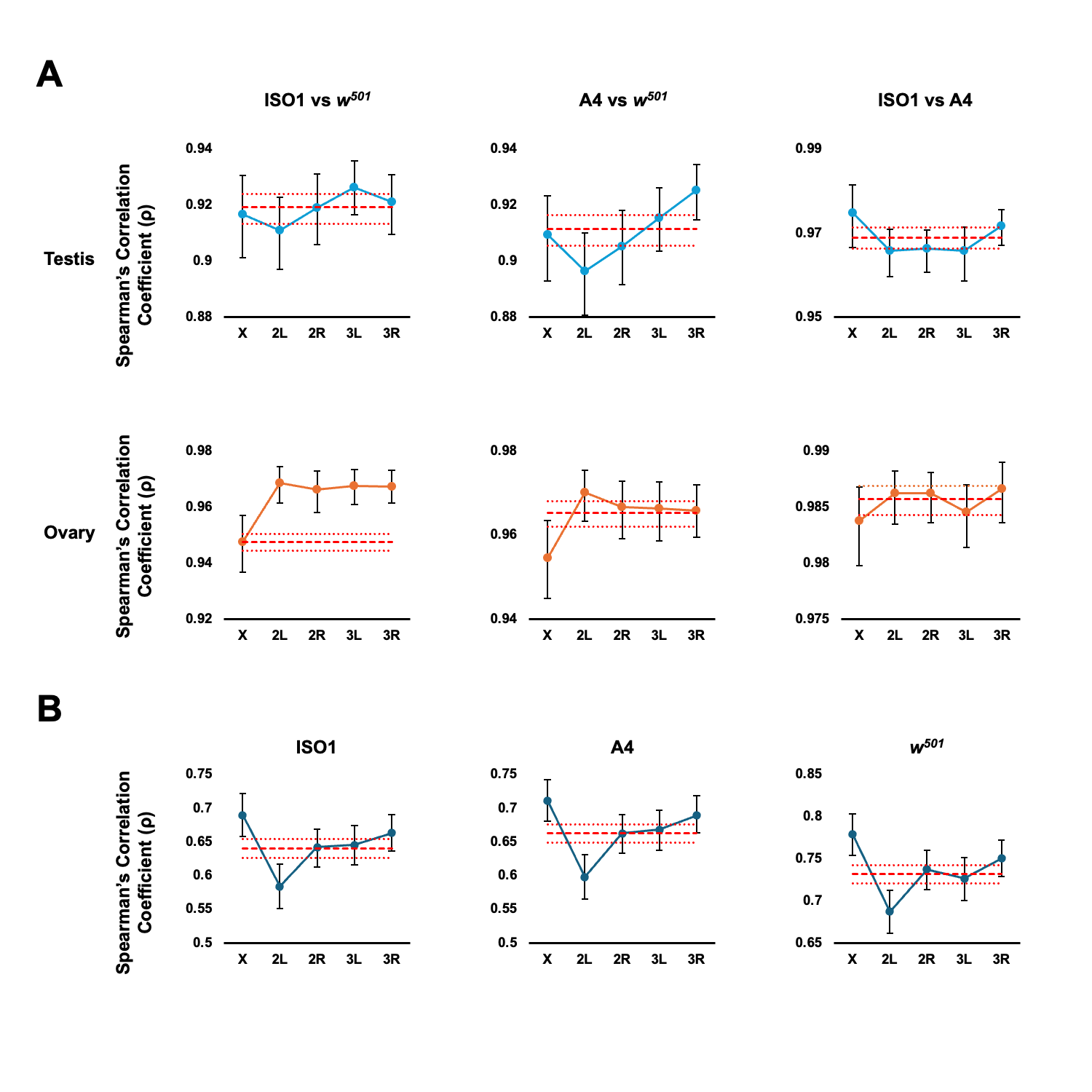

Supplement: S31 Fig — (A) Pairwise Spearman’s Rho correlations of gene expression across chromosomes are shown for three strain comparisons and two tissues, testis and ovary. In the ovary but not in the testis, there is evidence of faster-X evolution in gene expression (i.e., lower correlation) in the two interspecific contrasts. For each graph and chromosome, the dot corresponds to the correlation value and the error bar is the 95% CI. In each graph, the genome-wide correlation is shown as a red dashed line while the red dotted lines correspond to the genome-wide 95% CI. Only the four rod-like autosomes (the left and right arms -L and R- of chromosomes 2 and 3) are considered. (B) Spearman’s Rho correlations of gene expression across chromosomes between expression levels in testis and ovaries are shown for three strains. Gene expression on the X chromosome is not subject to more relaxed functional constraints compared to the autosomes as shown by the highest correlation values of the X versus the autosomes. Strains: A4 and ISO1, D. melanogaster; w501, D. simulans. (TIFF) [file pbio.3003869.s031.tiff]

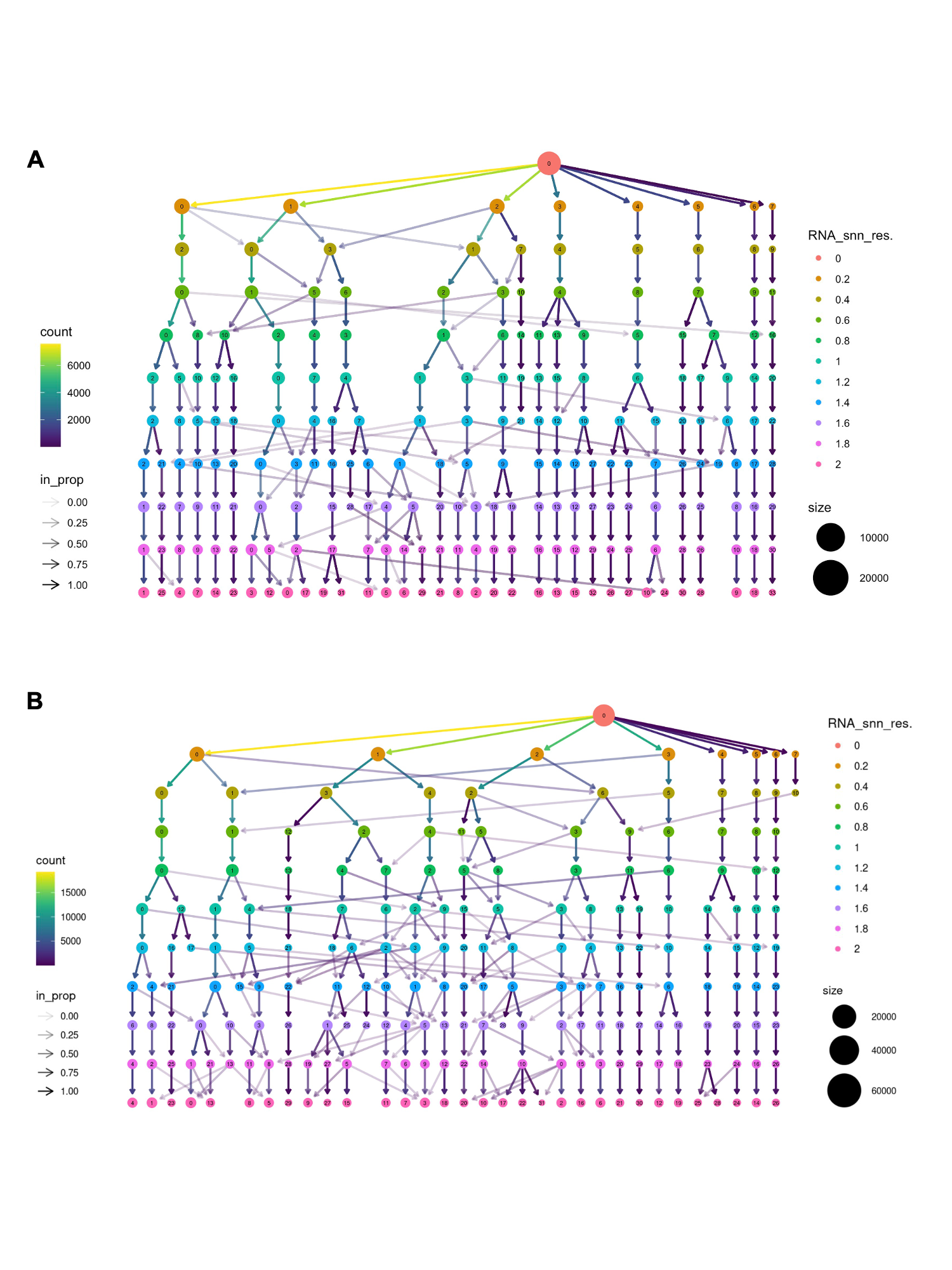

Supplement: S32 Fig — Clustree plots show the relationships among clusters across increasing clustering resolutions (0–2.0, in increments of 0.2) for (A) testis and (B) ovary. Each node represents a cluster identified at a given resolution, and edges connect clusters across adjacent resolutions based on shared cells. These plots were used to assess cluster stability and subdivision across resolutions and to guide selection of an appropriate resolution for downstream annotation and analysis. (TIFF) [file pbio.3003869.s032.tiff]
