# Supplementary material for: Comparative single-nucleus transcriptomics reveals asymmetric evolution of the Drosophila male and female germlines
Source: PLoS Biol. 2026 Jul 20;24(7):e3003869. doi: 10.1371/journal.pbio.3003869 (PMC13384527; doi:10.1371/journal.pbio.3003869)
Supplement: S1 Text — (DOCX) [file pbio.3003869.s033.docx]

**S1 Text. Analysis of cell type proportions across strains**

We examined the extent to which the proportions of different cell types were conserved across species. First, we found that cell type proportions were highly correlated across pairwise comparisons in both tissues (*P*_adj_<1×10^-3^ in all cases; S6 Table). To further test for differences, we chose one of the strains randomly as a reference (A4) and implemented linear models to compare the relationships between the cell type proportions in that strain relative to the other two strains, ISO1 and *w^501^*. Specifically, we tested whether the regression lines differed in their intercept and slope (S5 Table; S3 Fig). For both the testis and ovary, we found no significant differences, indicating that the strains analyzed showed proportional scaling across cell types for both tissues.
